# Supplementary figures and images for: From genes to reproductive health: Immune cell influences on abortion
Source: PLoS One. 2024 Oct 10;19(10):e0309088. doi: 10.1371/journal.pone.0309088 (PMC11466425; doi:10.1371/journal.pone.0309088)

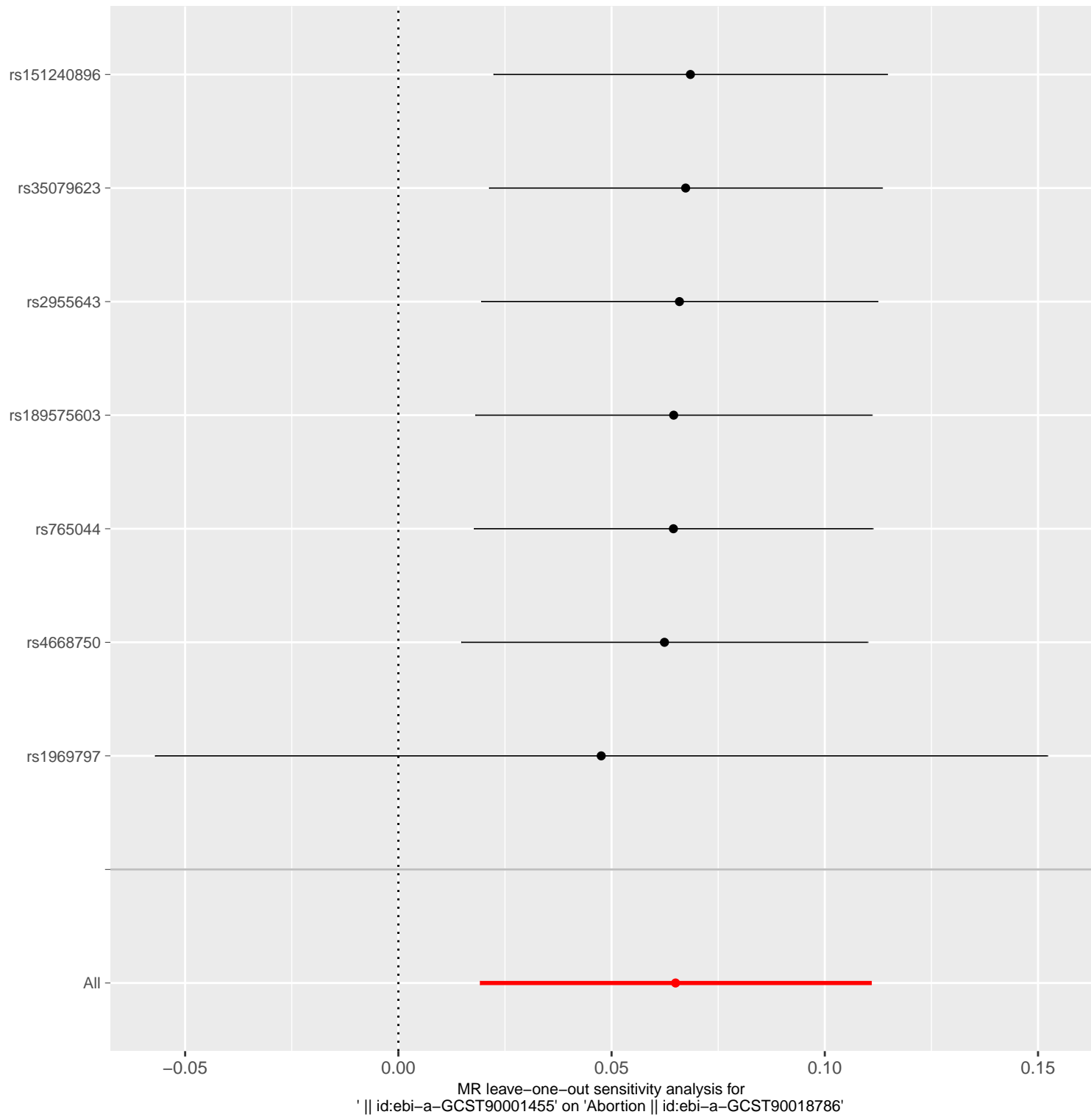

Supplement: S1 File — (ZIP) [file pone.0309088.s001.zip › S1 Fig /ebi-a-GCST90001455/sensitivity-analysis.pdf]

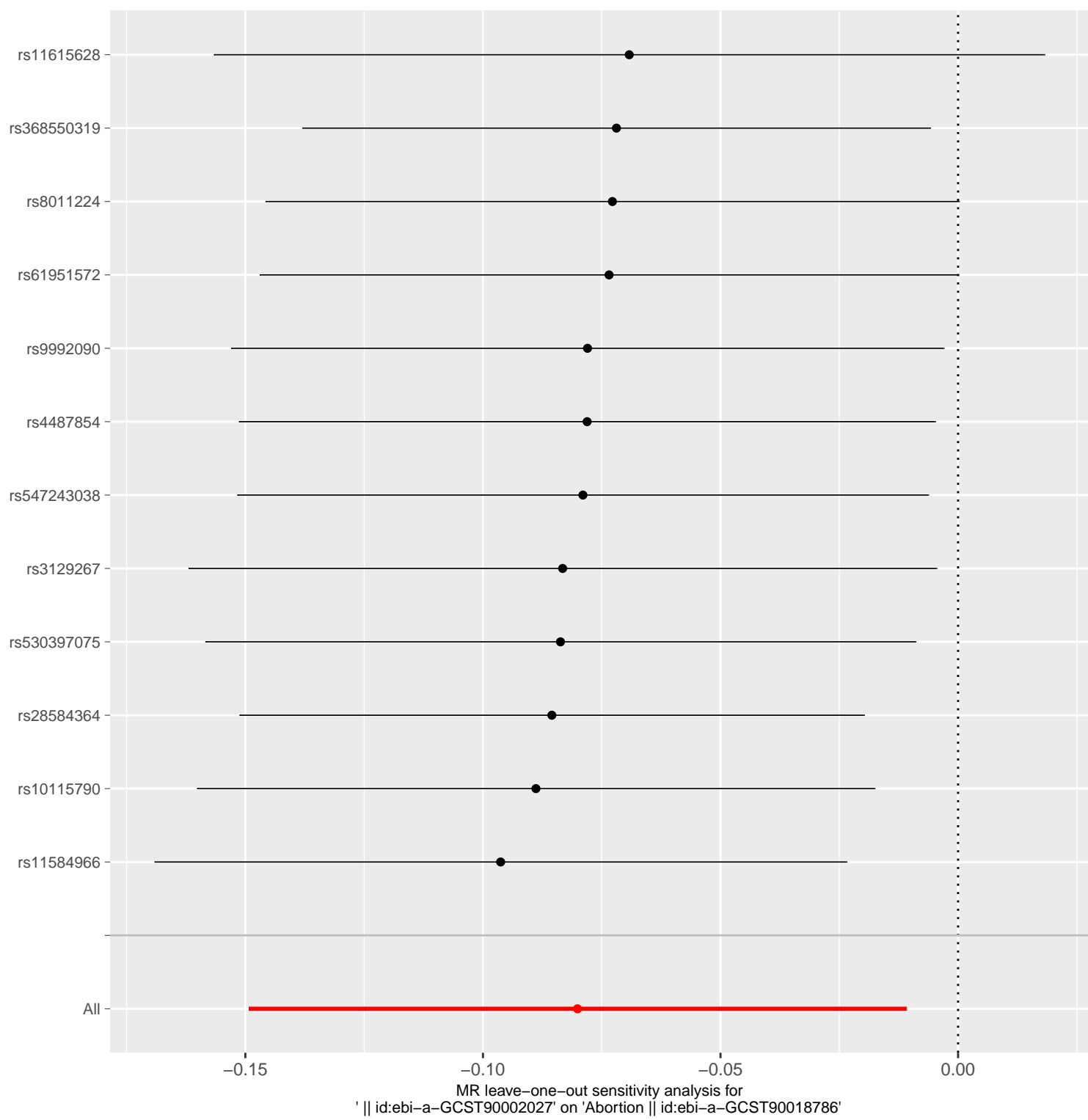

Supplement: S1 File — (ZIP) [file pone.0309088.s001.zip › S1 Fig /ebi-a-GCST90002027/sensitivity-analysis.pdf]

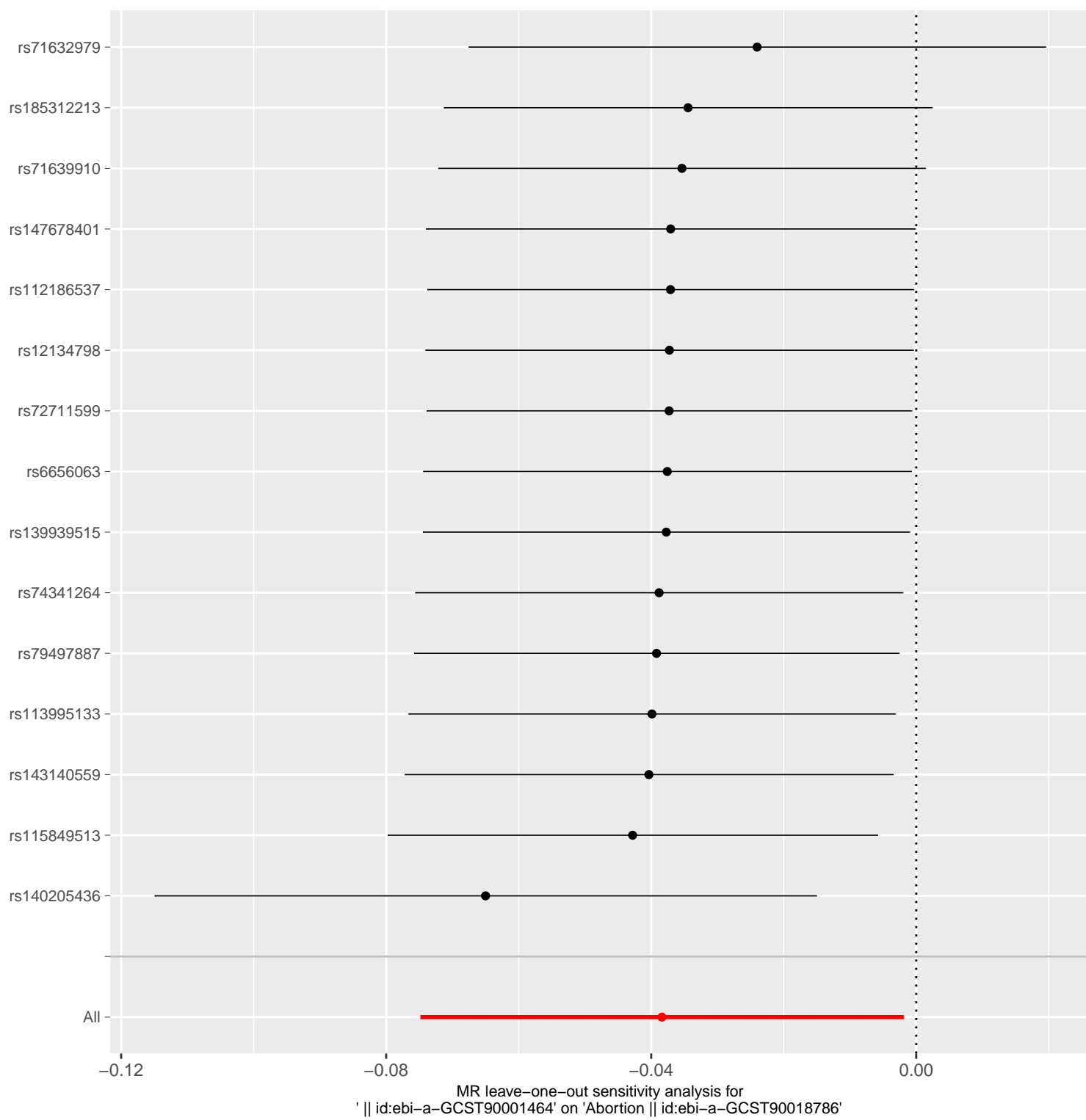

Supplement: S1 File — (ZIP) [file pone.0309088.s001.zip › S1 Fig /ebi-a-GCST90001464/sensitivity-analysis.pdf]

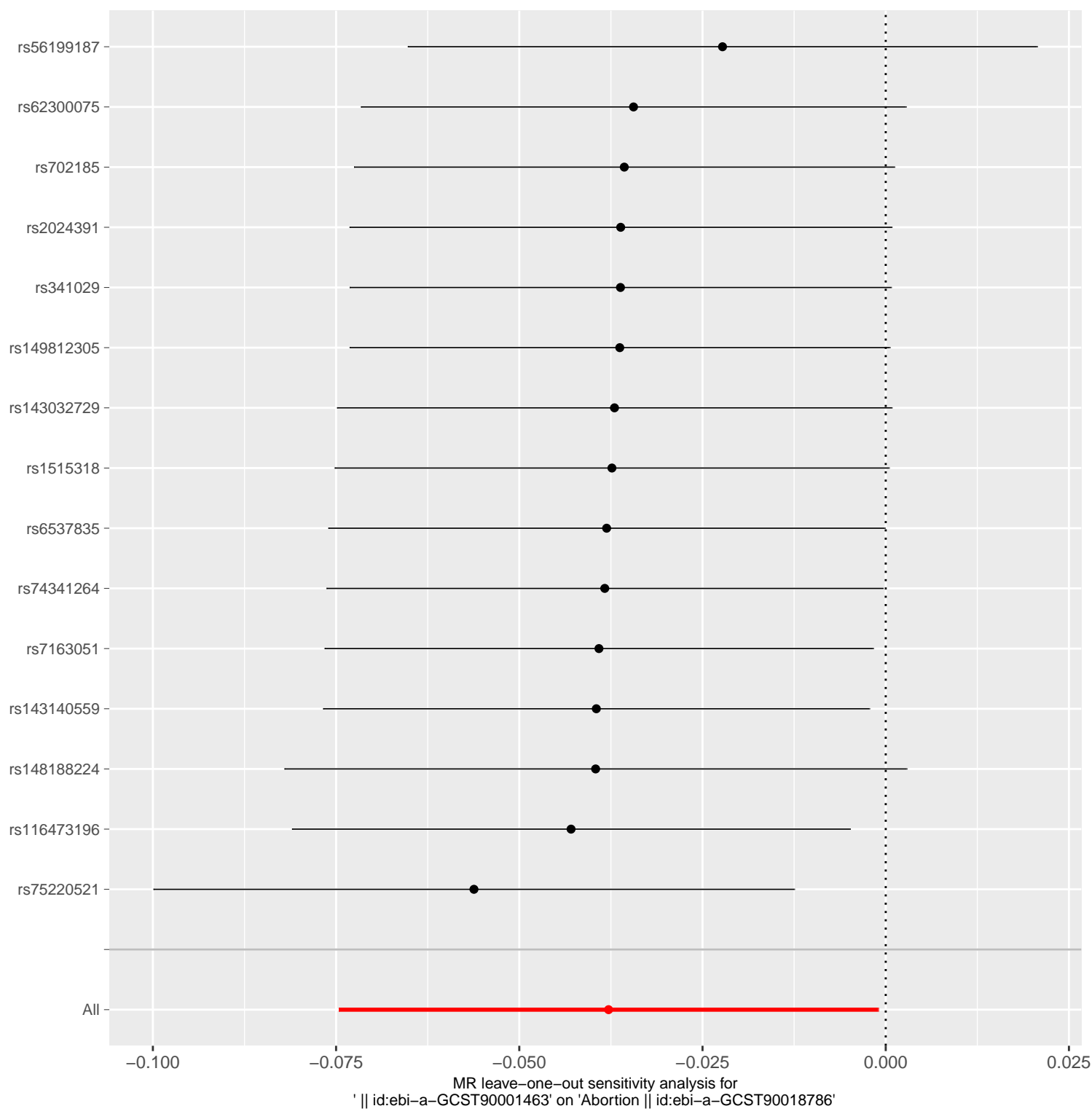

Supplement: S1 File — (ZIP) [file pone.0309088.s001.zip › S1 Fig /ebi-a-GCST90001463/sensitivity-analysis.pdf]

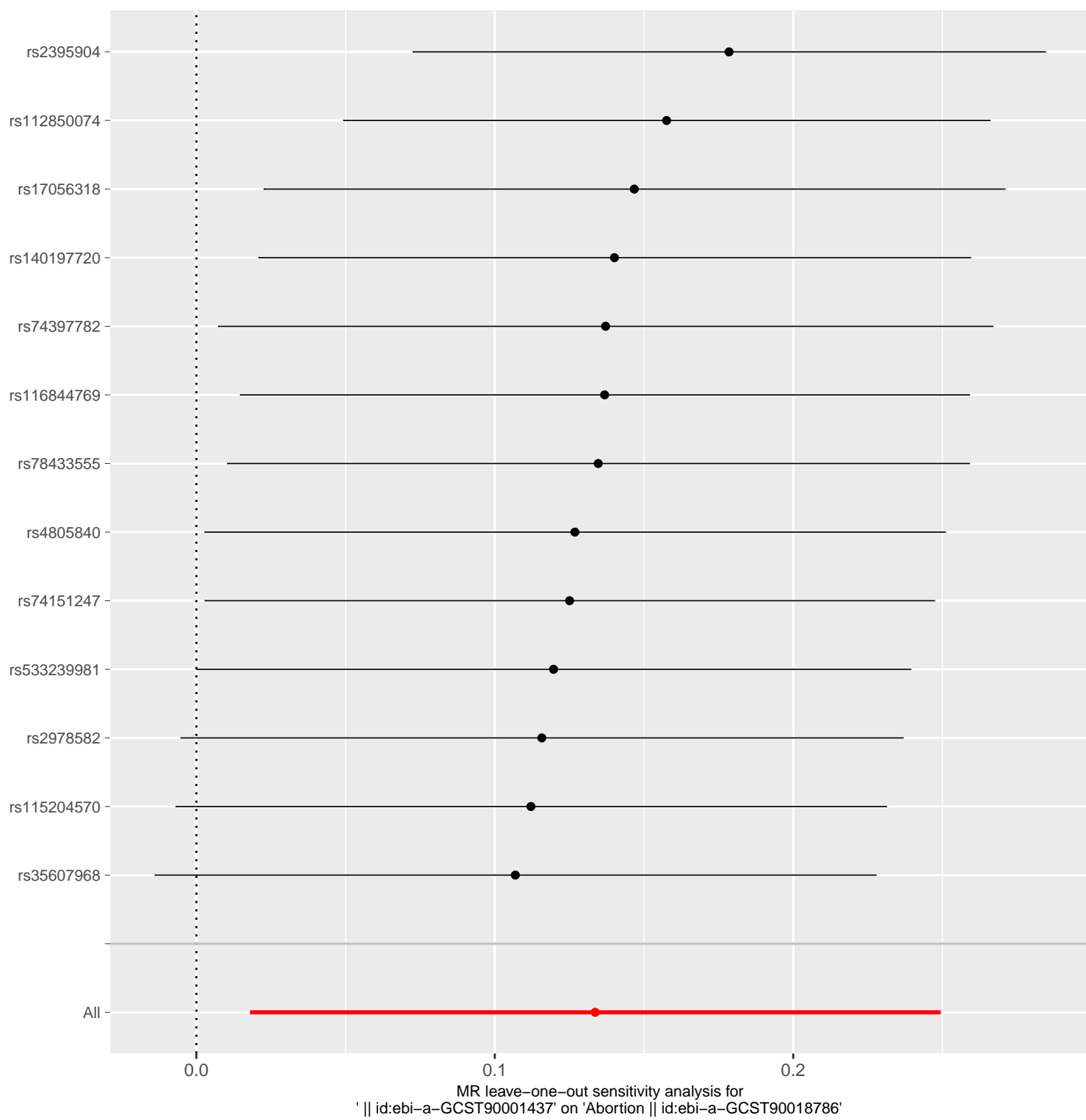

Supplement: S1 File — (ZIP) [file pone.0309088.s001.zip › S1 Fig /ebi-a-GCST90001437/sensitivity-analysis.pdf]

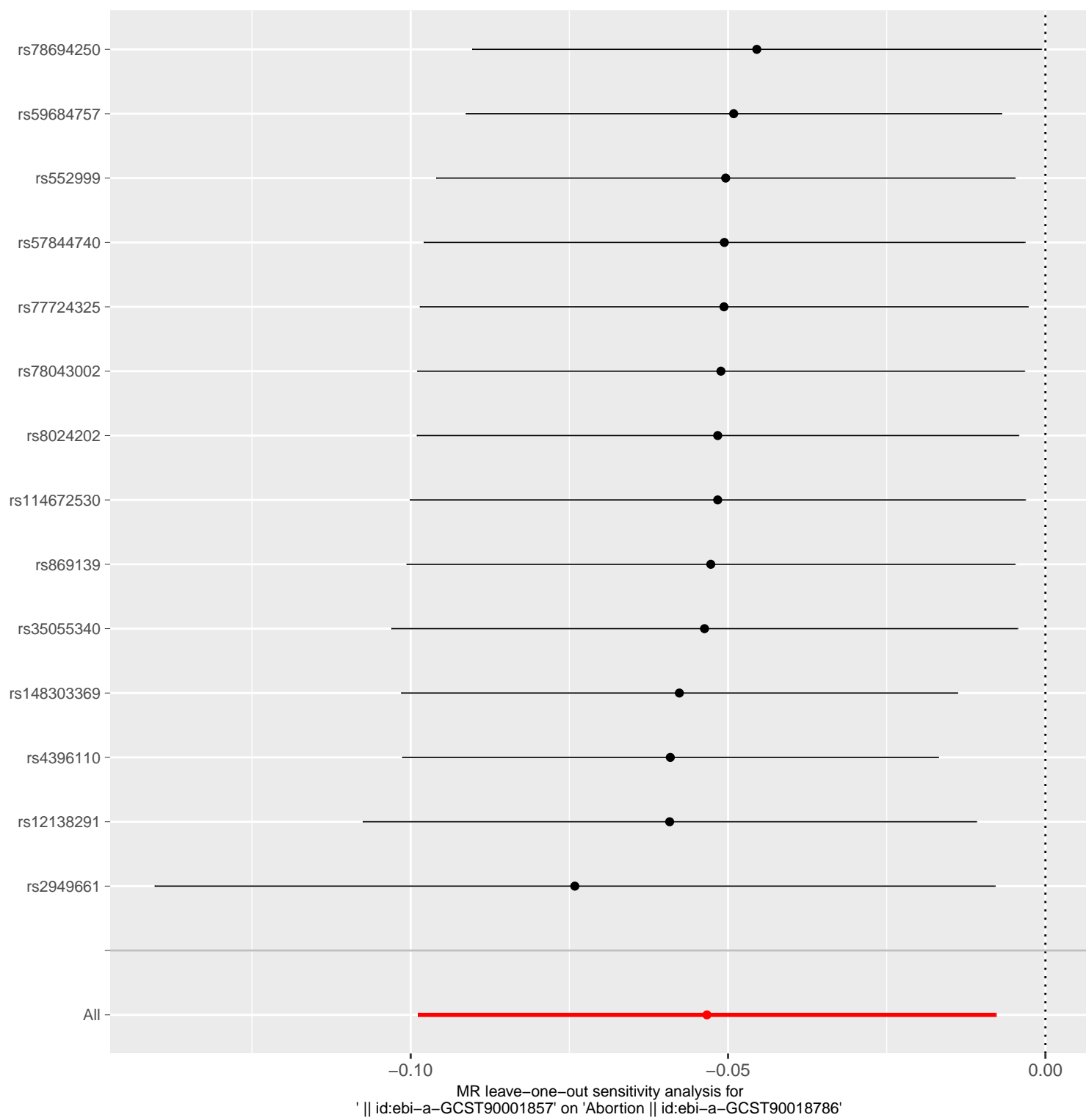

Supplement: S1 File — (ZIP) [file pone.0309088.s001.zip › S1 Fig /ebi-a-GCST90001857/sensitivity-analysis.pdf]

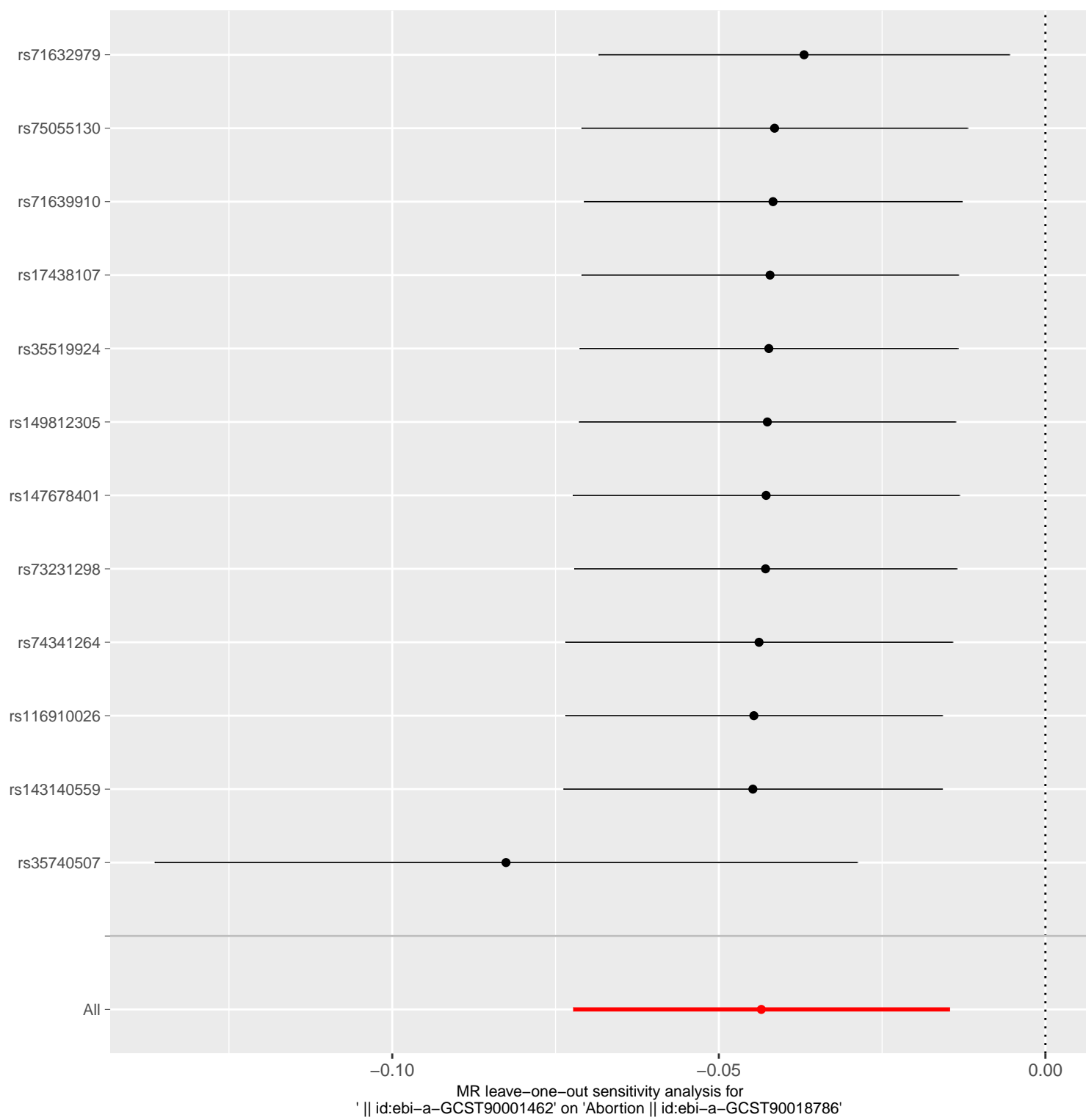

Supplement: S1 File — (ZIP) [file pone.0309088.s001.zip › S1 Fig /ebi-a-GCST90001462/sensitivity-analysis.pdf]

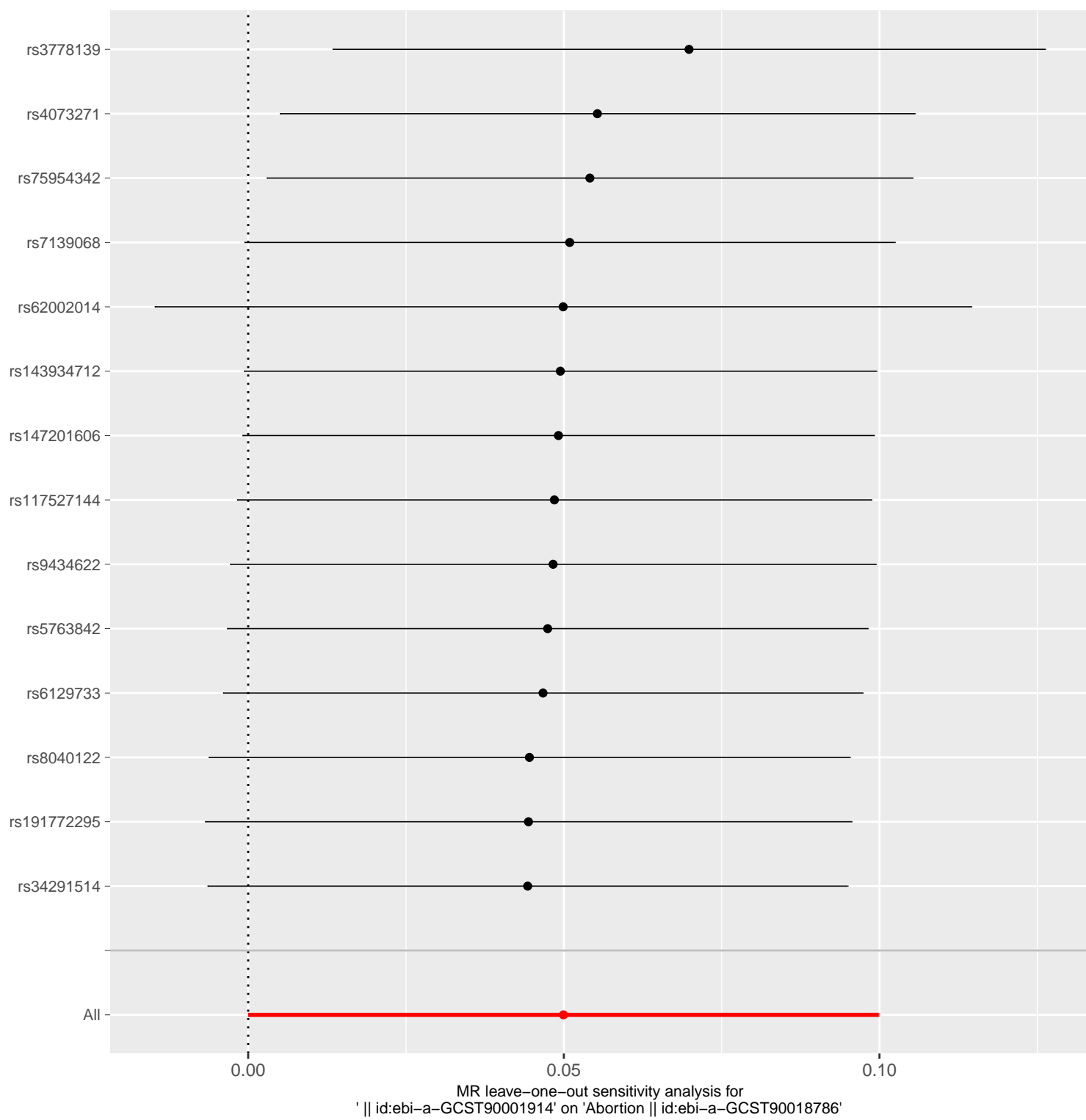

Supplement: S1 File — (ZIP) [file pone.0309088.s001.zip › S1 Fig /ebi-a-GCST90001914/sensitivity-analysis.pdf]

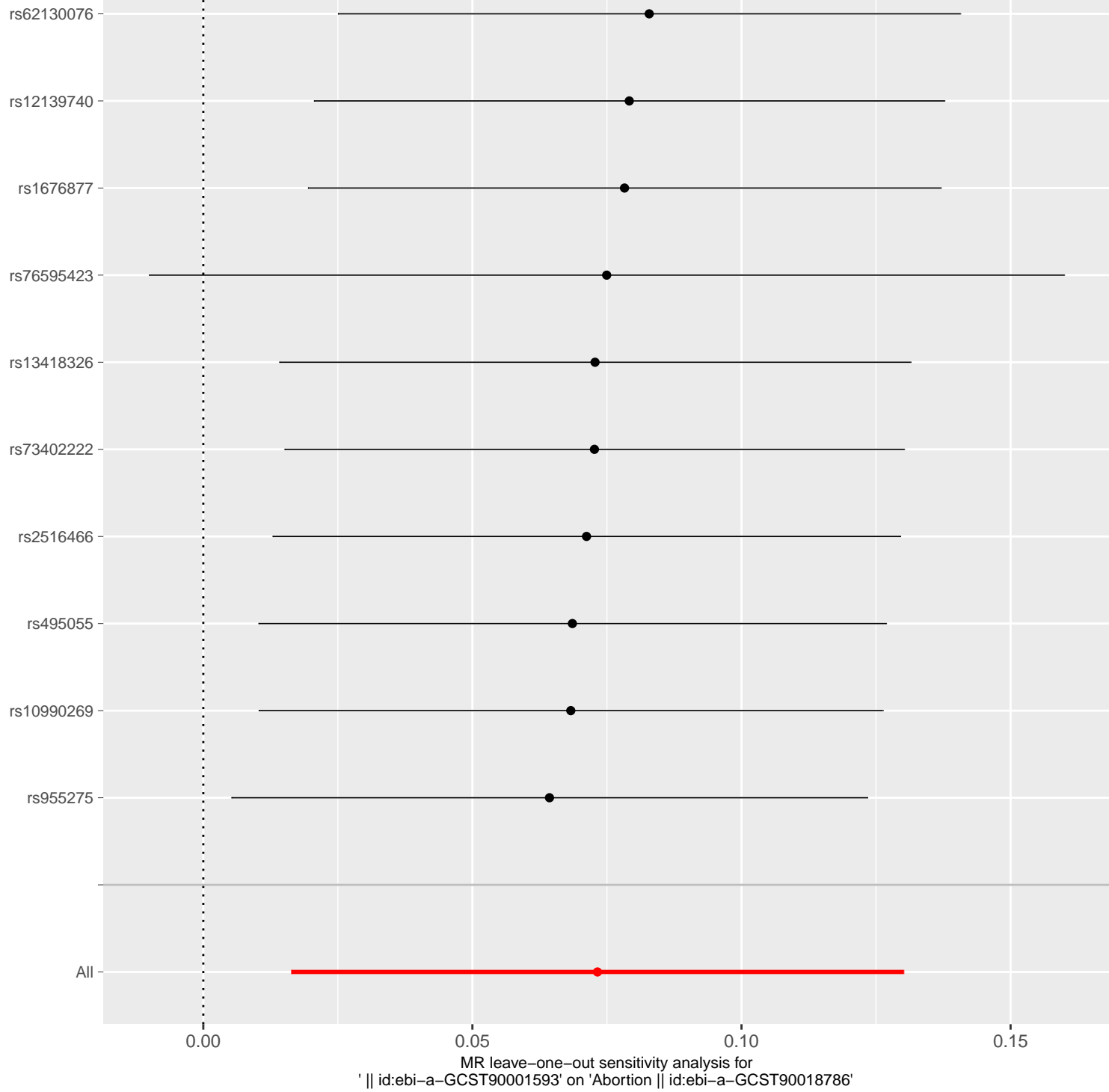

Supplement: S1 File — (ZIP) [file pone.0309088.s001.zip › S1 Fig /ebi-a-GCST90001593/sensitivity-analysis.pdf]

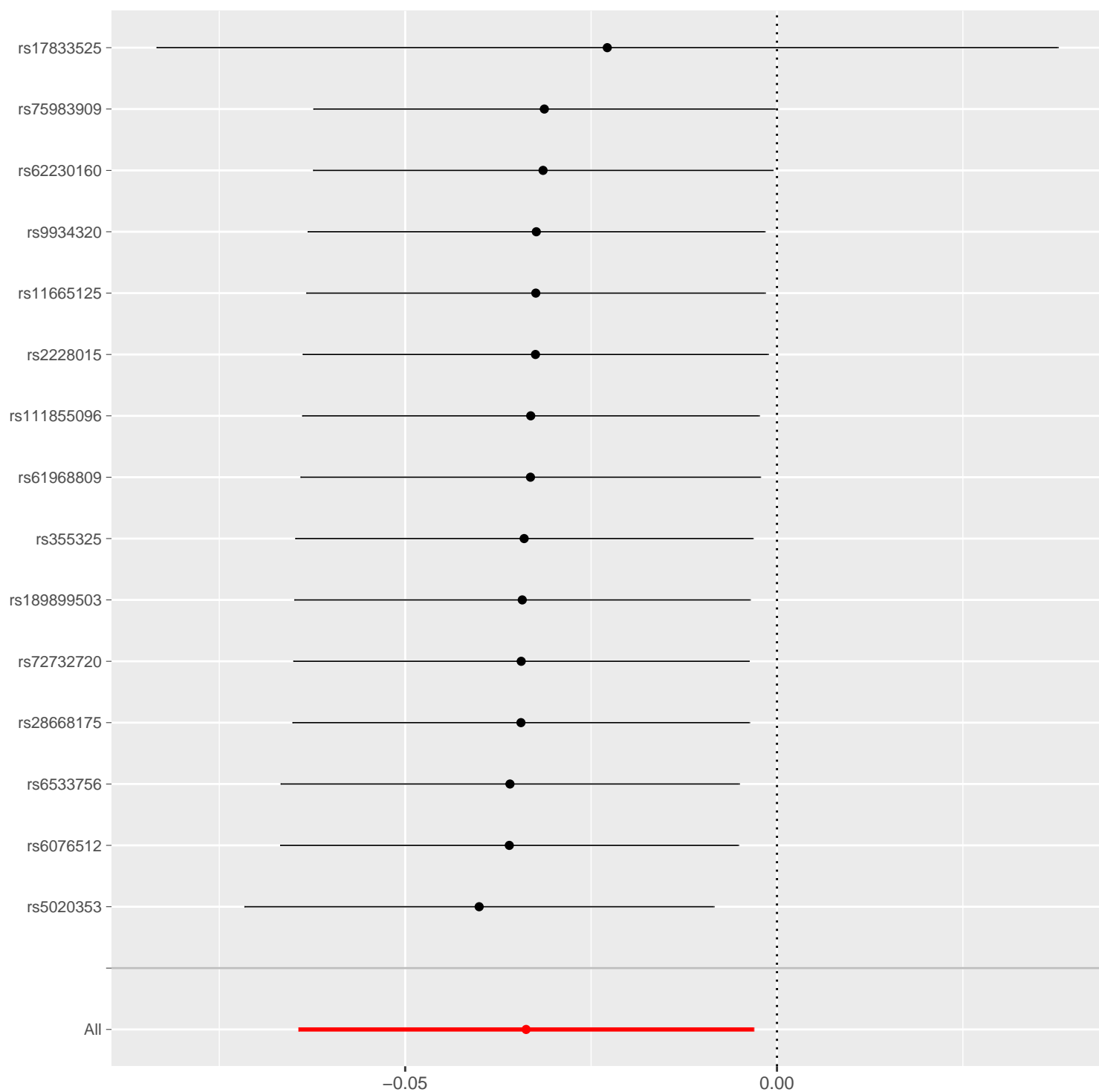

MR leave-one-out sensitivity analysis for  
' || id:ebi-a-GCST90001907' on 'Abortion || id:ebi-a-GCST90018786'

Supplement: S1 File — (ZIP) [file pone.0309088.s001.zip › S1 Fig /ebi-a-GCST90001907/sensitivity-analysis.pdf]

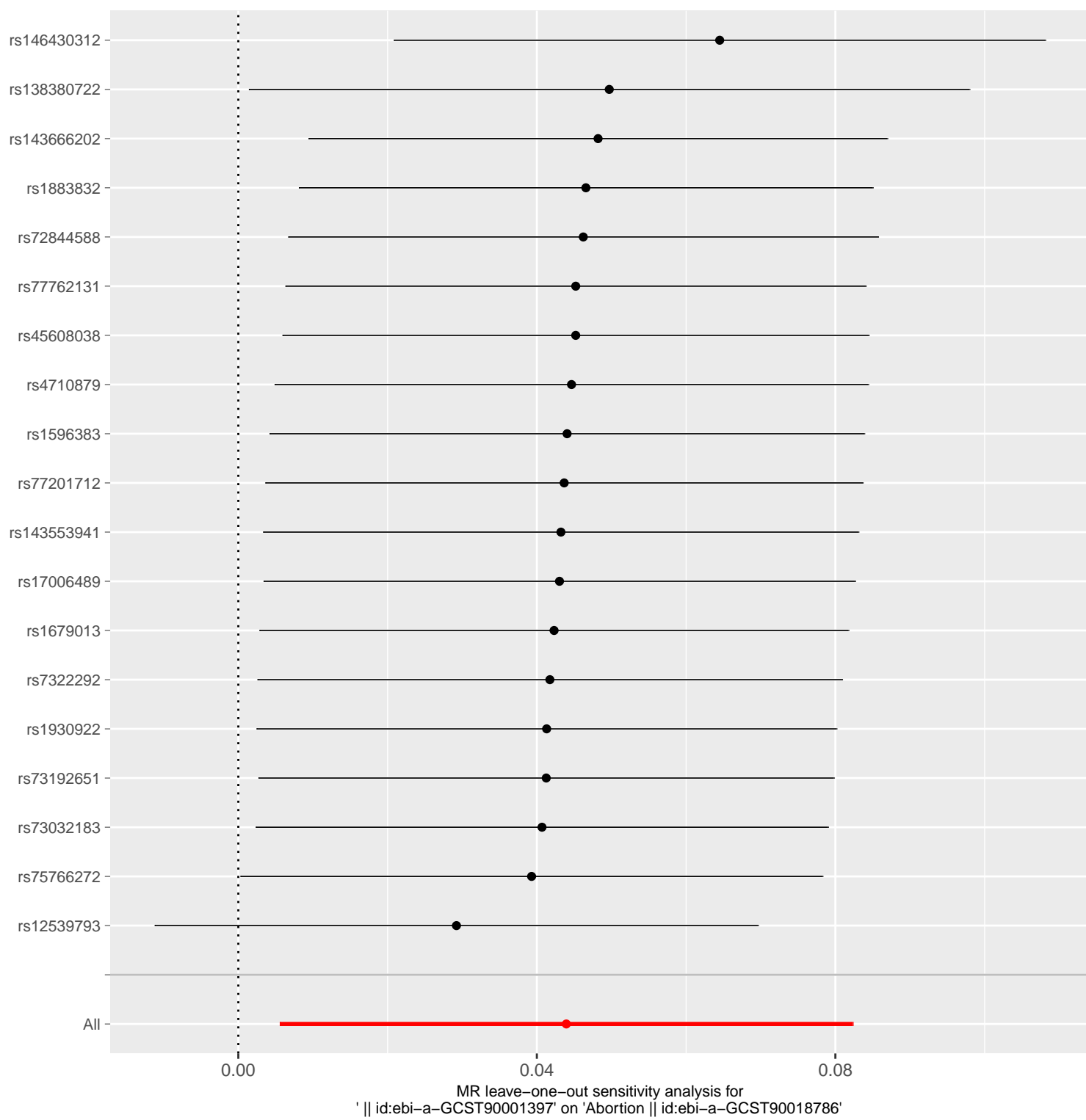

Supplement: S1 File — (ZIP) [file pone.0309088.s001.zip › S1 Fig /ebi-a-GCST90001397/sensitivity-analysis.pdf]

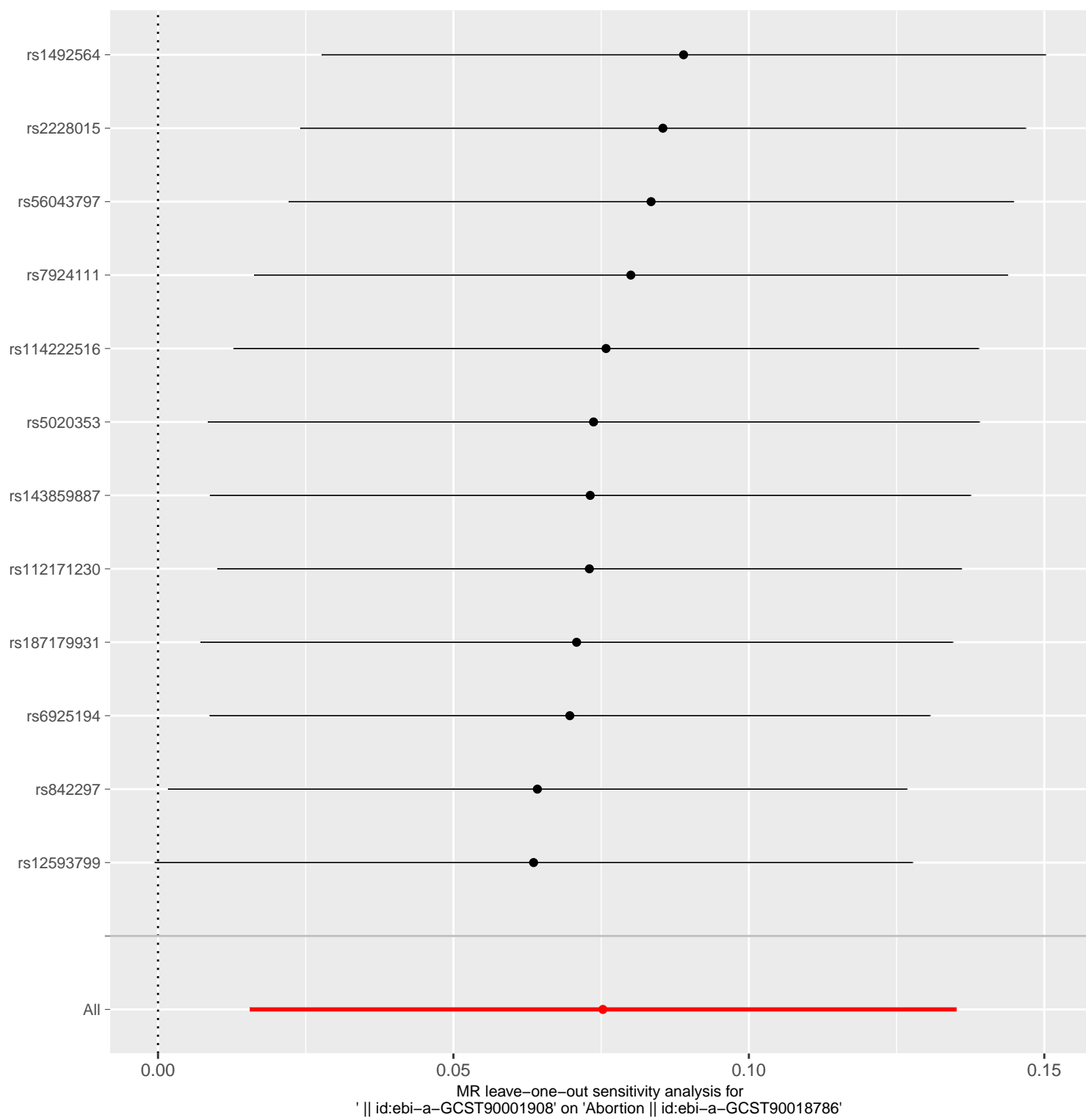

Supplement: S1 File — (ZIP) [file pone.0309088.s001.zip › S1 Fig /ebi-a-GCST90001908/sensitivity-analysis.pdf]

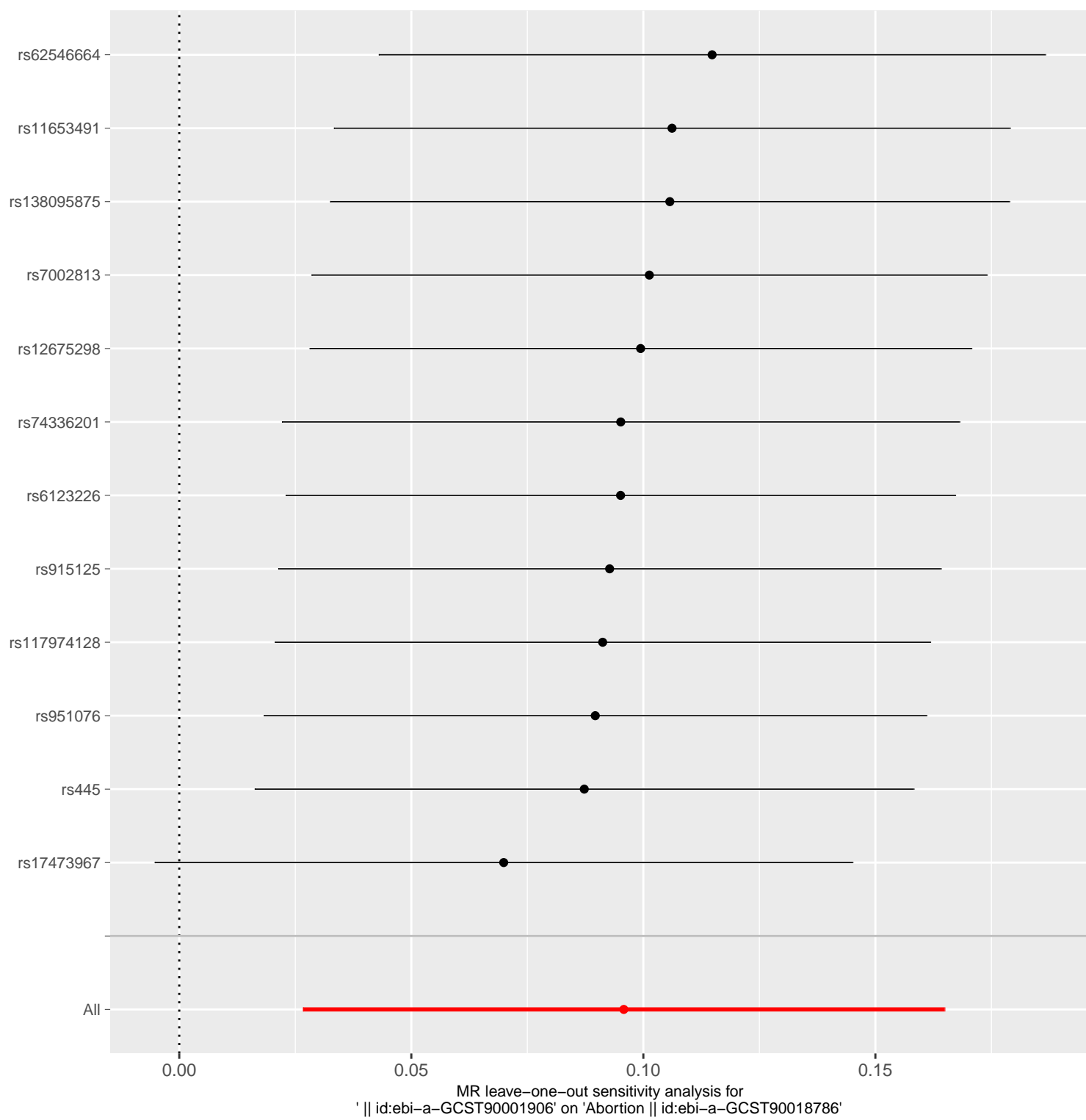

Supplement: S1 File — (ZIP) [file pone.0309088.s001.zip › S1 Fig /ebi-a-GCST90001906/sensitivity-analysis.pdf]

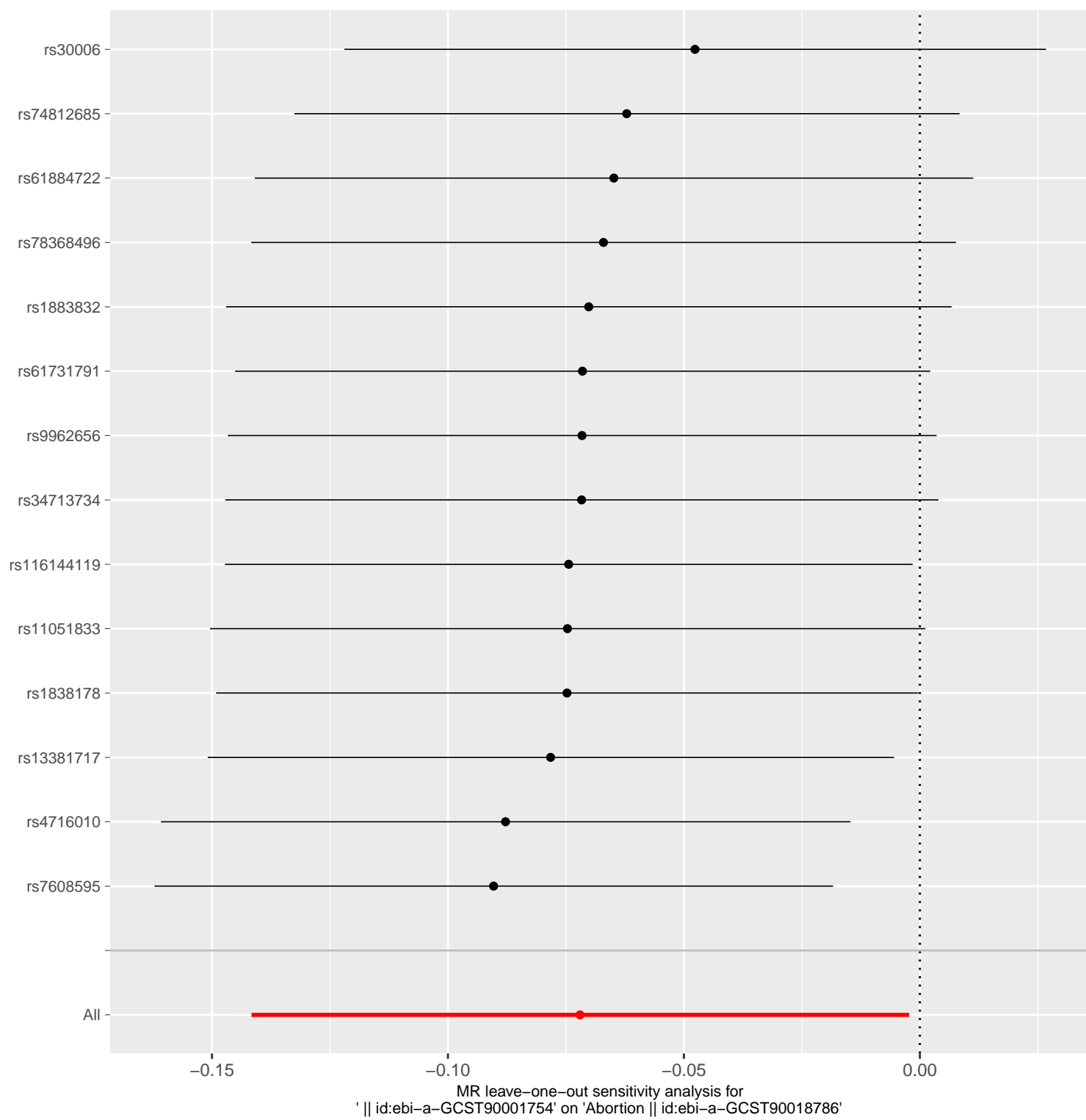

Supplement: S1 File — (ZIP) [file pone.0309088.s001.zip › S1 Fig /ebi-a-GCST90001754/sensitivity-analysis.pdf]

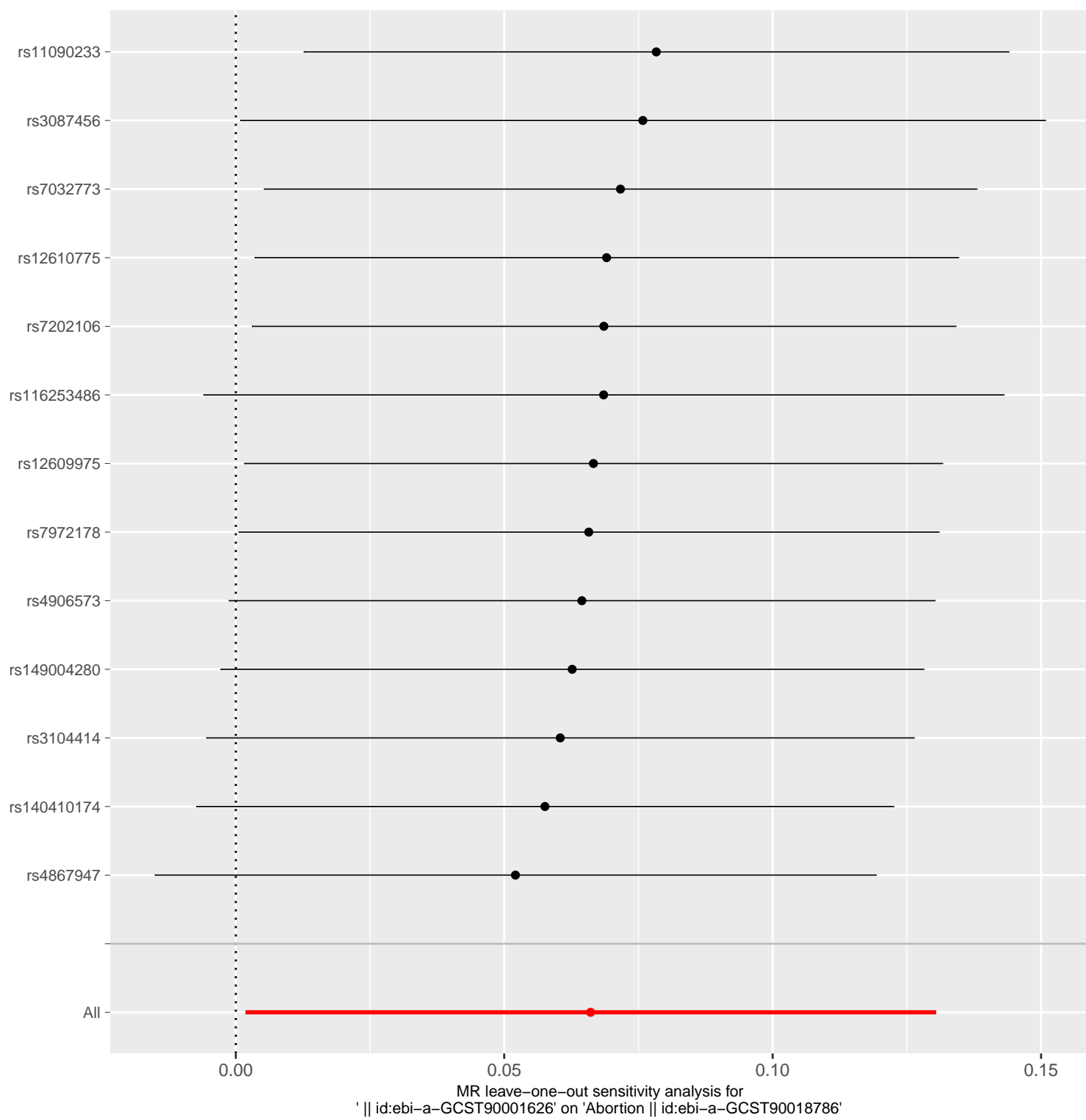

Supplement: S1 File — (ZIP) [file pone.0309088.s001.zip › S1 Fig /ebi-a-GCST90001626/sensitivity-analysis.pdf]

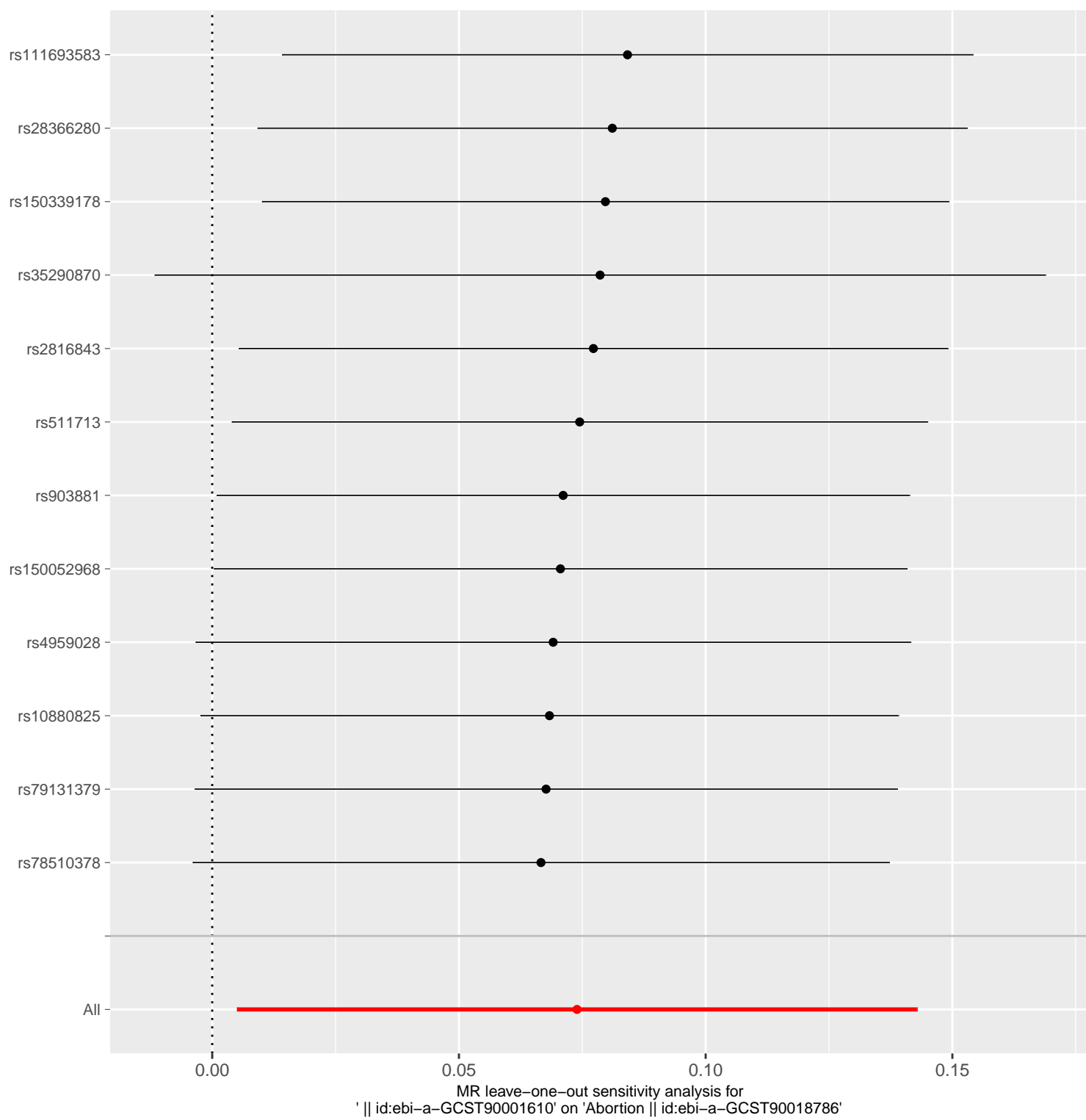

Supplement: S1 File — (ZIP) [file pone.0309088.s001.zip › S1 Fig /ebi-a-GCST90001610/sensitivity-analysis.pdf]

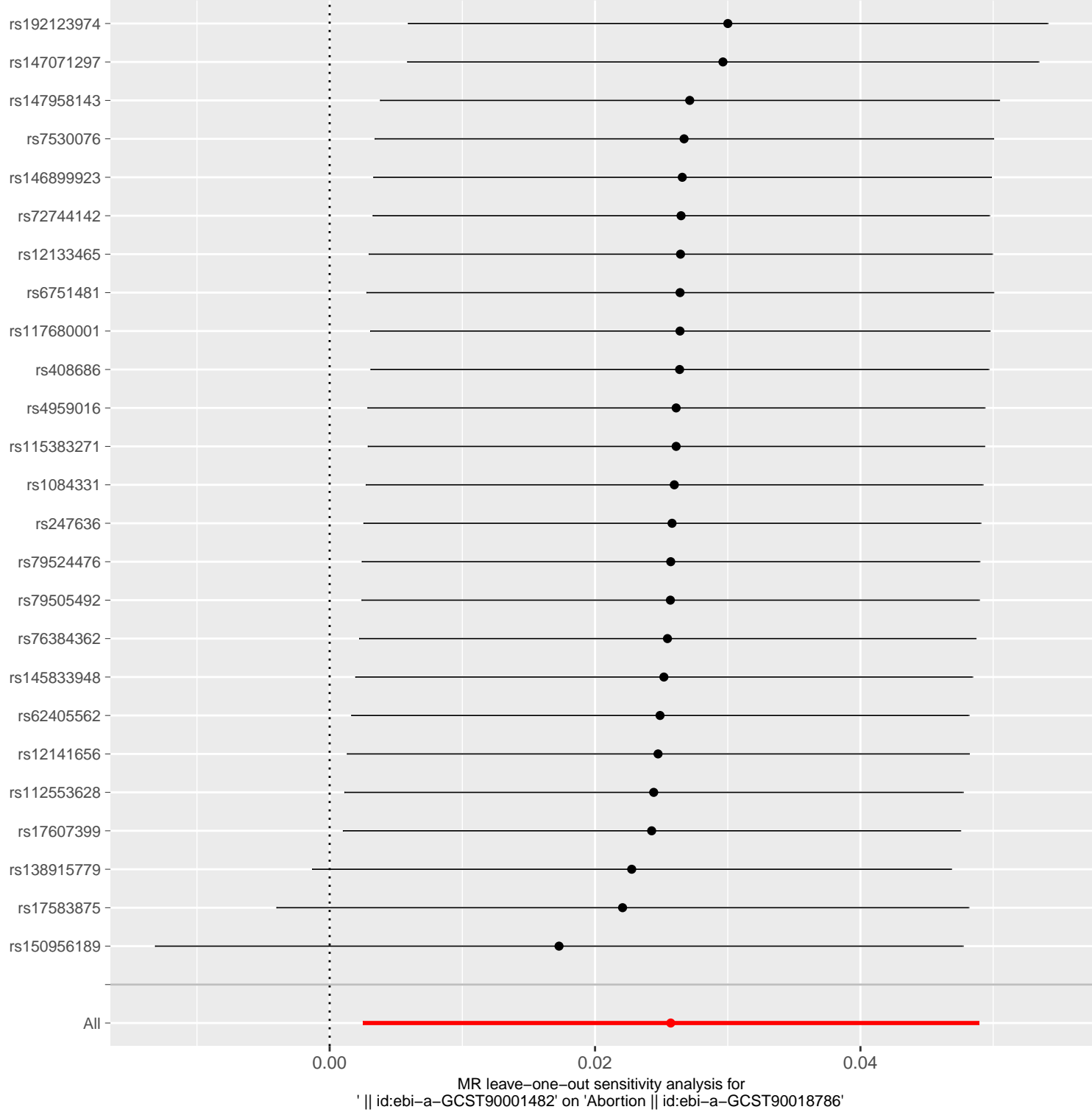

Supplement: S1 File — (ZIP) [file pone.0309088.s001.zip › S1 Fig /ebi-a-GCST90001482/sensitivity-analysis.pdf]

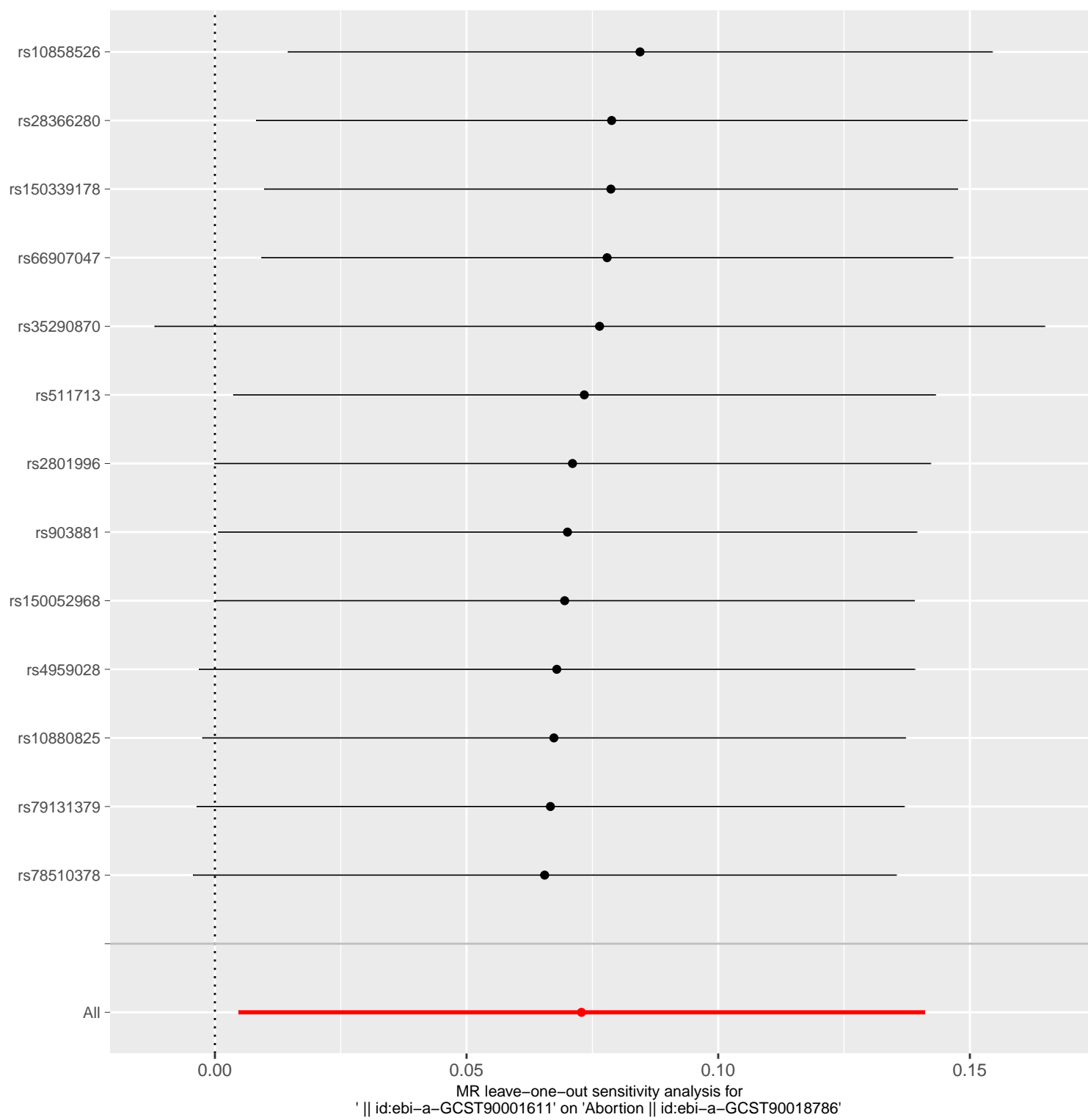

Supplement: S1 File — (ZIP) [file pone.0309088.s001.zip › S1 Fig /ebi-a-GCST90001611/sensitivity-analysis.pdf]

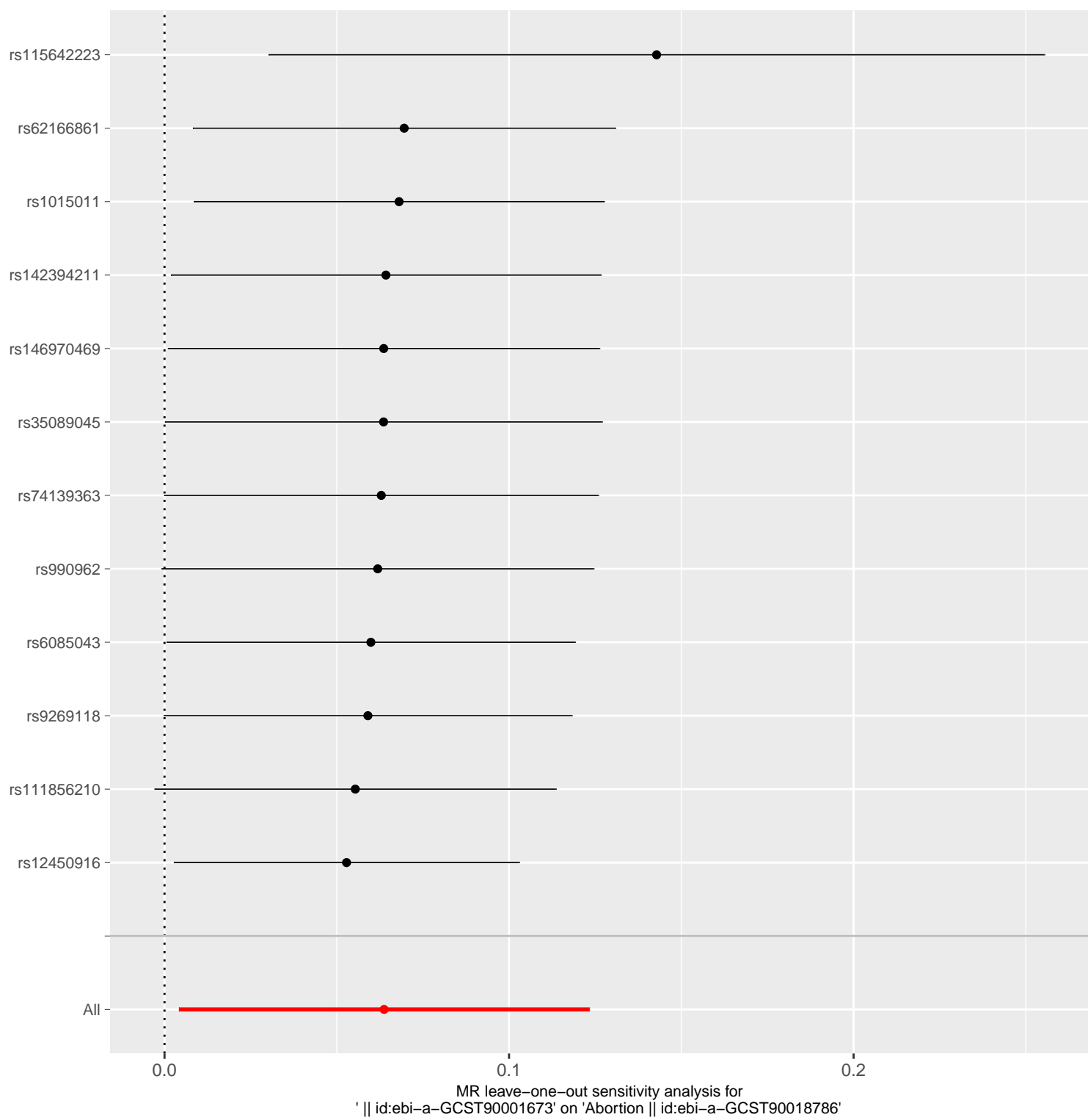

Supplement: S1 File — (ZIP) [file pone.0309088.s001.zip › S1 Fig /ebi-a-GCST90001673/sensitivity-analysis.pdf]

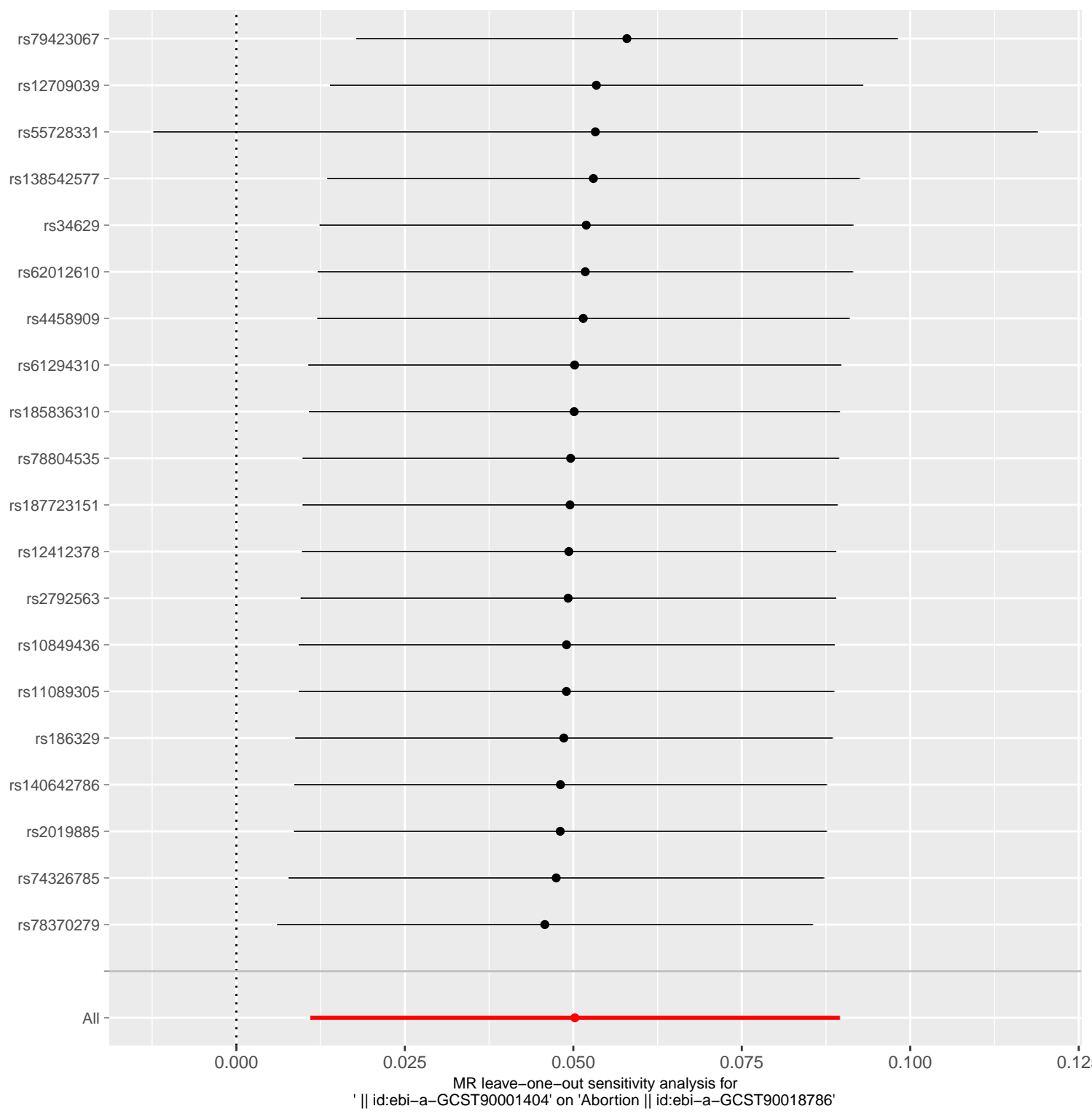

Supplement: S1 File — (ZIP) [file pone.0309088.s001.zip › S1 Fig /ebi-a-GCST90001404/sensitivity-analysis.pdf]

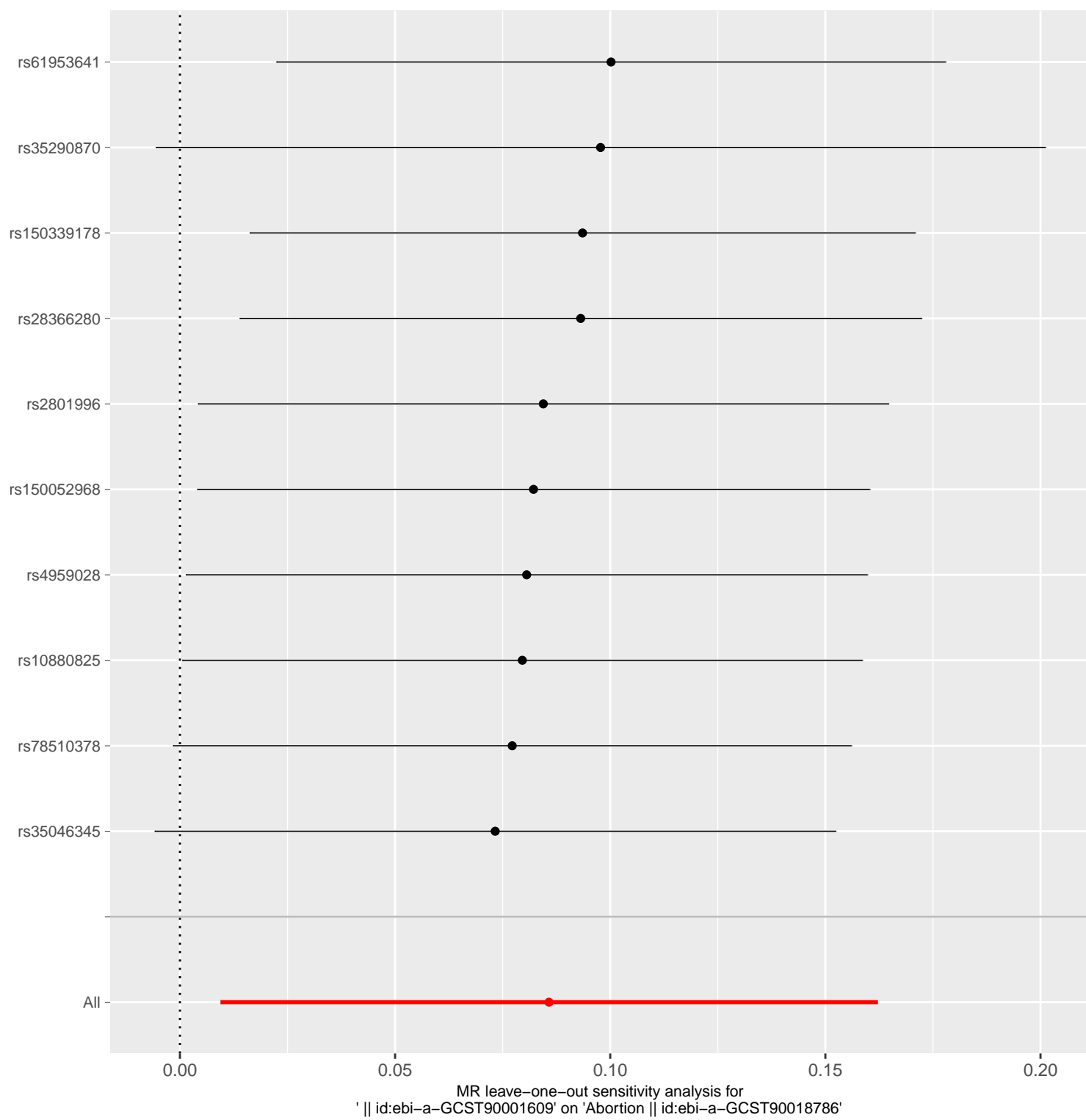

Supplement: S1 File — (ZIP) [file pone.0309088.s001.zip › S1 Fig /ebi-a-GCST90001609/sensitivity-analysis.pdf]

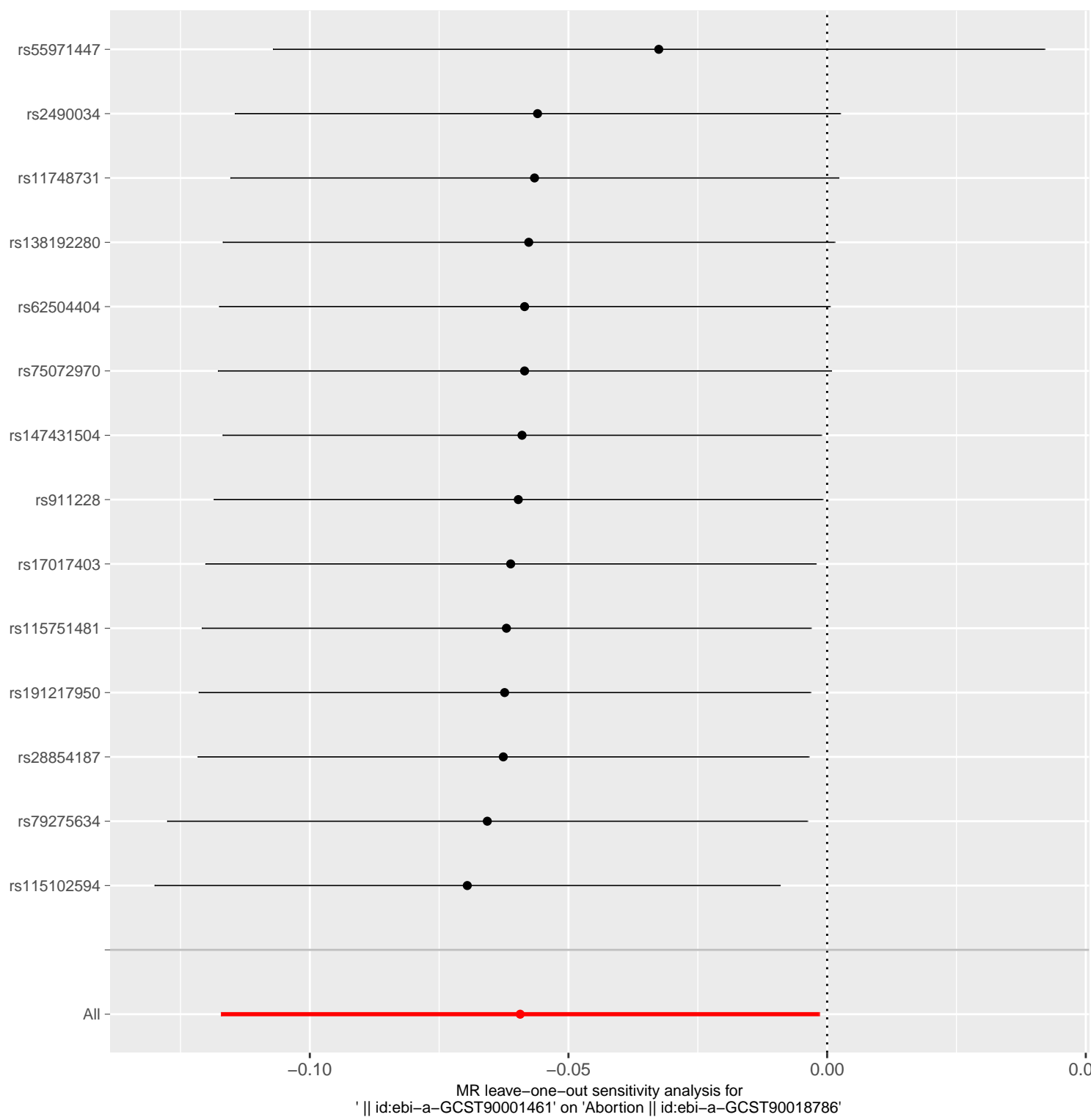

Supplement: S1 File — (ZIP) [file pone.0309088.s001.zip › S1 Fig /ebi-a-GCST90001461/sensitivity-analysis.pdf]

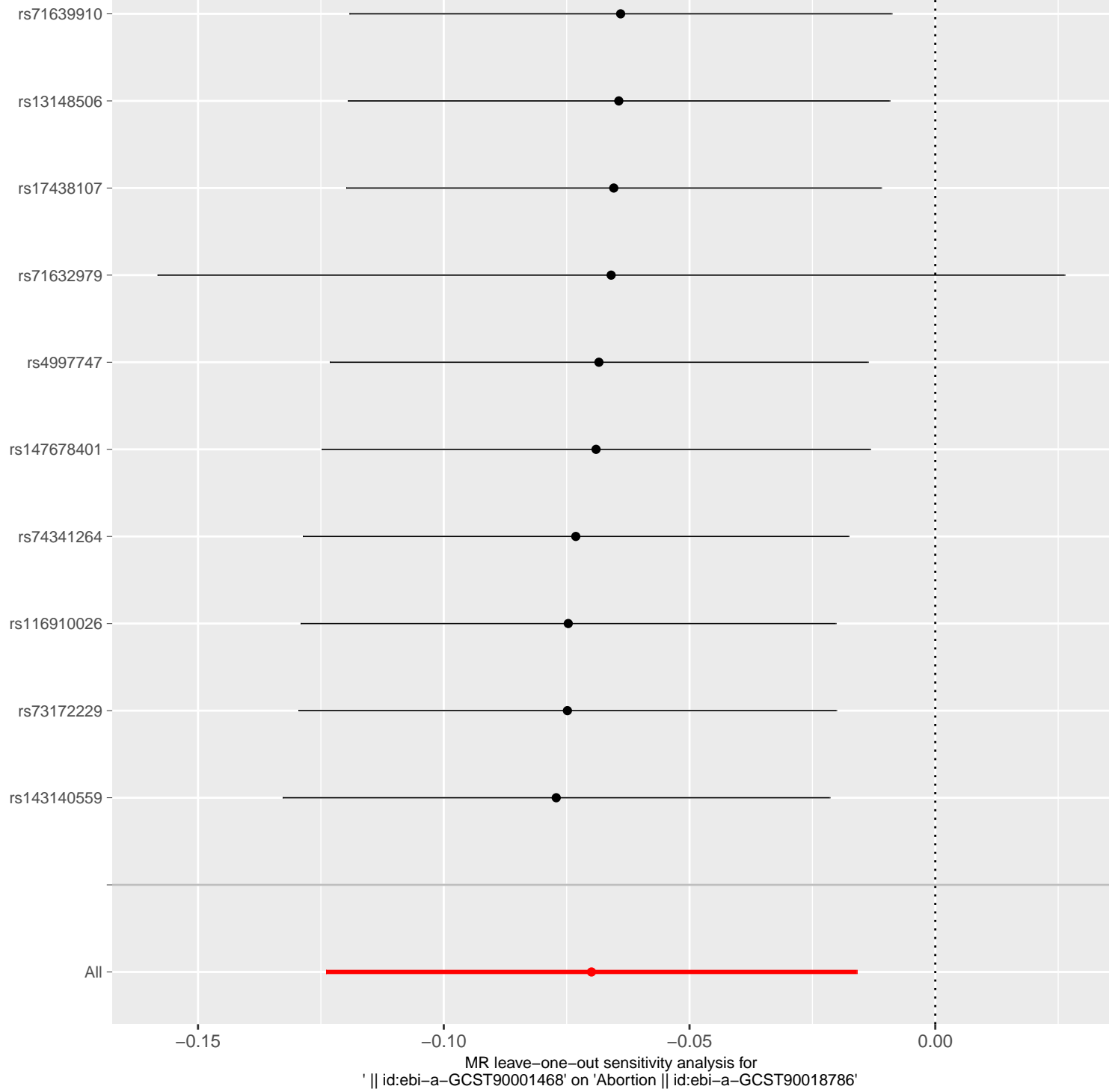

Supplement: S1 File — (ZIP) [file pone.0309088.s001.zip › S1 Fig /ebi-a-GCST90001468/sensitivity-analysis.pdf]

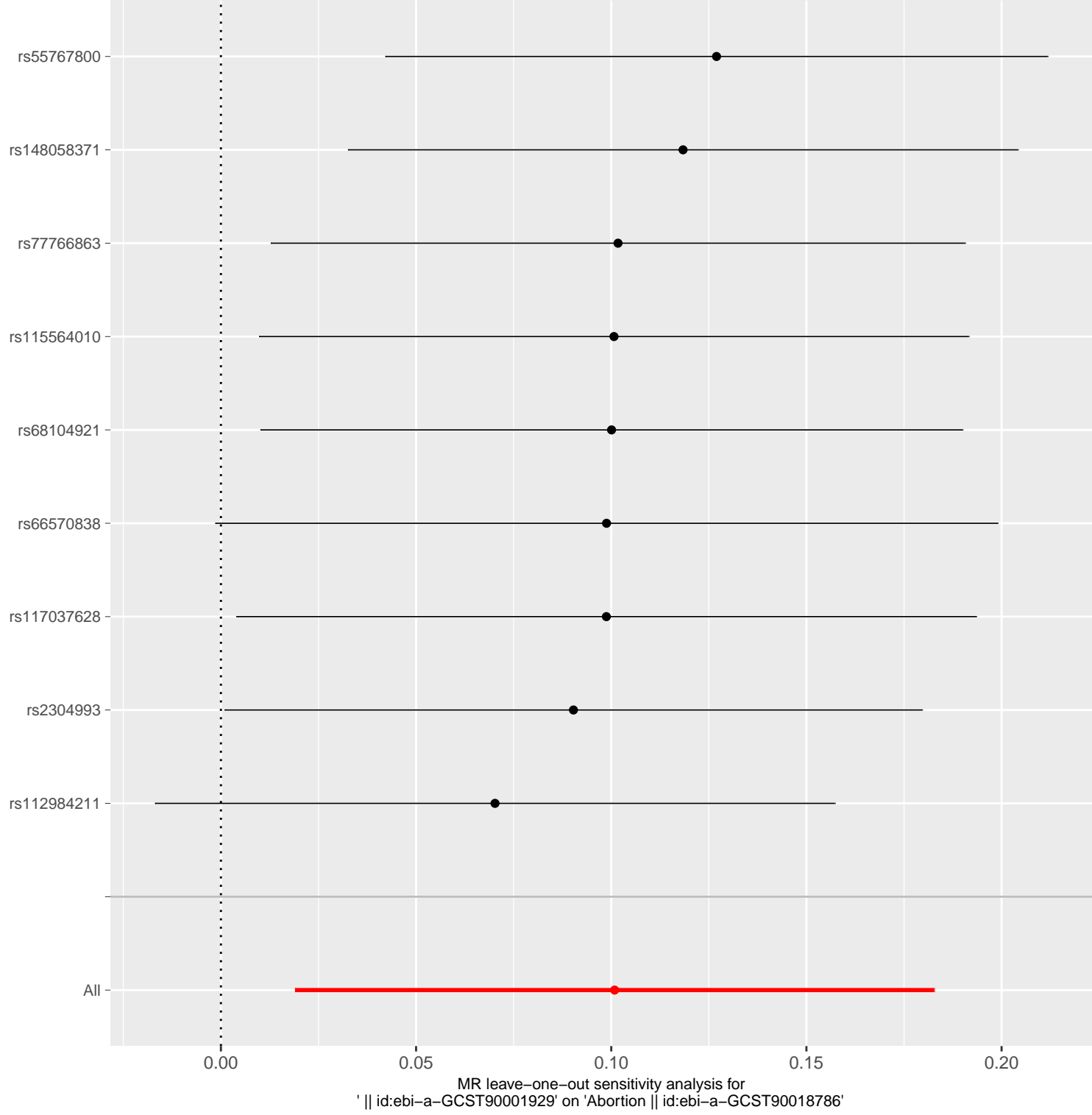

Supplement: S1 File — (ZIP) [file pone.0309088.s001.zip › S1 Fig /ebi-a-GCST90001929/sensitivity-analysis.pdf]

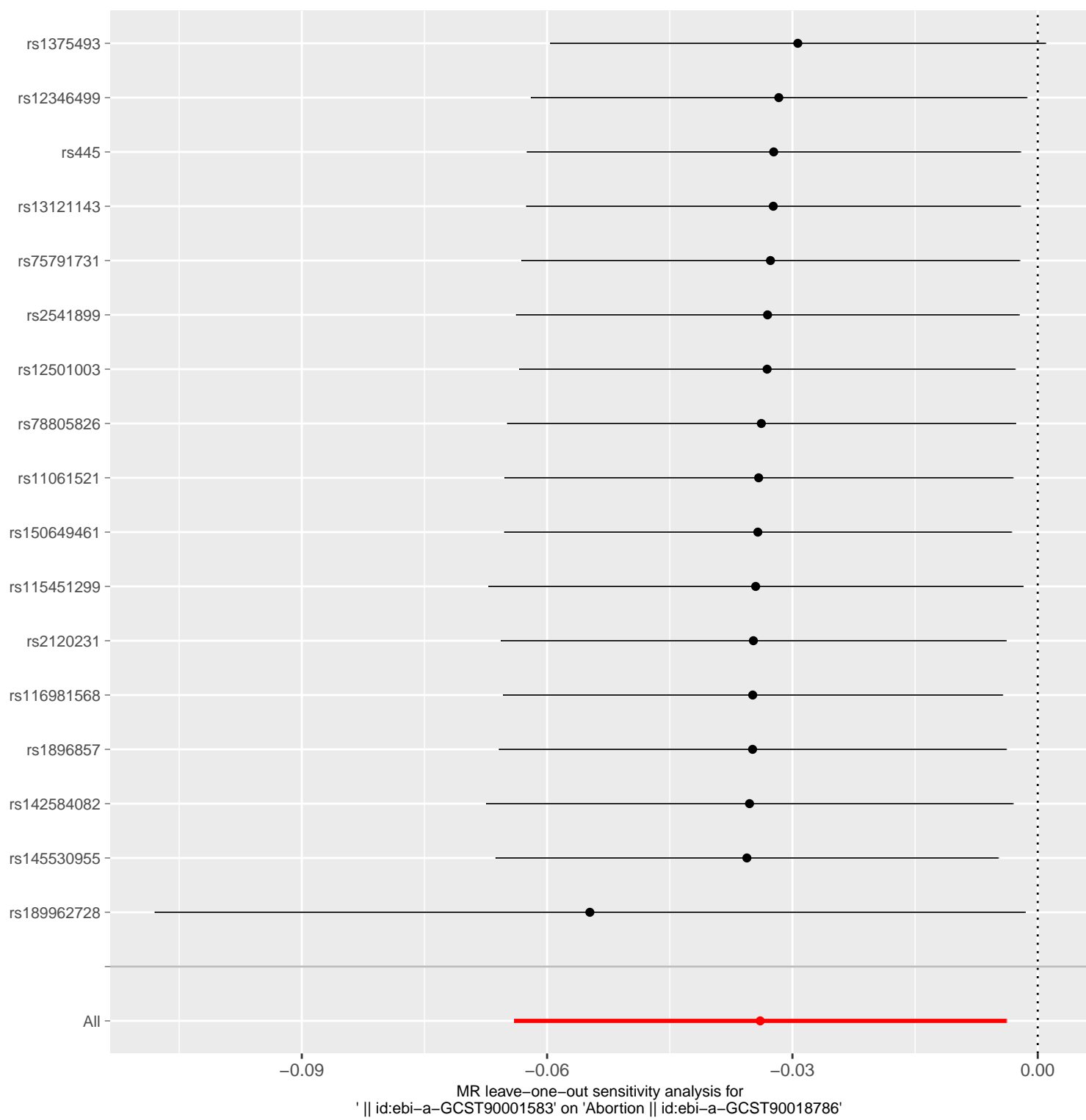

Supplement: S1 File — (ZIP) [file pone.0309088.s001.zip › S1 Fig /ebi-a-GCST90001583/sensitivity-analysis.pdf]

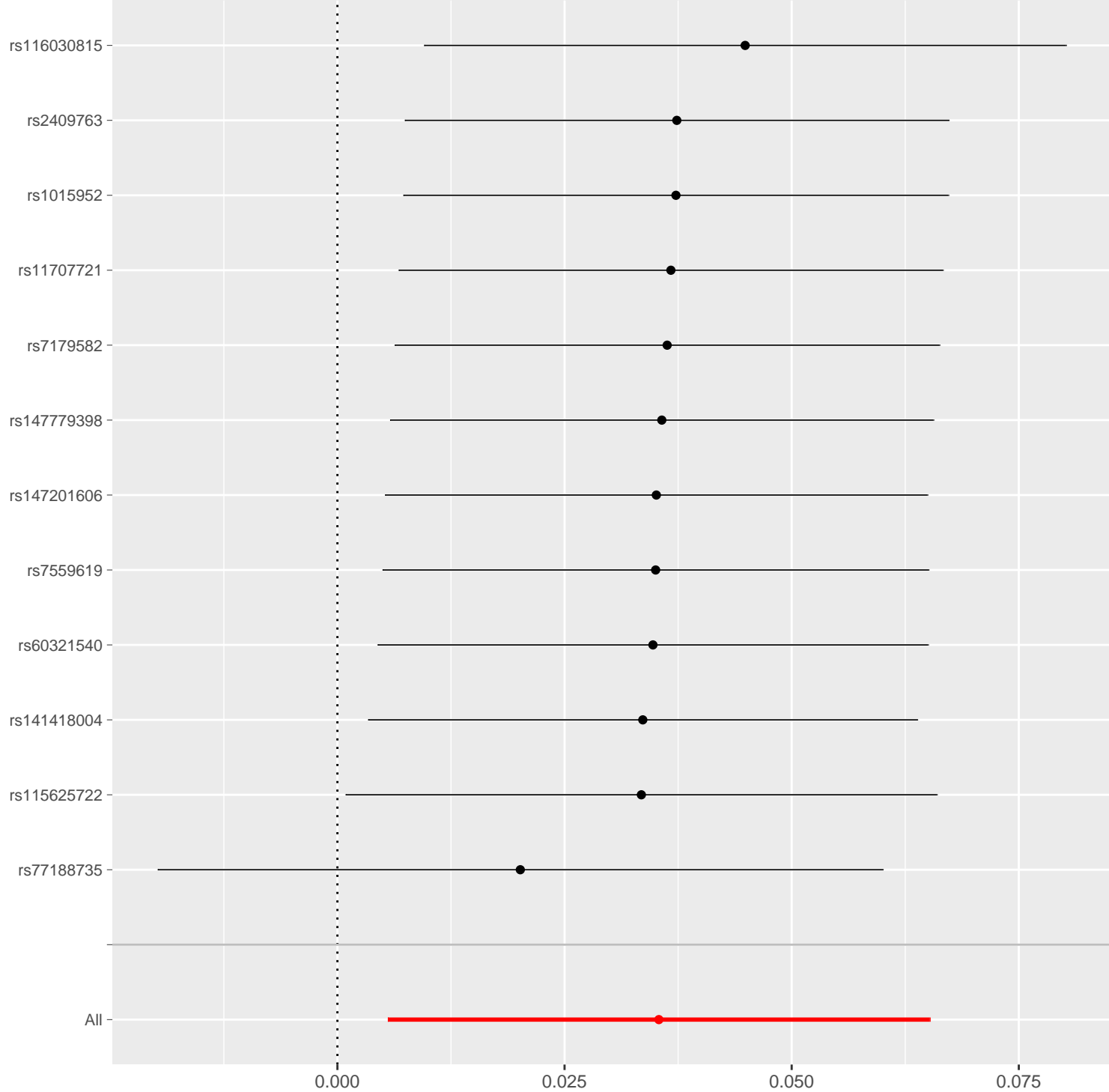

Supplement: S1 File — (ZIP) [file pone.0309088.s001.zip › S1 Fig /ebi-a-GCST90001917/sensitivity-analysis.pdf]

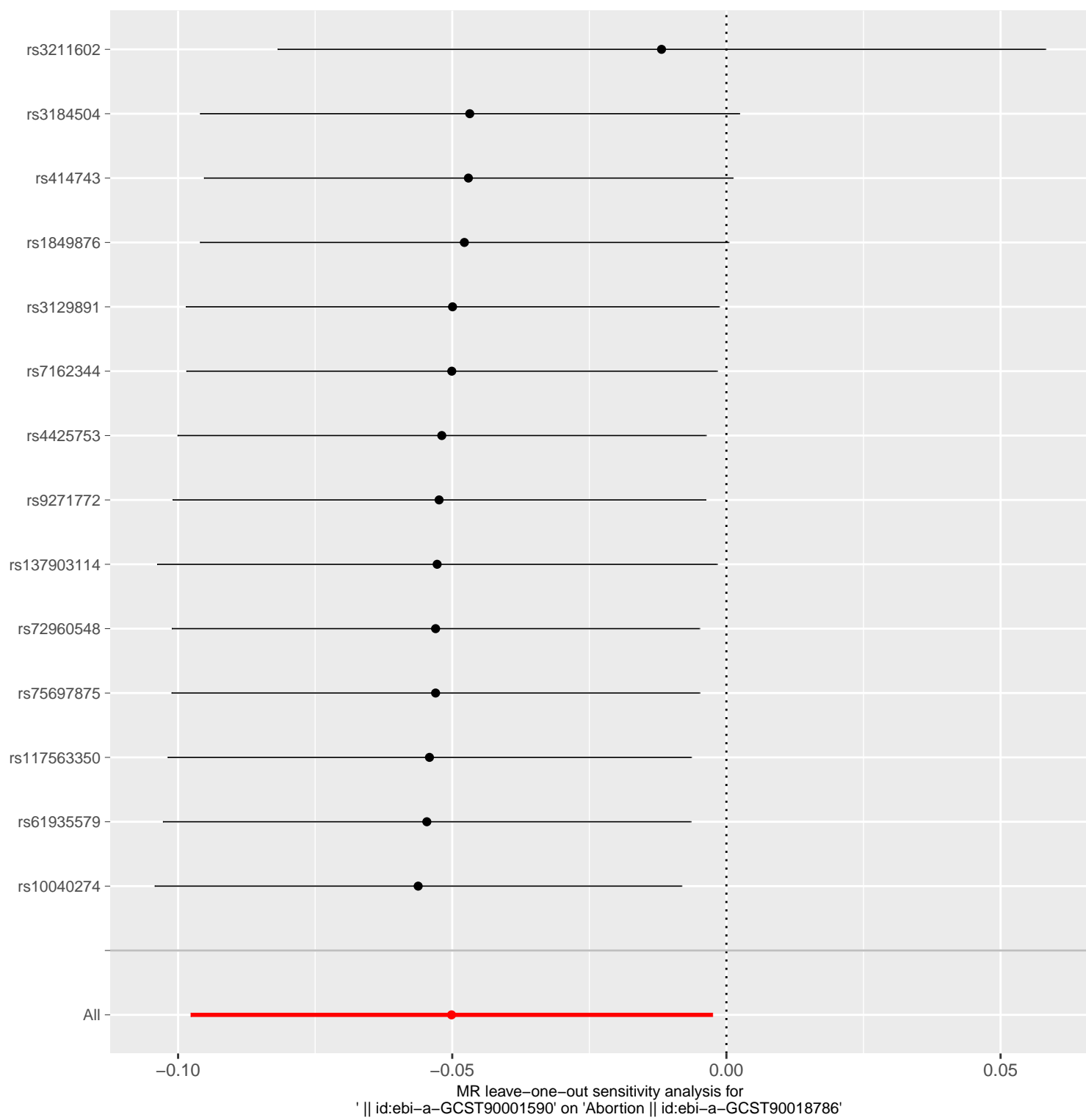

Supplement: S1 File — (ZIP) [file pone.0309088.s001.zip › S1 Fig /ebi-a-GCST90001590/sensitivity-analysis.pdf]

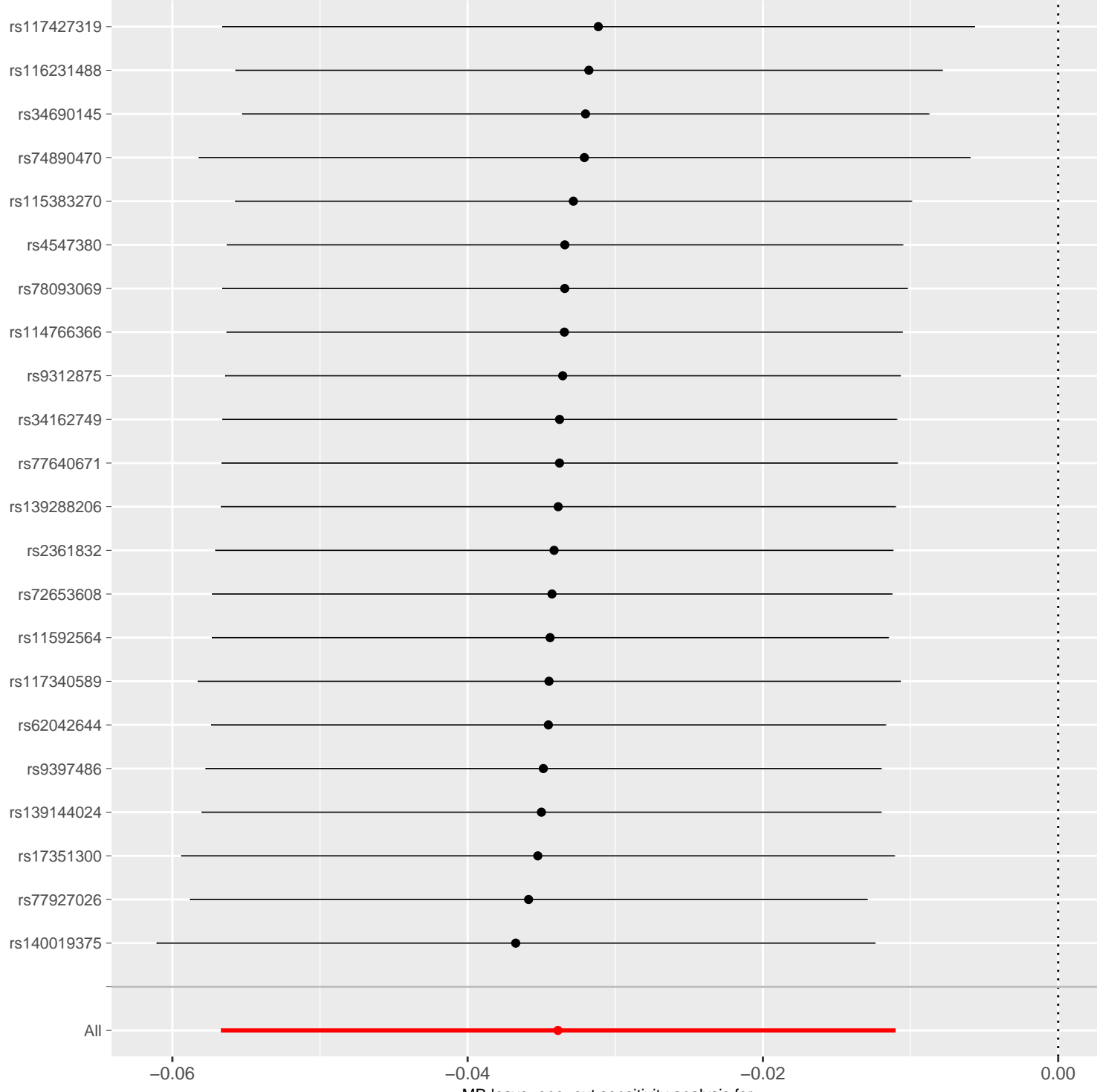

Supplement: S1 File — (ZIP) [file pone.0309088.s001.zip › S1 Fig /ebi-a-GCST90001992/sensitivity-analysis.pdf]

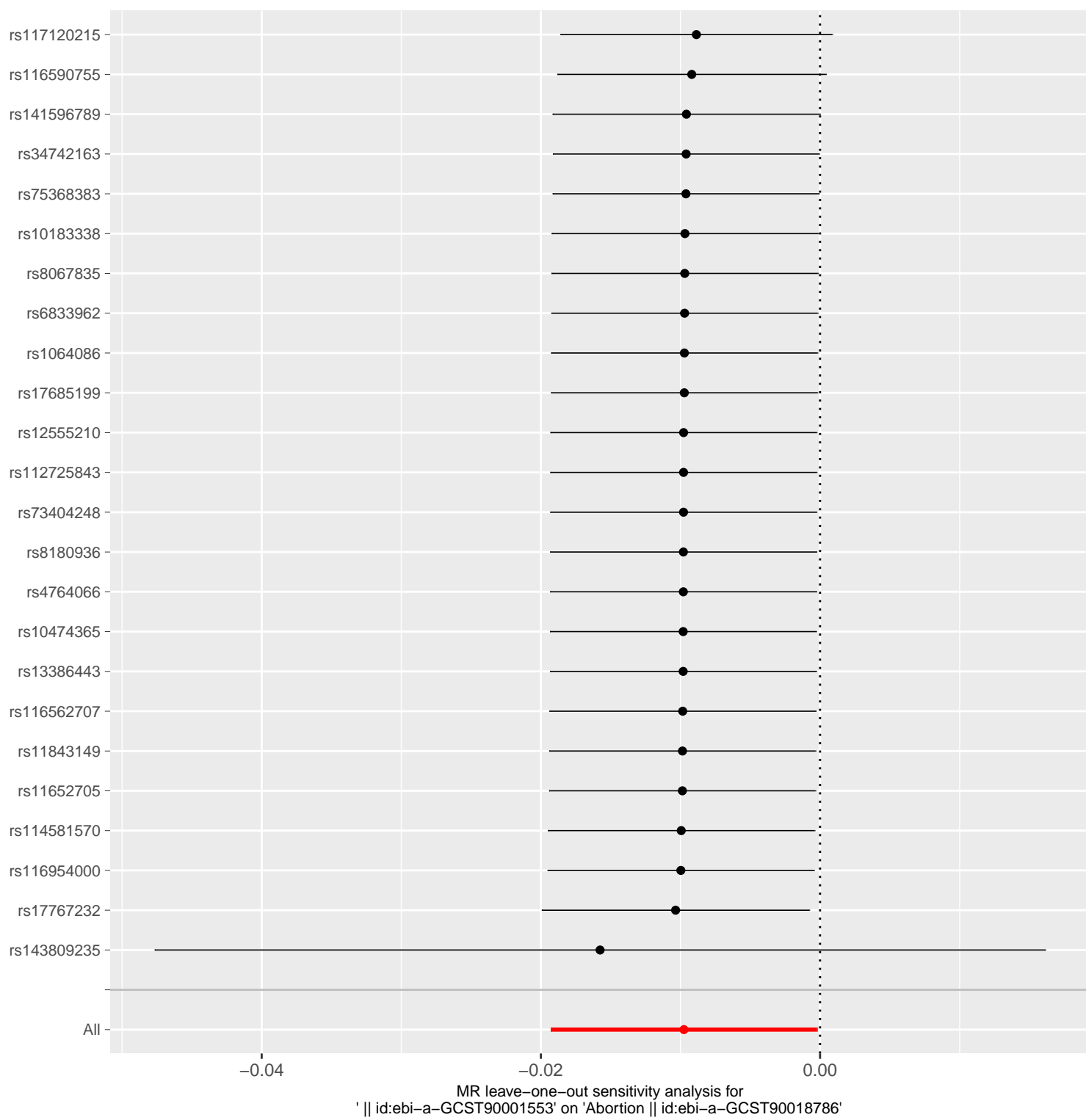

Supplement: S1 File — (ZIP) [file pone.0309088.s001.zip › S1 Fig /ebi-a-GCST90001553/sensitivity-analysis.pdf]

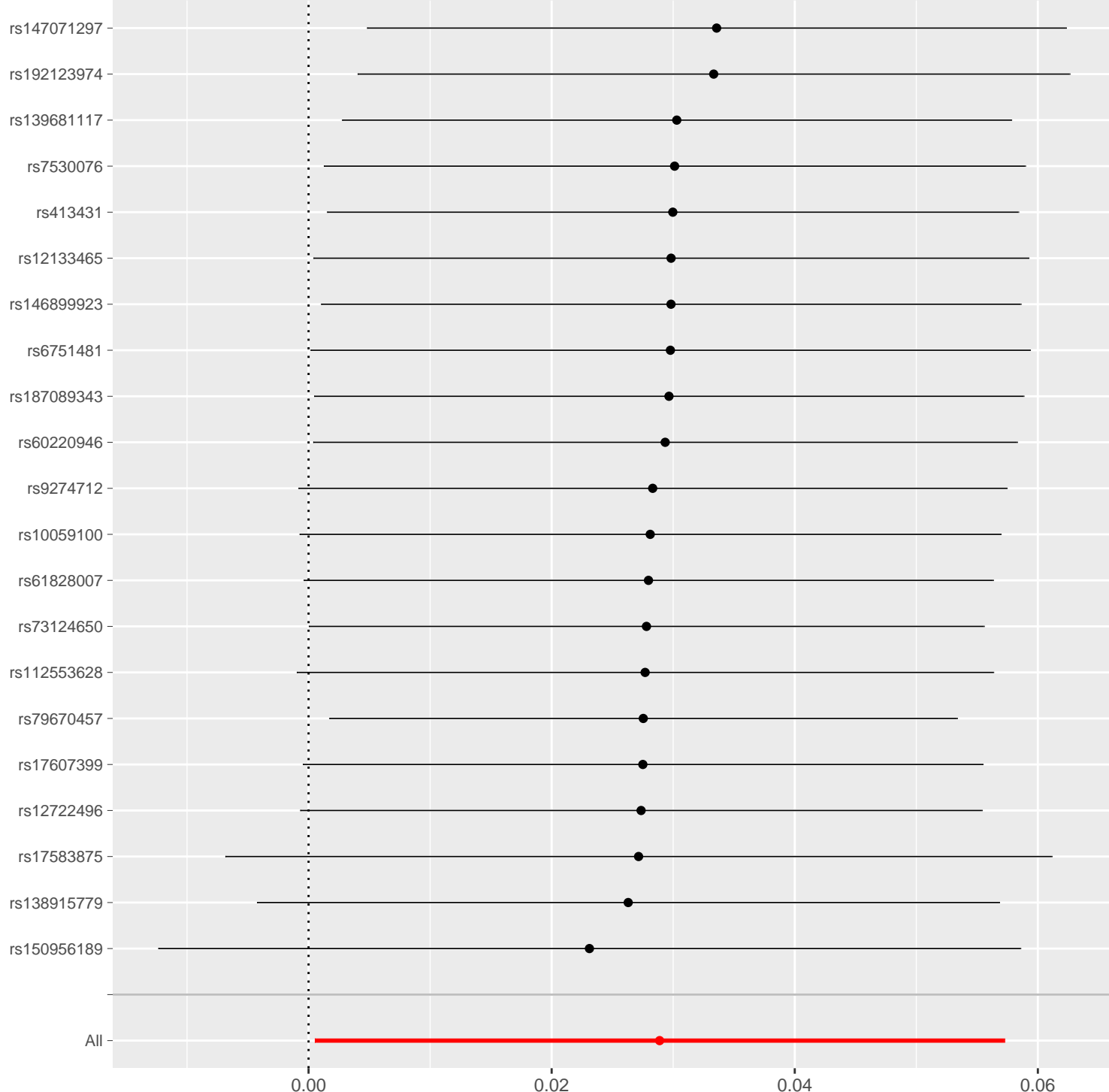

MR leave-one-out sensitivity analysis for  
' || id:ebi-a-GCST90001481' on 'Abortion || id:ebi-a-GCST90018786'

Supplement: S1 File — (ZIP) [file pone.0309088.s001.zip › S1 Fig /ebi-a-GCST90001481/sensitivity-analysis.pdf]

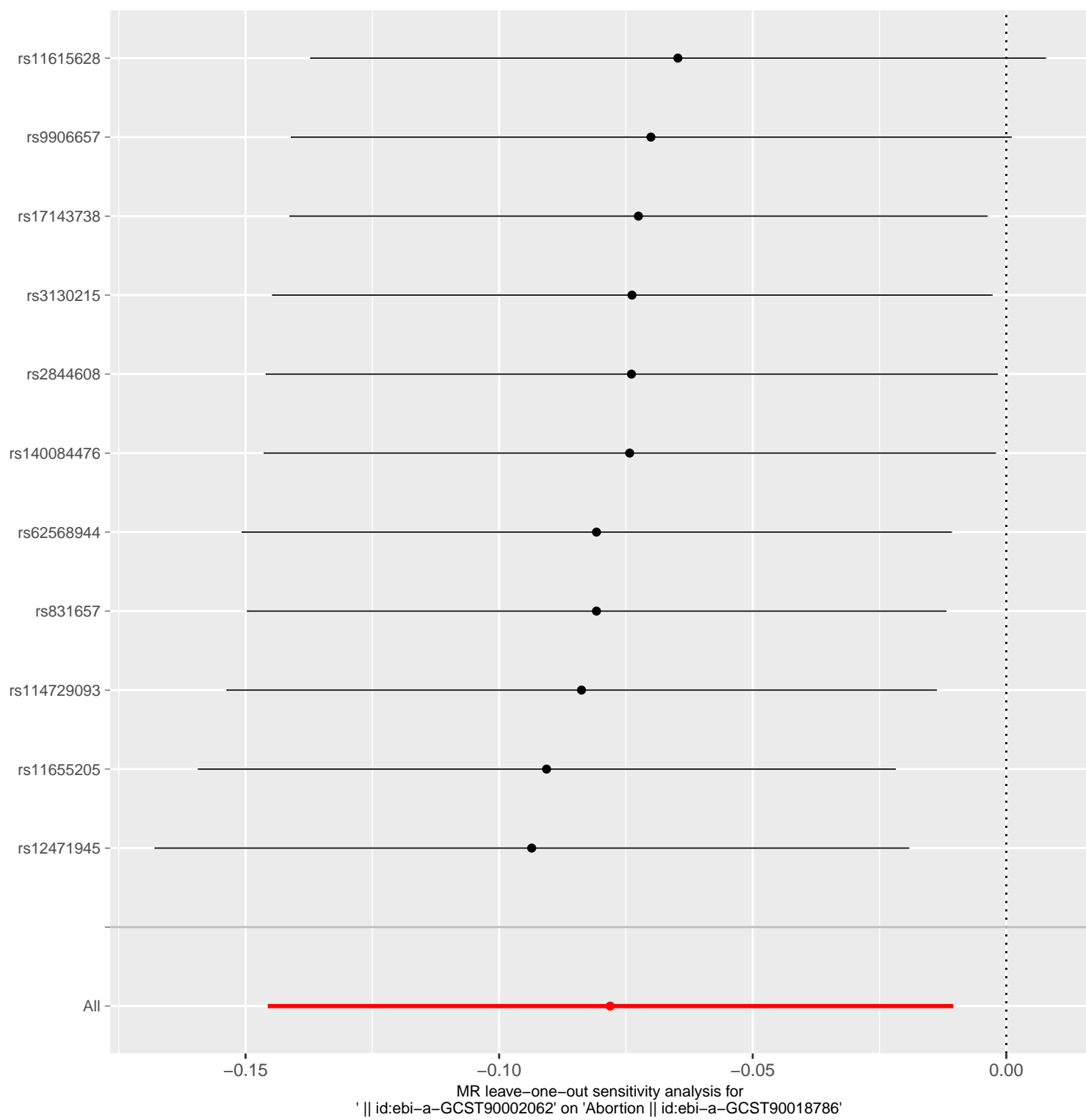

Supplement: S1 File — (ZIP) [file pone.0309088.s001.zip › S1 Fig /ebi-a-GCST90002062/sensitivity-analysis.pdf]

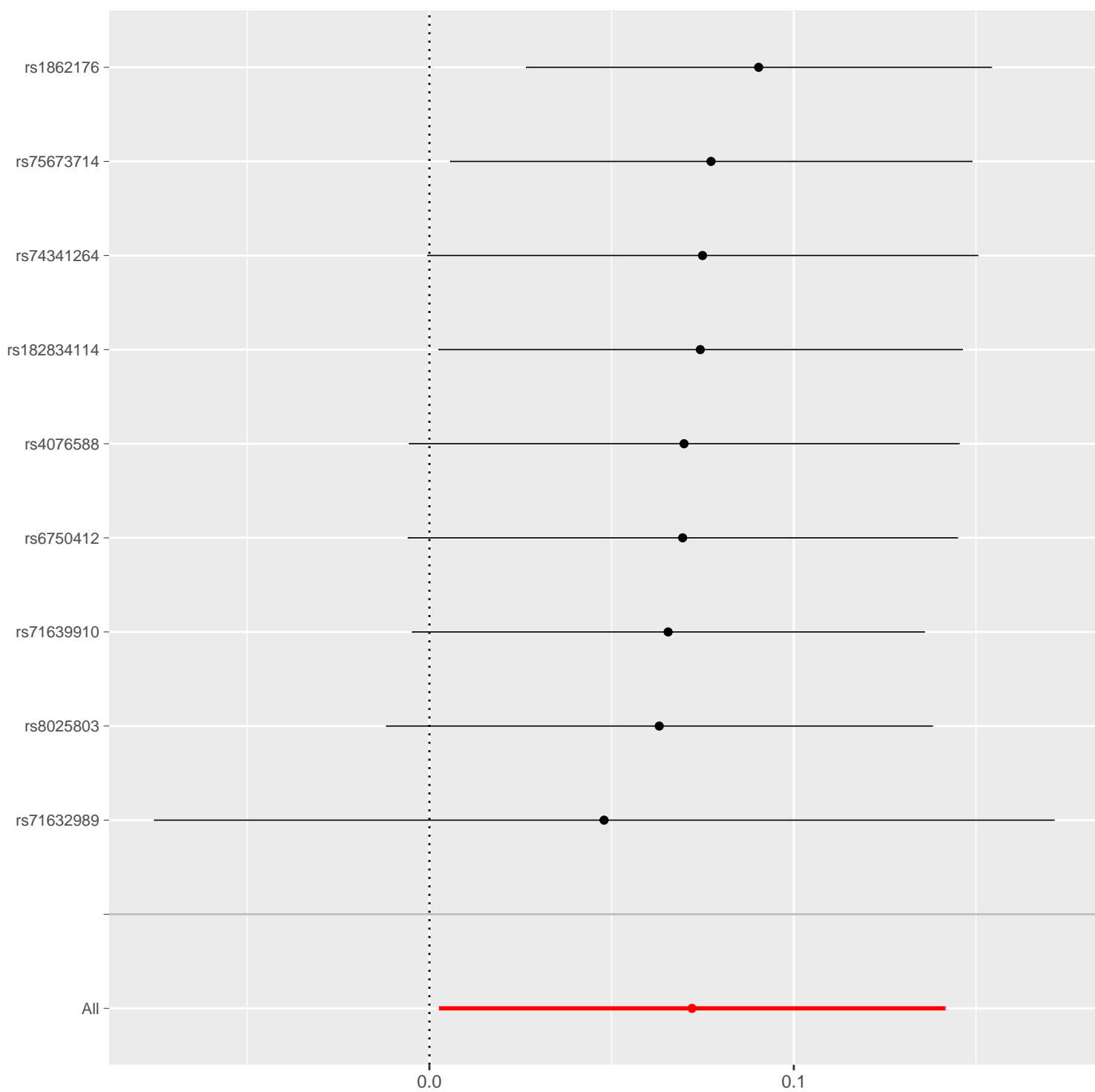

MR leave-one-out sensitivity analysis for  
' || id:ebi-a-GCST90001648' on 'Abortion || id:ebi-a-GCST90018786'

Supplement: S1 File — (ZIP) [file pone.0309088.s001.zip › S1 Fig /ebi-a-GCST90001648/sensitivity-analysis.pdf]

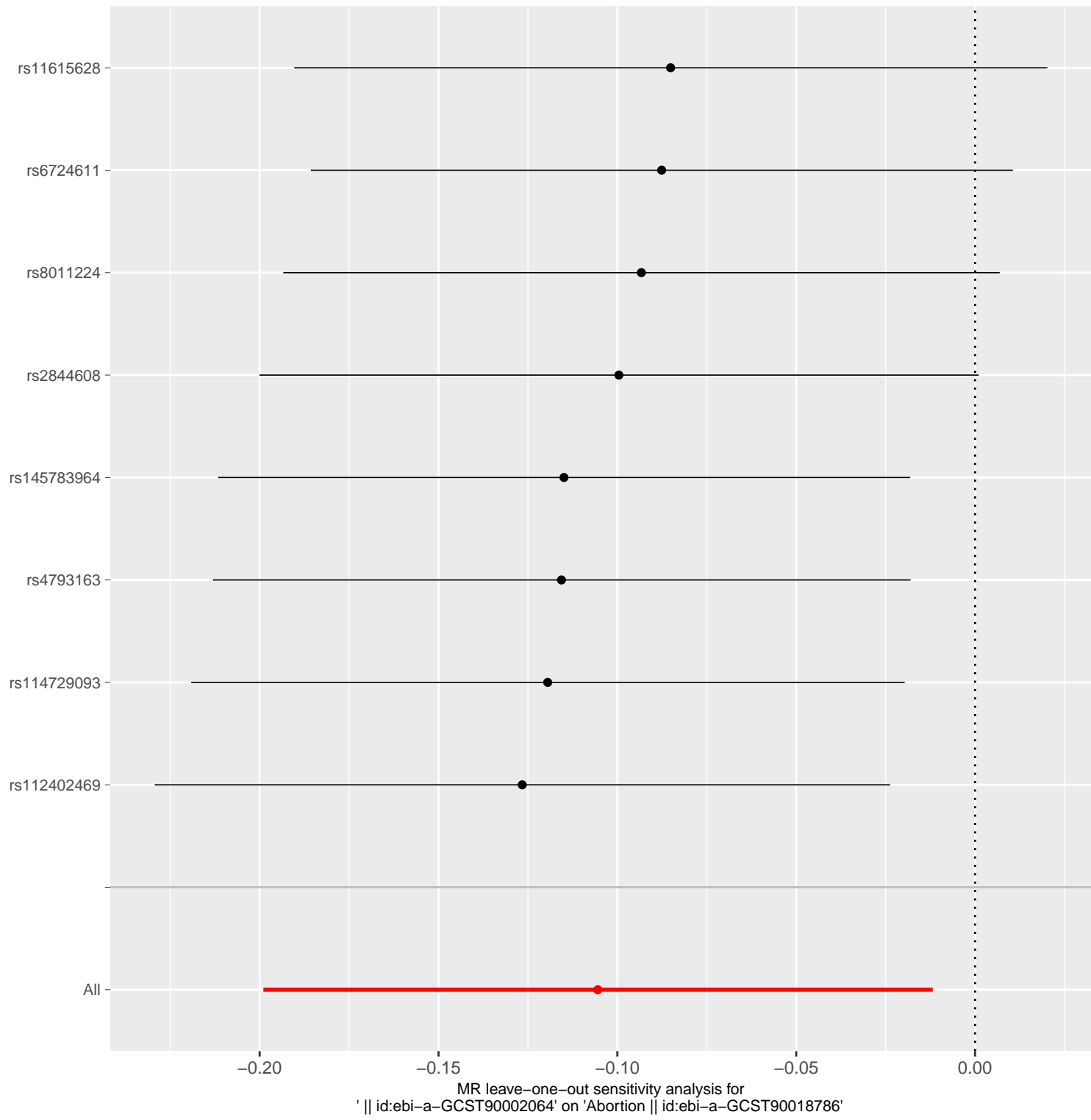

Supplement: S1 File — (ZIP) [file pone.0309088.s001.zip › S1 Fig /ebi-a-GCST90002064/sensitivity-analysis.pdf]

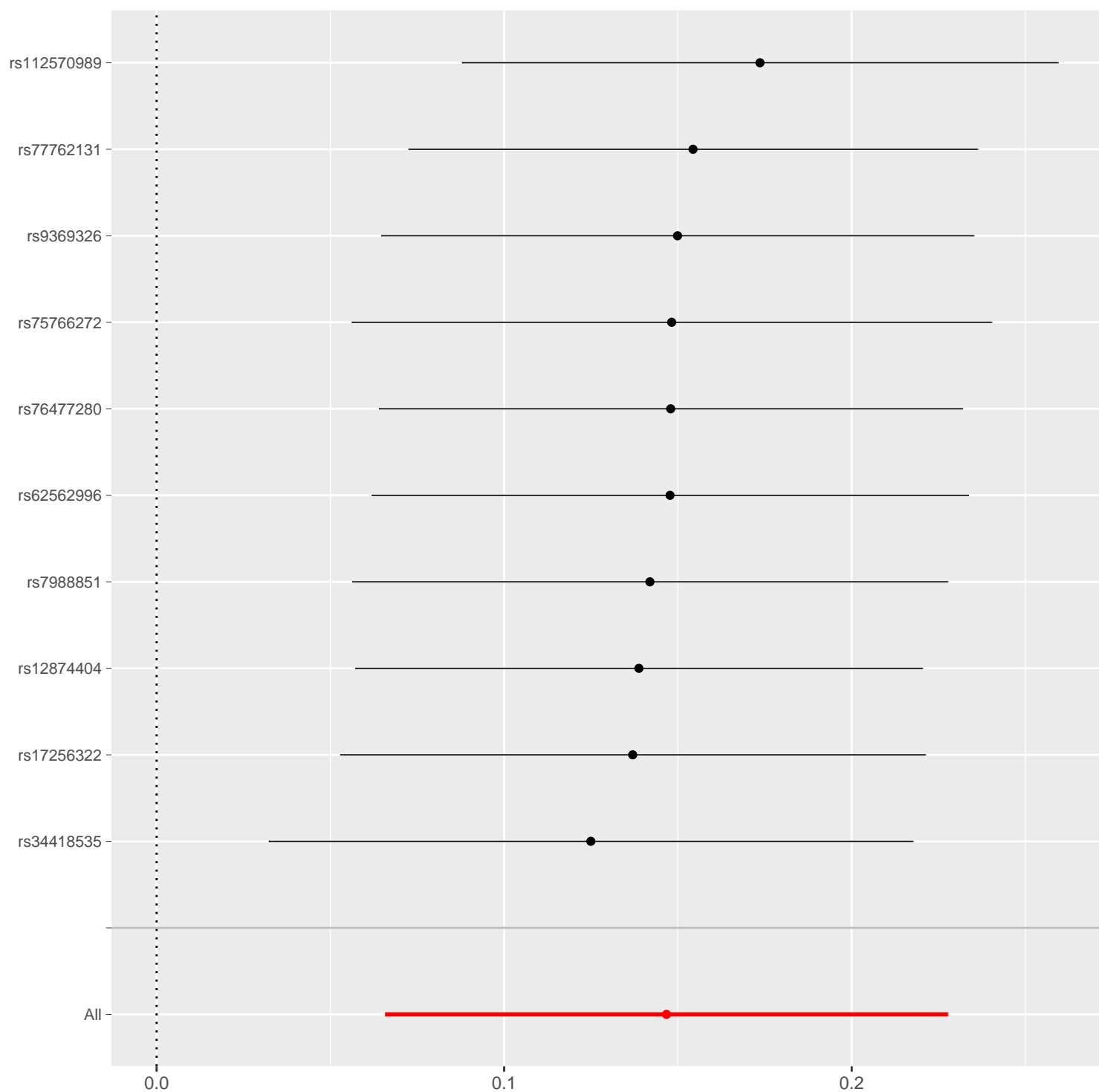

MR leave-one-out sensitivity analysis for  
' || id:ebi-a-GCST90001411' on 'Abortion || id:ebi-a-GCST90018786'

Supplement: S1 File — (ZIP) [file pone.0309088.s001.zip › S1 Fig /ebi-a-GCST90001411/sensitivity-analysis.pdf]

# MR Test

- Inverse variance weighted
- MR Egger
- Simple mode
- Weighted median
- Weighted mode

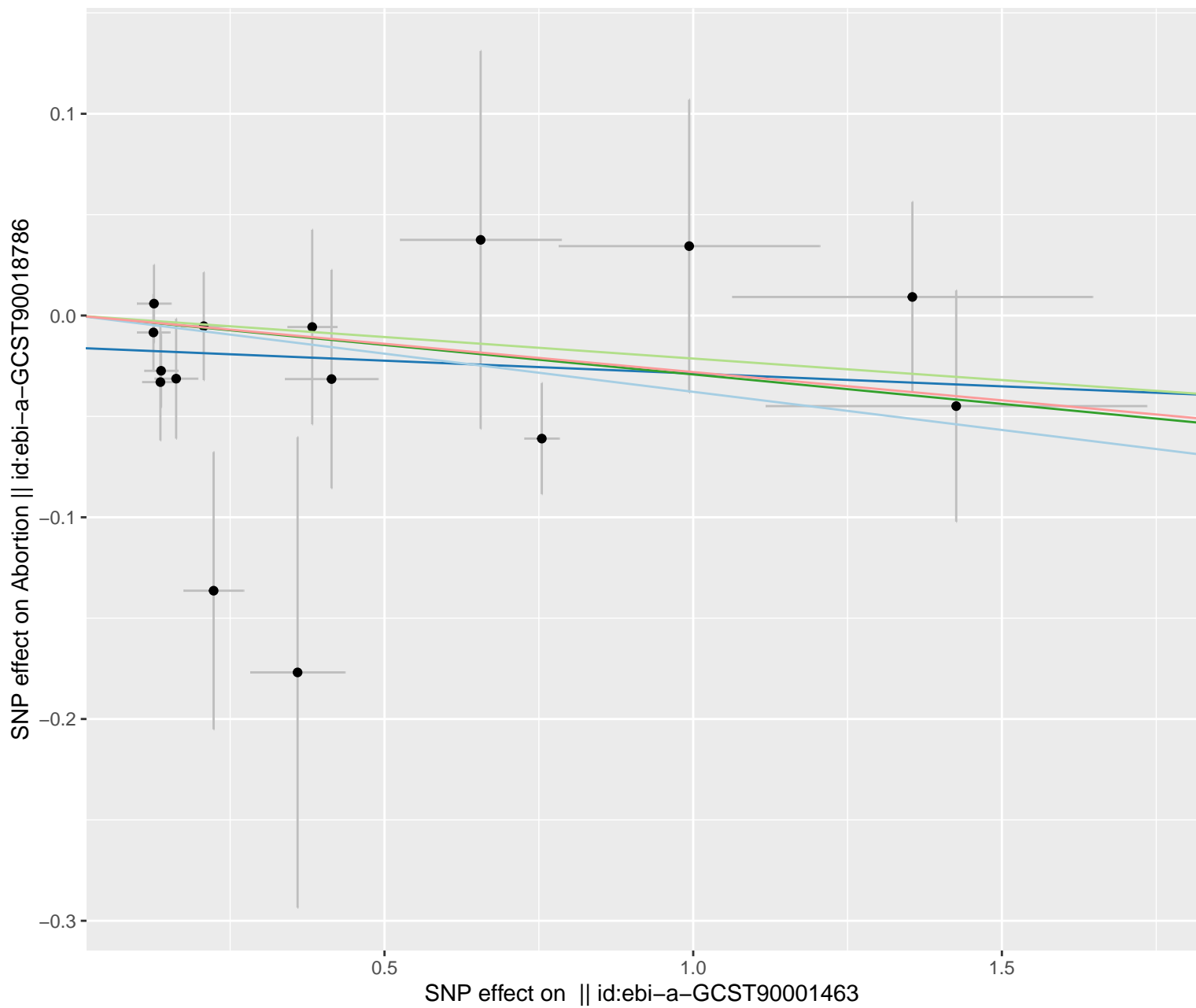

Supplement: S2 File — (ZIP) [file pone.0309088.s002.zip › S2 Fig/ebi-a-GCST90001463/scatter.pdf]

# MR Test

- Inverse variance weighted
- MR Egger
- Simple mode
- Weighted median
- Weighted mode

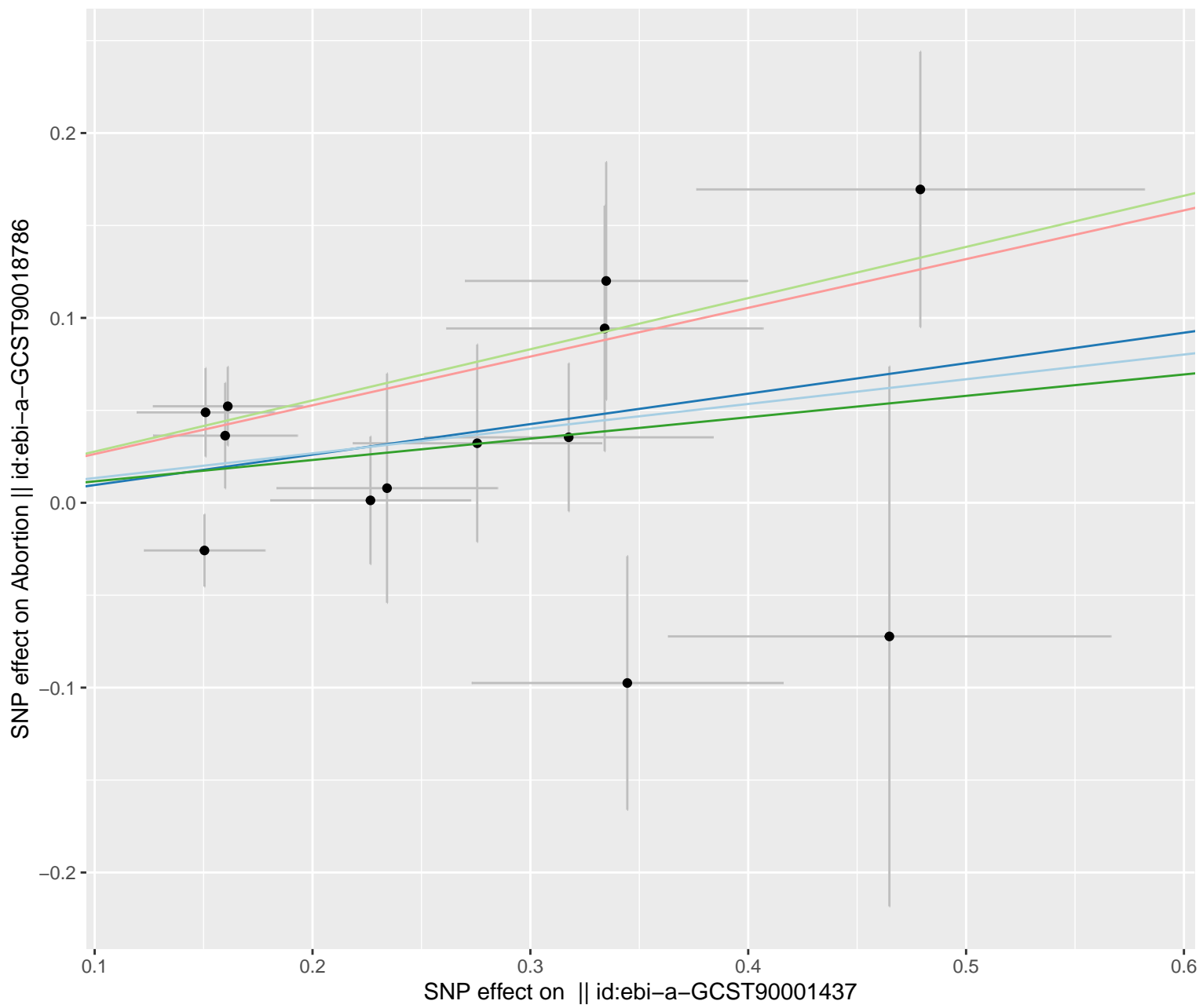

Supplement: S2 File — (ZIP) [file pone.0309088.s002.zip › S2 Fig/ebi-a-GCST90001437/scatter.pdf]

# MR Test

- Inverse variance weighted
- MR Egger
- Simple mode
- Weighted median
- Weighted mode

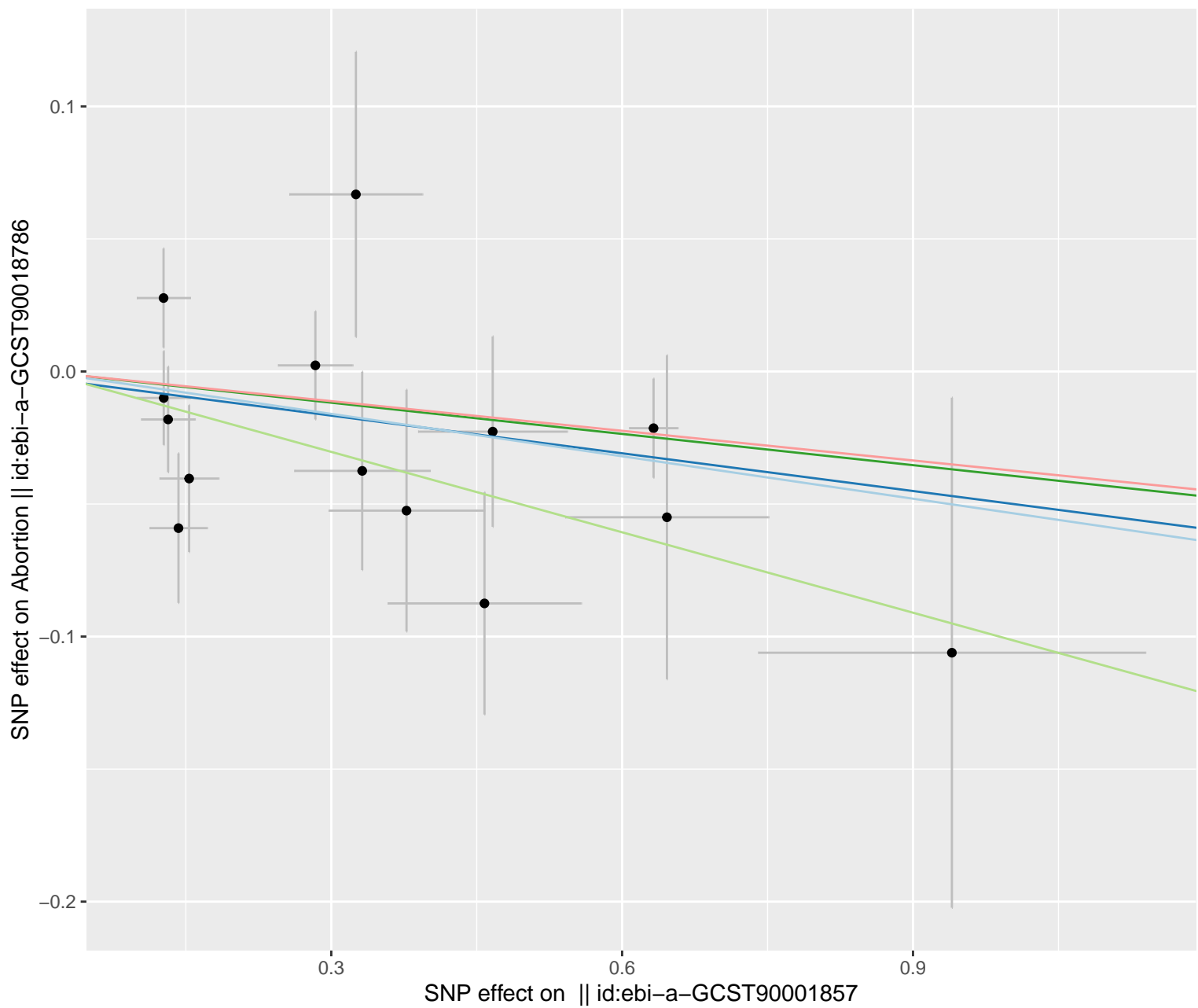

Supplement: S2 File — (ZIP) [file pone.0309088.s002.zip › S2 Fig/ebi-a-GCST90001857/scatter.pdf]

# MR Test

- Inverse variance weighted
- MR Egger
- Simple mode
- Weighted median
- Weighted mode

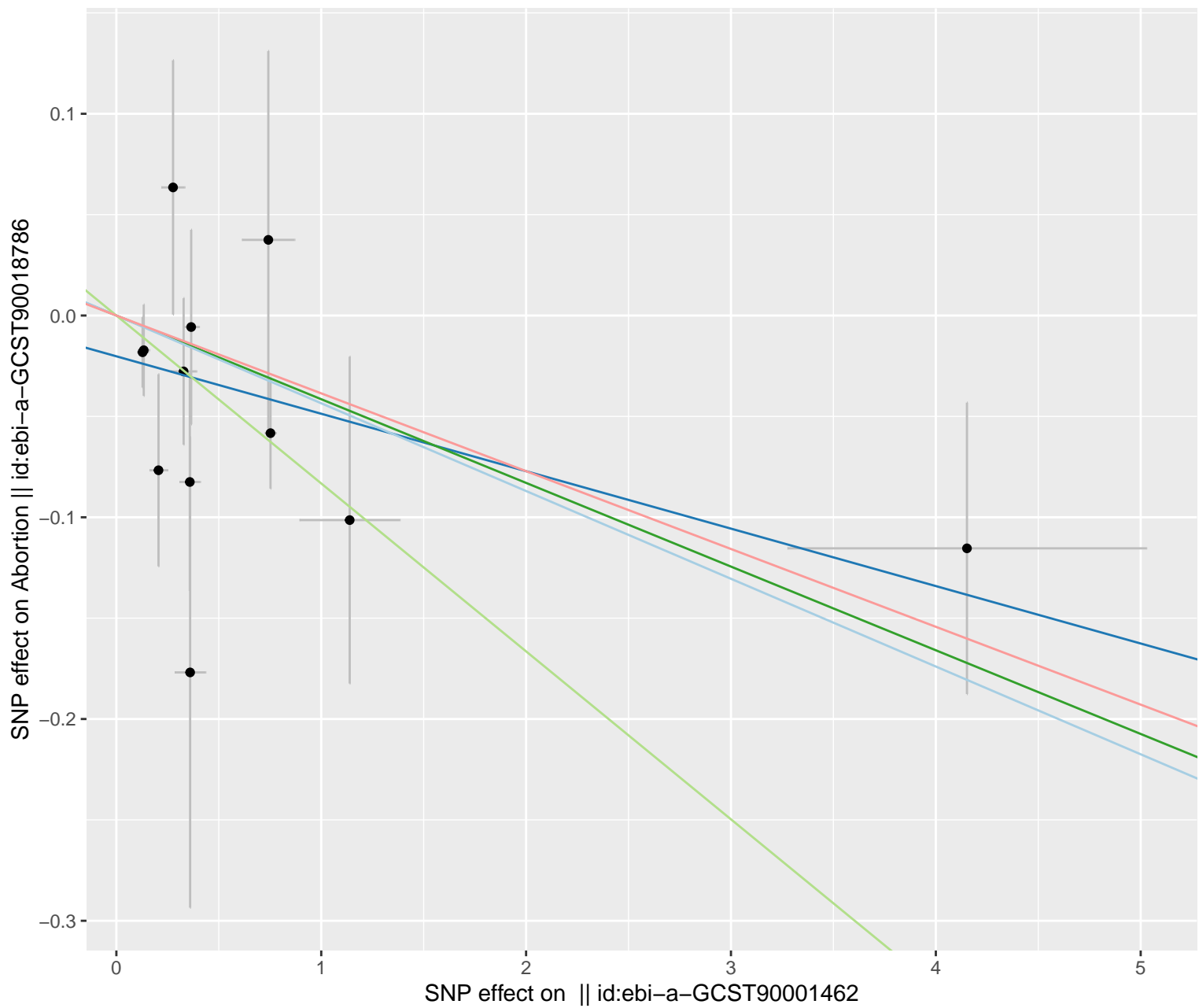

Supplement: S2 File — (ZIP) [file pone.0309088.s002.zip › S2 Fig/ebi-a-GCST90001462/scatter.pdf]

# MR Test

- Inverse variance weighted
- MR Egger
- Simple mode
- Weighted median
- Weighted mode

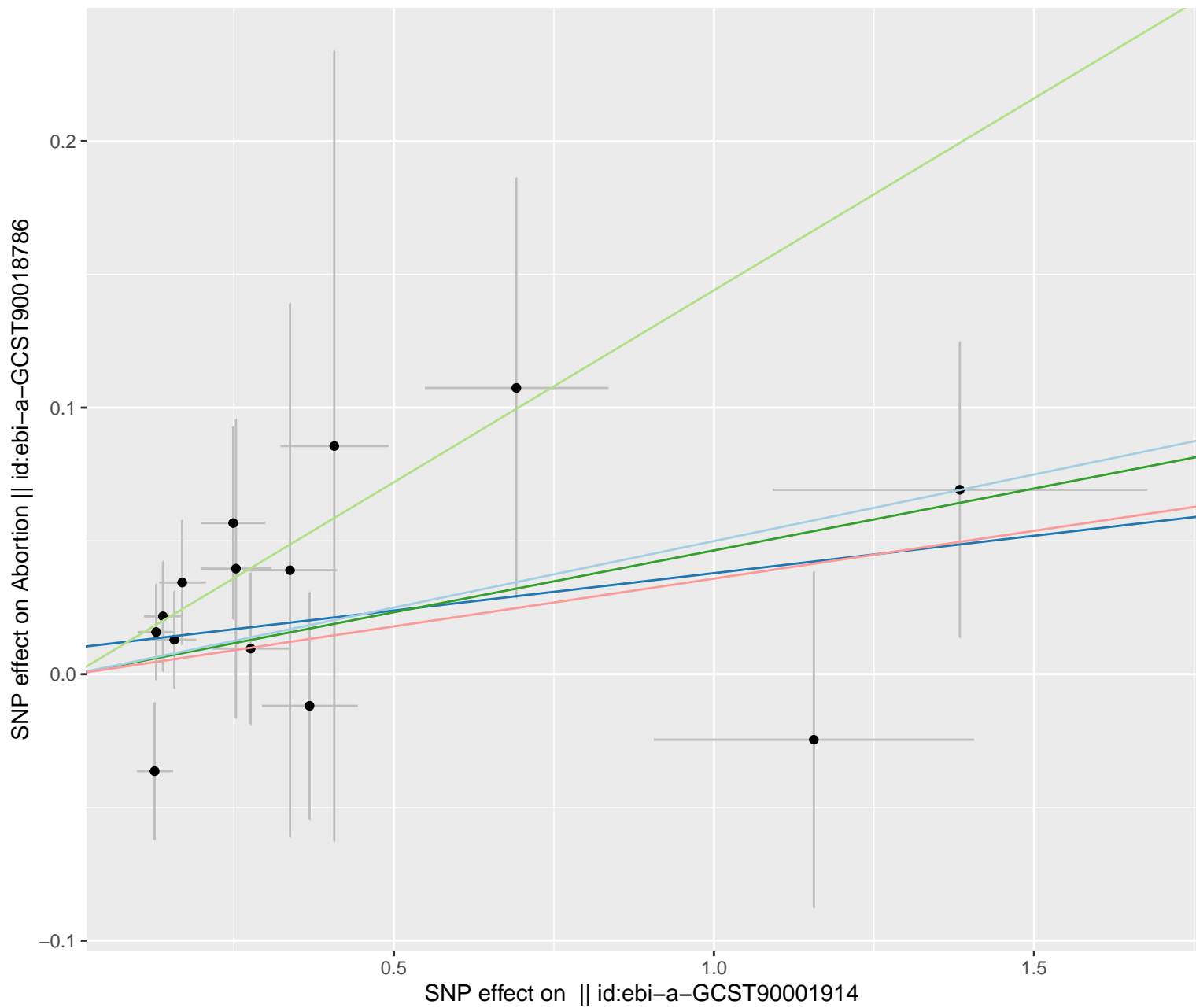

Supplement: S2 File — (ZIP) [file pone.0309088.s002.zip › S2 Fig/ebi-a-GCST90001914/scatter.pdf]

# MR Test

- Inverse variance weighted
- MR Egger
- Simple mode
- Weighted median
- Weighted mode

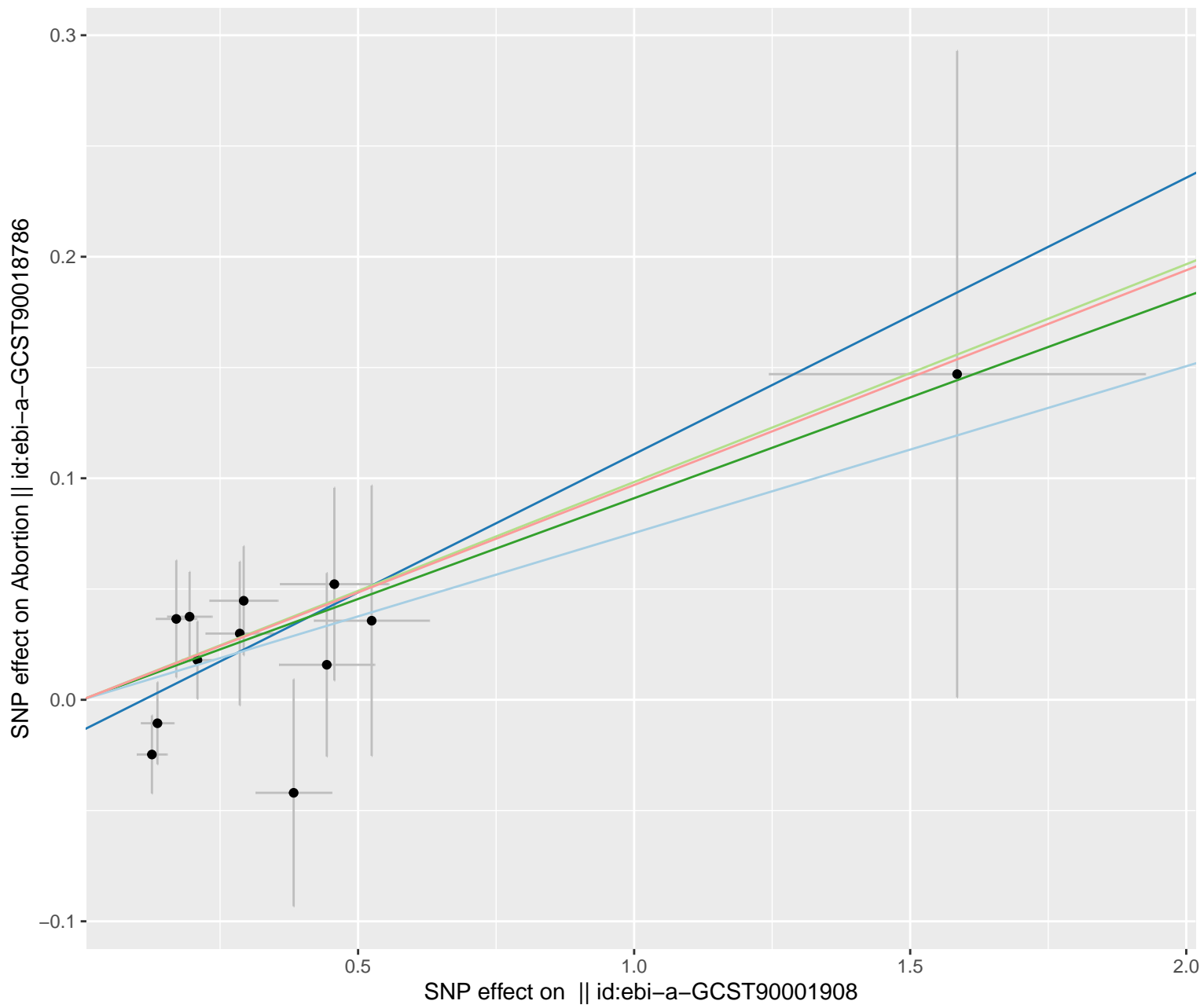

Supplement: S2 File — (ZIP) [file pone.0309088.s002.zip › S2 Fig/ebi-a-GCST90001908/scatter.pdf]

# MR Test

- Inverse variance weighted
- MR Egger
- Simple mode
- Weighted median
- Weighted mode

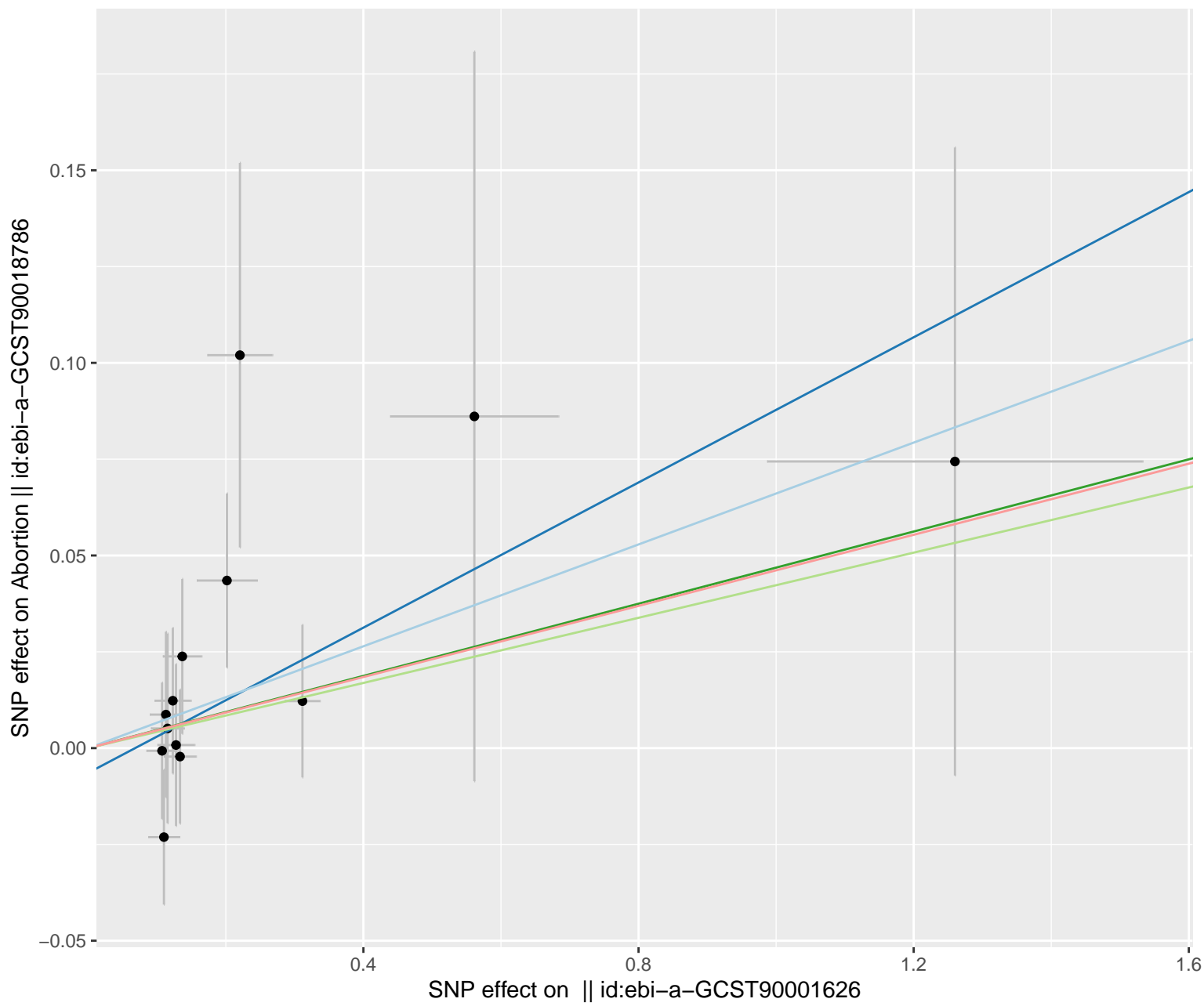

Supplement: S2 File — (ZIP) [file pone.0309088.s002.zip › S2 Fig/ebi-a-GCST90001626/scatter.pdf]

# MR Test

- Inverse variance weighted
- MR Egger
- Simple mode
- Weighted median
- Weighted mode

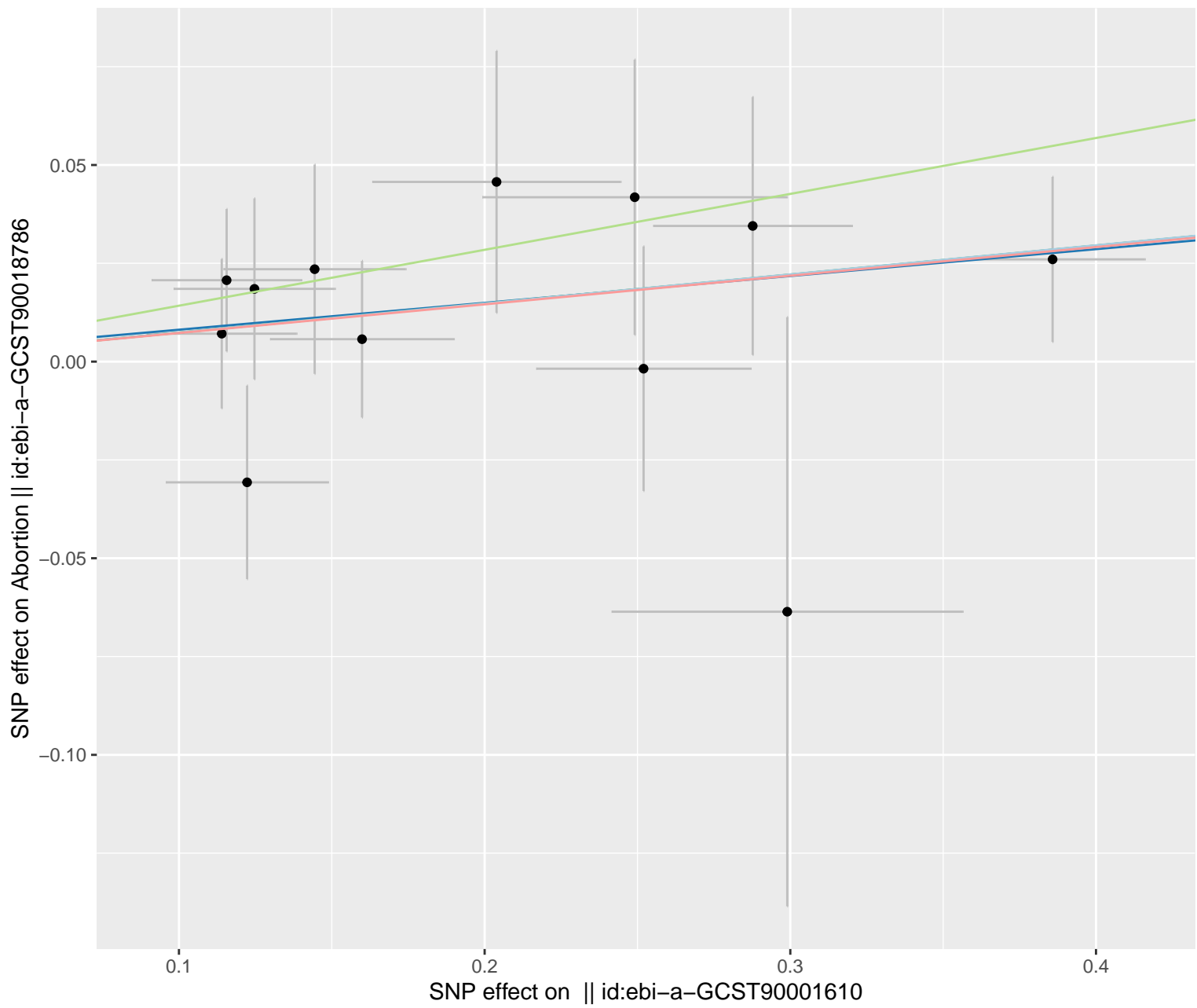

Supplement: S2 File — (ZIP) [file pone.0309088.s002.zip › S2 Fig/ebi-a-GCST90001610/scatter.pdf]

# MR Test

- Inverse variance weighted
- MR Egger
- Simple mode
- Weighted median
- Weighted mode

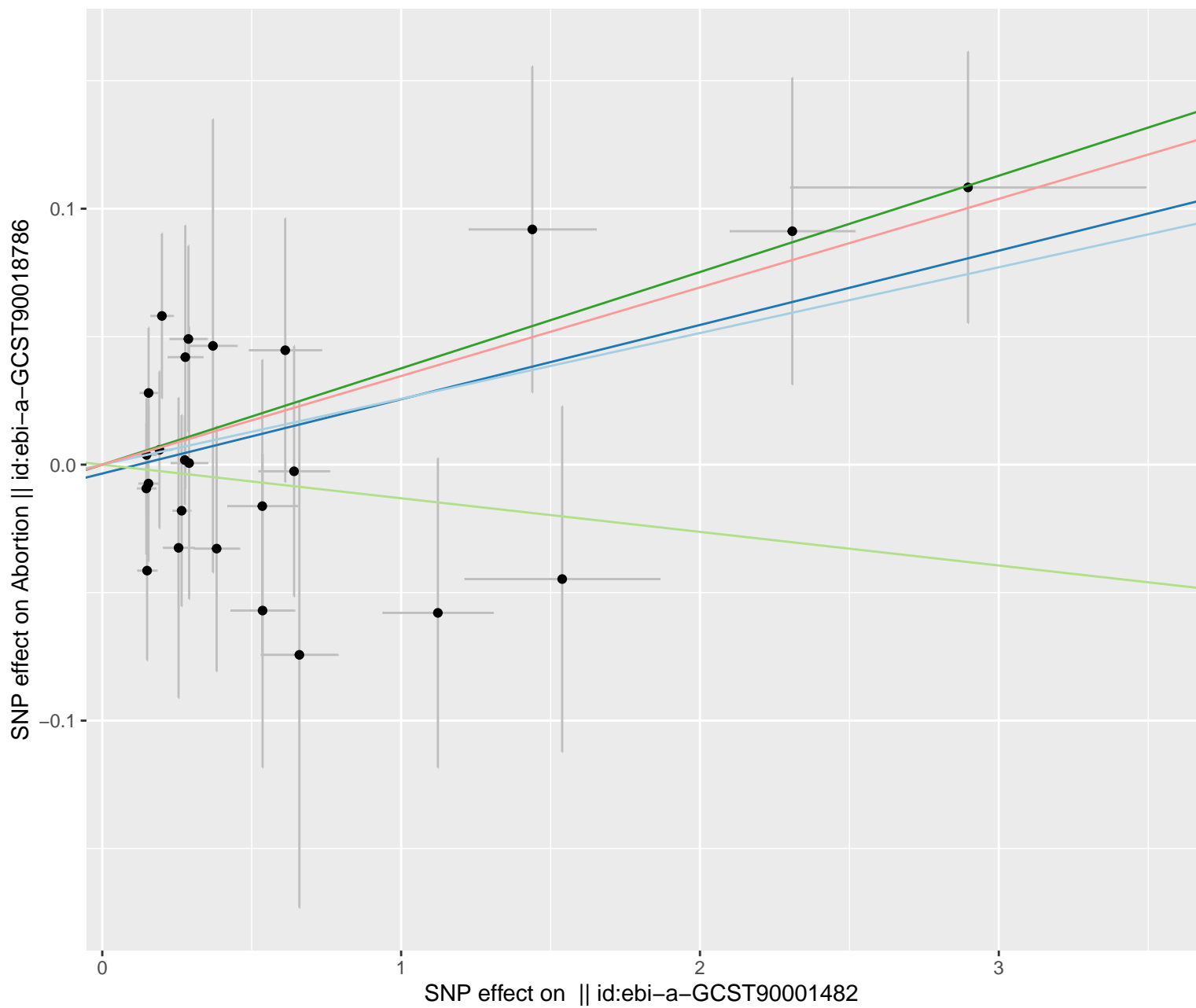

Supplement: S2 File — (ZIP) [file pone.0309088.s002.zip › S2 Fig/ebi-a-GCST90001482/scatter.pdf]

# MR Test

- Inverse variance weighted
- MR Egger
- Simple mode
- Weighted median
- Weighted mode

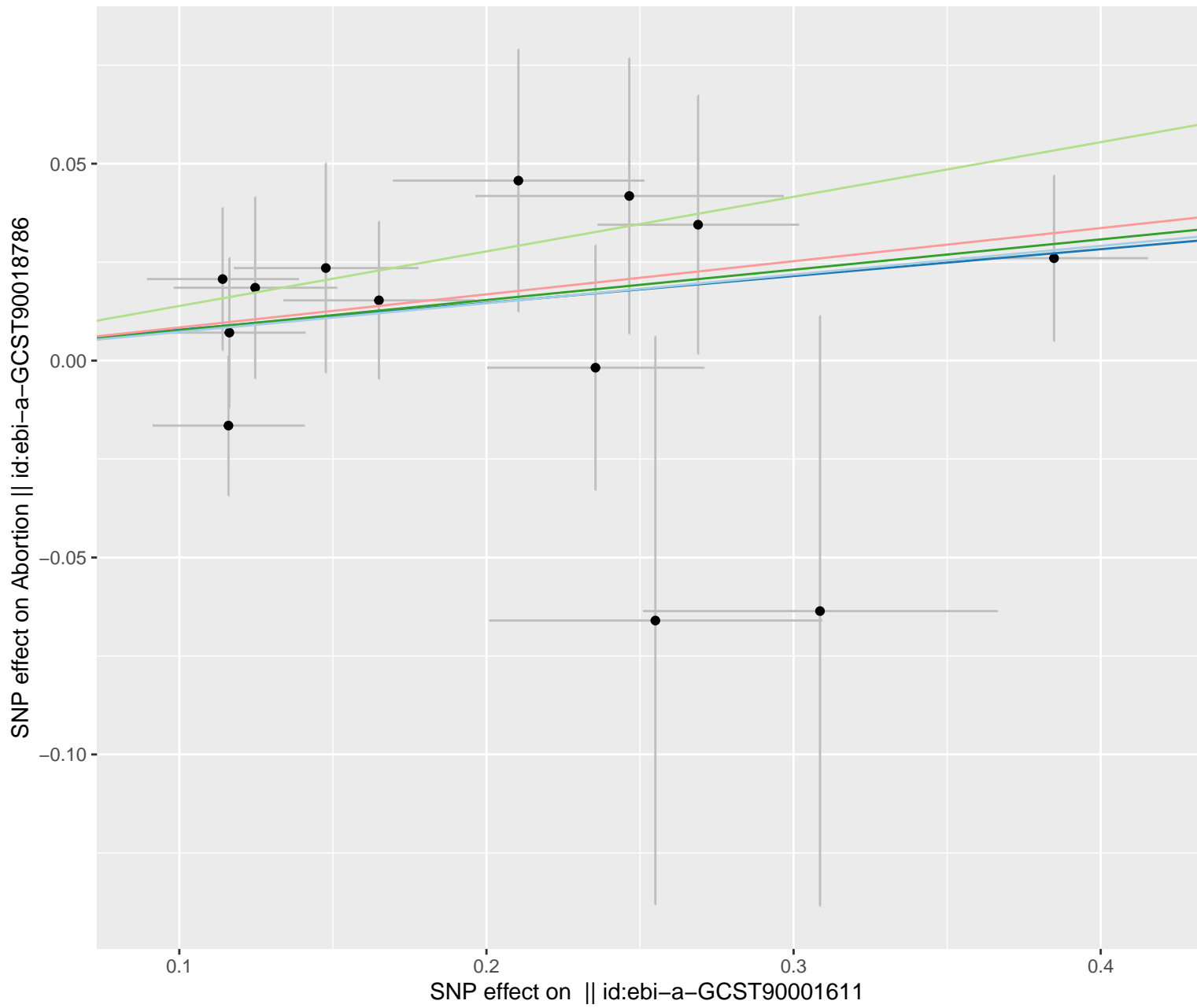

Supplement: S2 File — (ZIP) [file pone.0309088.s002.zip › S2 Fig/ebi-a-GCST90001611/scatter.pdf]

# MR Test

- Inverse variance weighted
- MR Egger
- Simple mode
- Weighted median
- Weighted mode

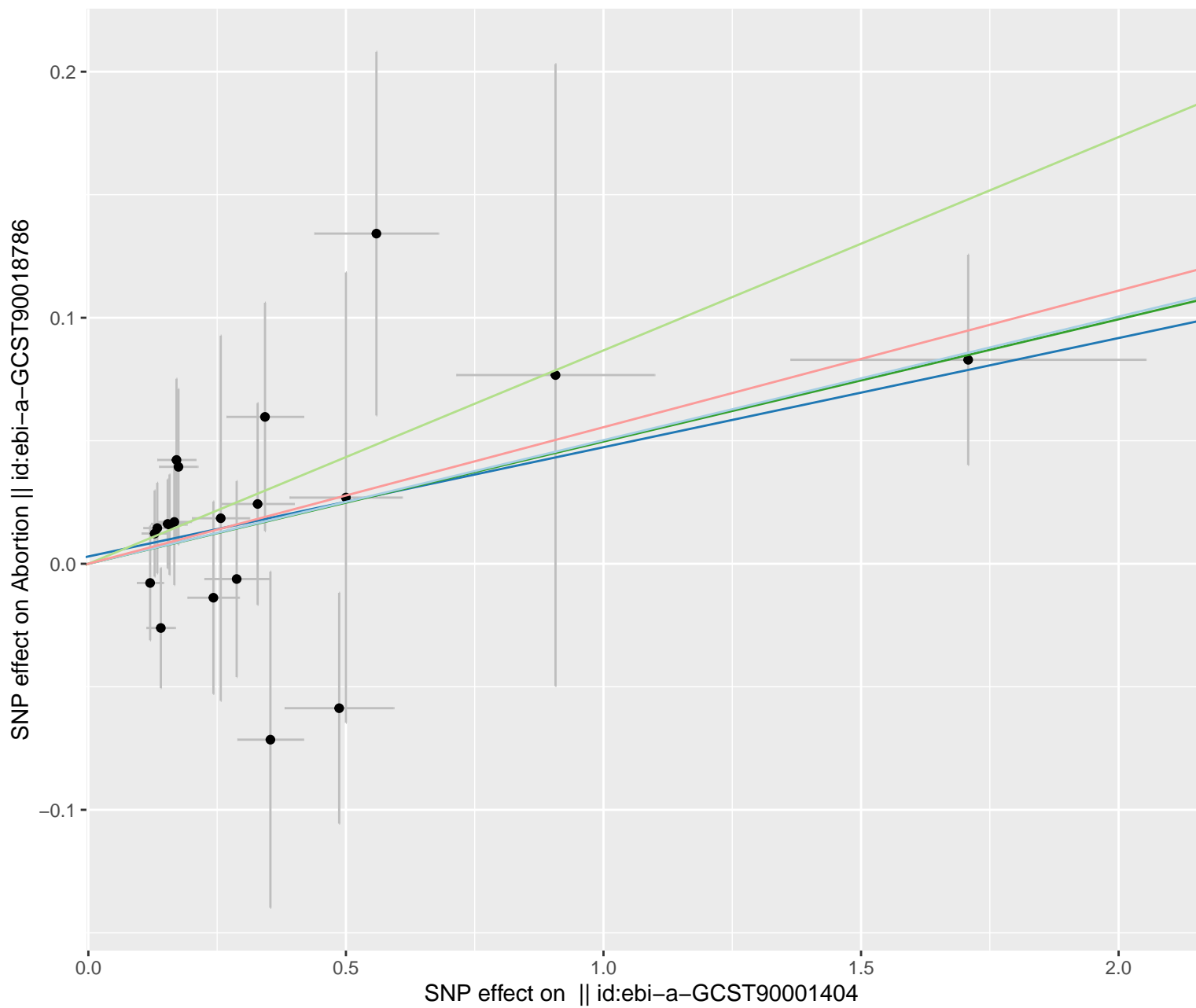

Supplement: S2 File — (ZIP) [file pone.0309088.s002.zip › S2 Fig/ebi-a-GCST90001404/scatter.pdf]

# MR Test

- Inverse variance weighted
- MR Egger
- Simple mode
- Weighted median
- Weighted mode

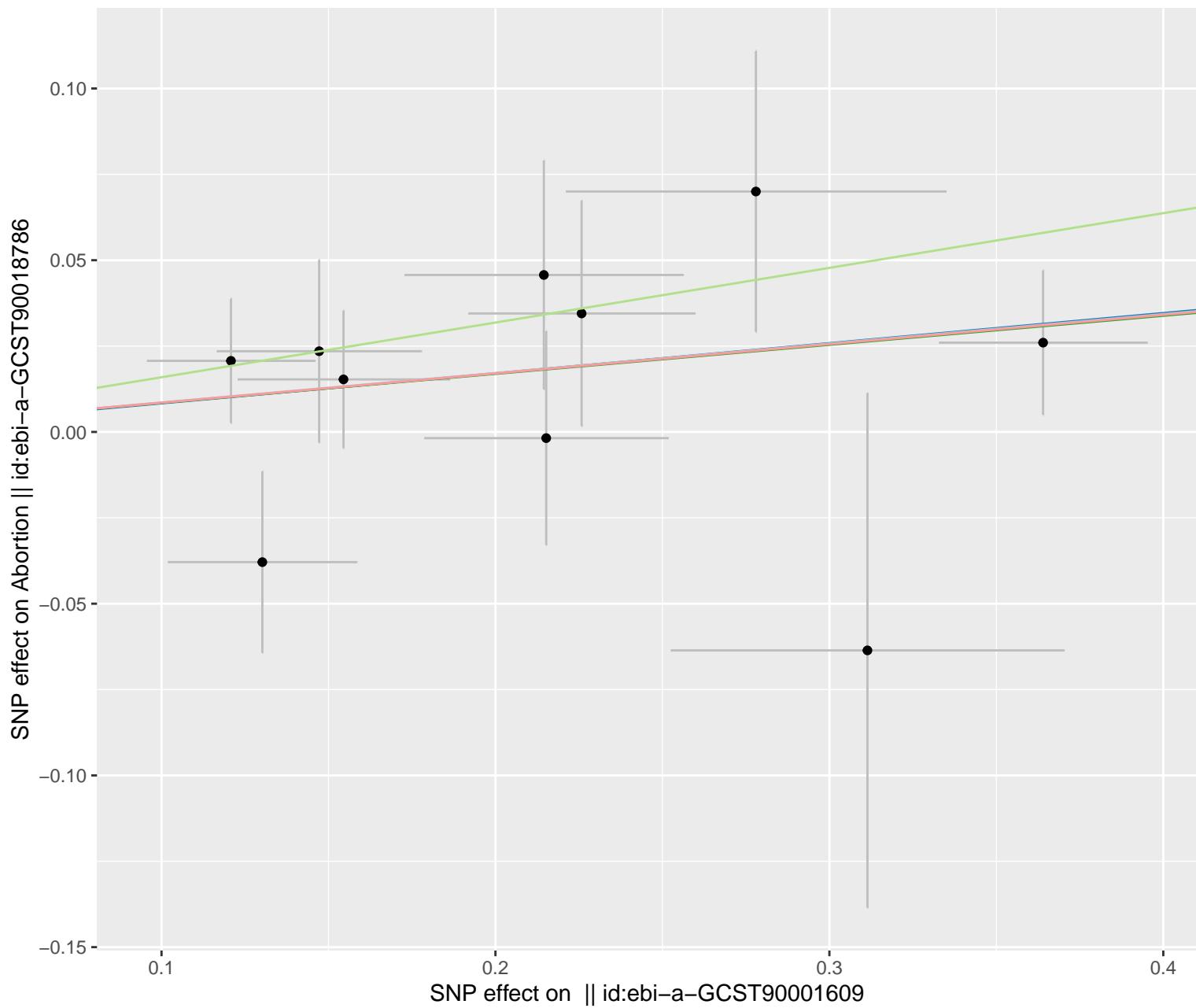

Supplement: S2 File — (ZIP) [file pone.0309088.s002.zip › S2 Fig/ebi-a-GCST90001609/scatter.pdf]

# MR Test

- Inverse variance weighted
- MR Egger
- Simple mode
- Weighted median
- Weighted mode

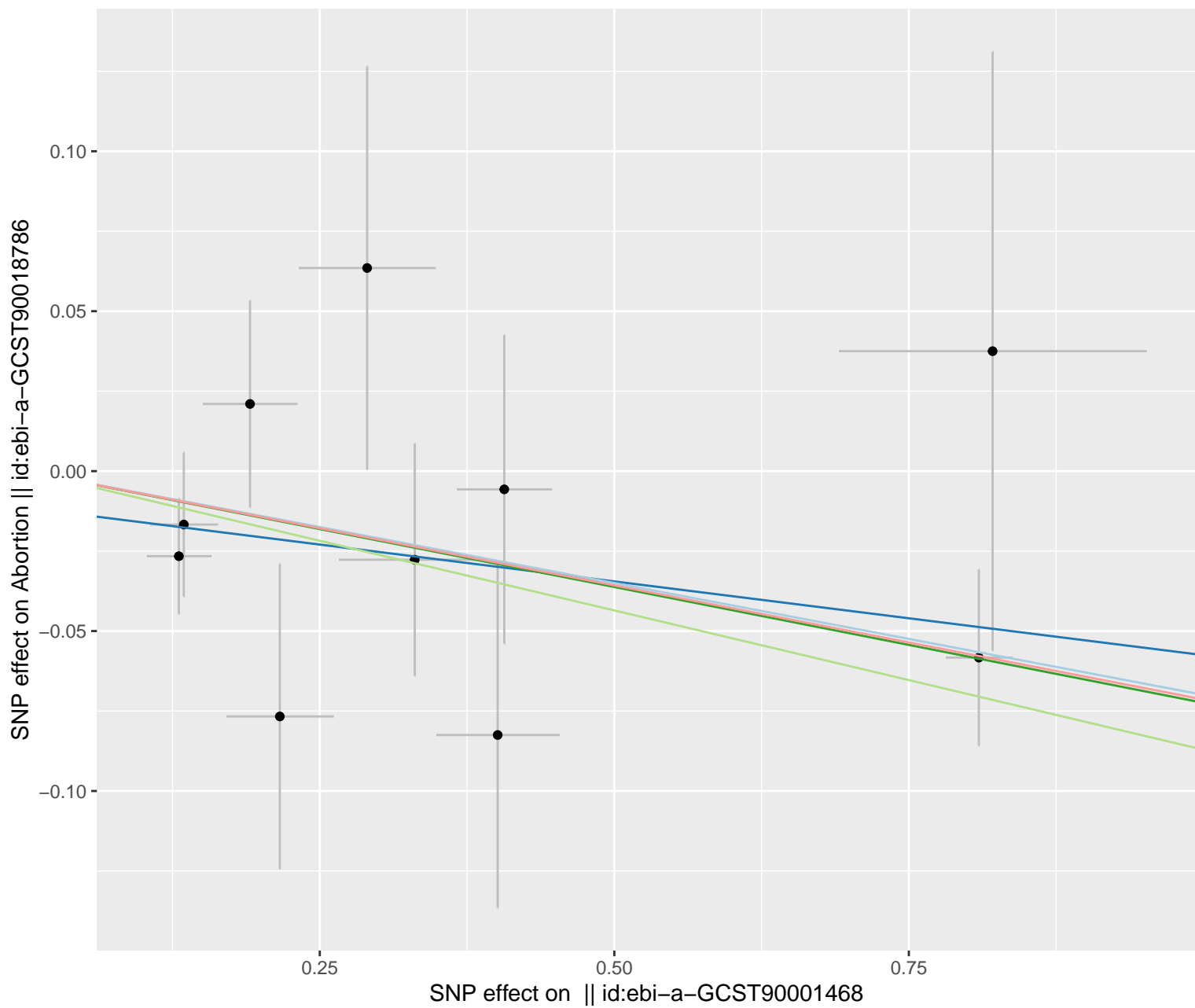

Supplement: S2 File — (ZIP) [file pone.0309088.s002.zip › S2 Fig/ebi-a-GCST90001468/scatter.pdf]

# MR Test

- Inverse variance weighted
- MR Egger
- Simple mode
- Weighted median
- Weighted mode

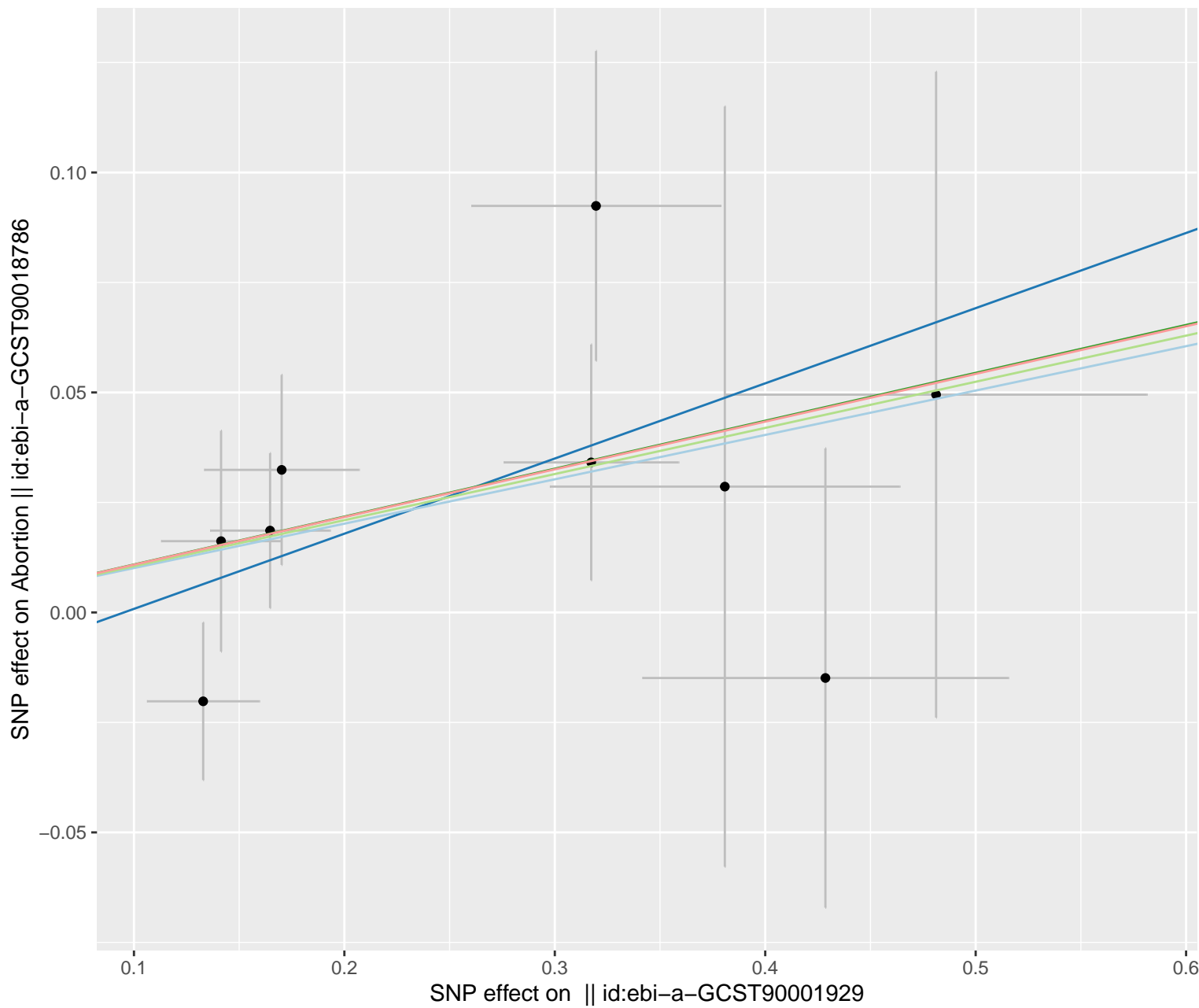

Supplement: S2 File — (ZIP) [file pone.0309088.s002.zip › S2 Fig/ebi-a-GCST90001929/scatter.pdf]

# MR Test

- Inverse variance weighted
- MR Egger
- Simple mode
- Weighted median
- Weighted mode

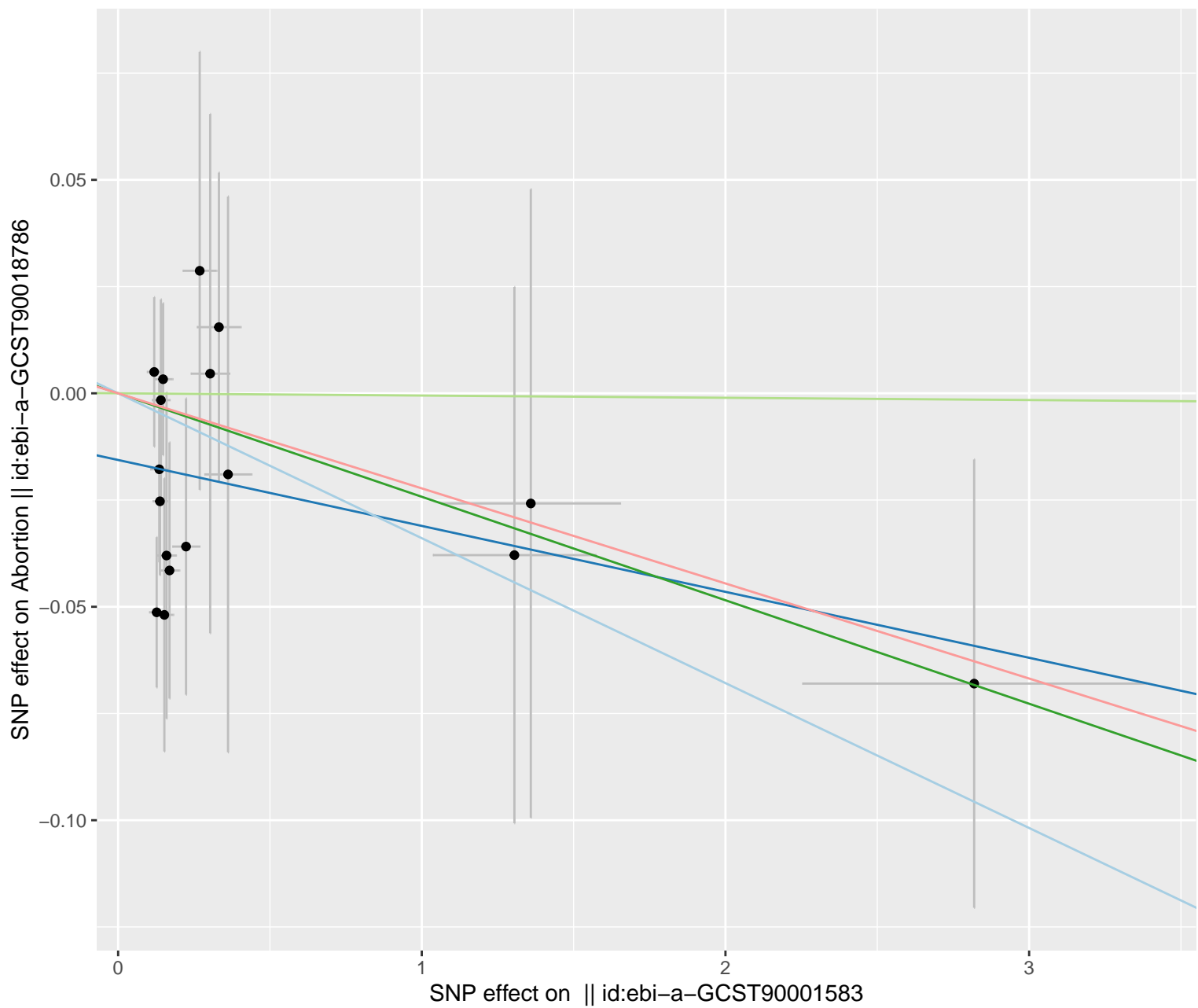

Supplement: S2 File — (ZIP) [file pone.0309088.s002.zip › S2 Fig/ebi-a-GCST90001583/scatter.pdf]

# MR Test

- Inverse variance weighted
- MR Egger
- Simple mode
- Weighted median
- Weighted mode

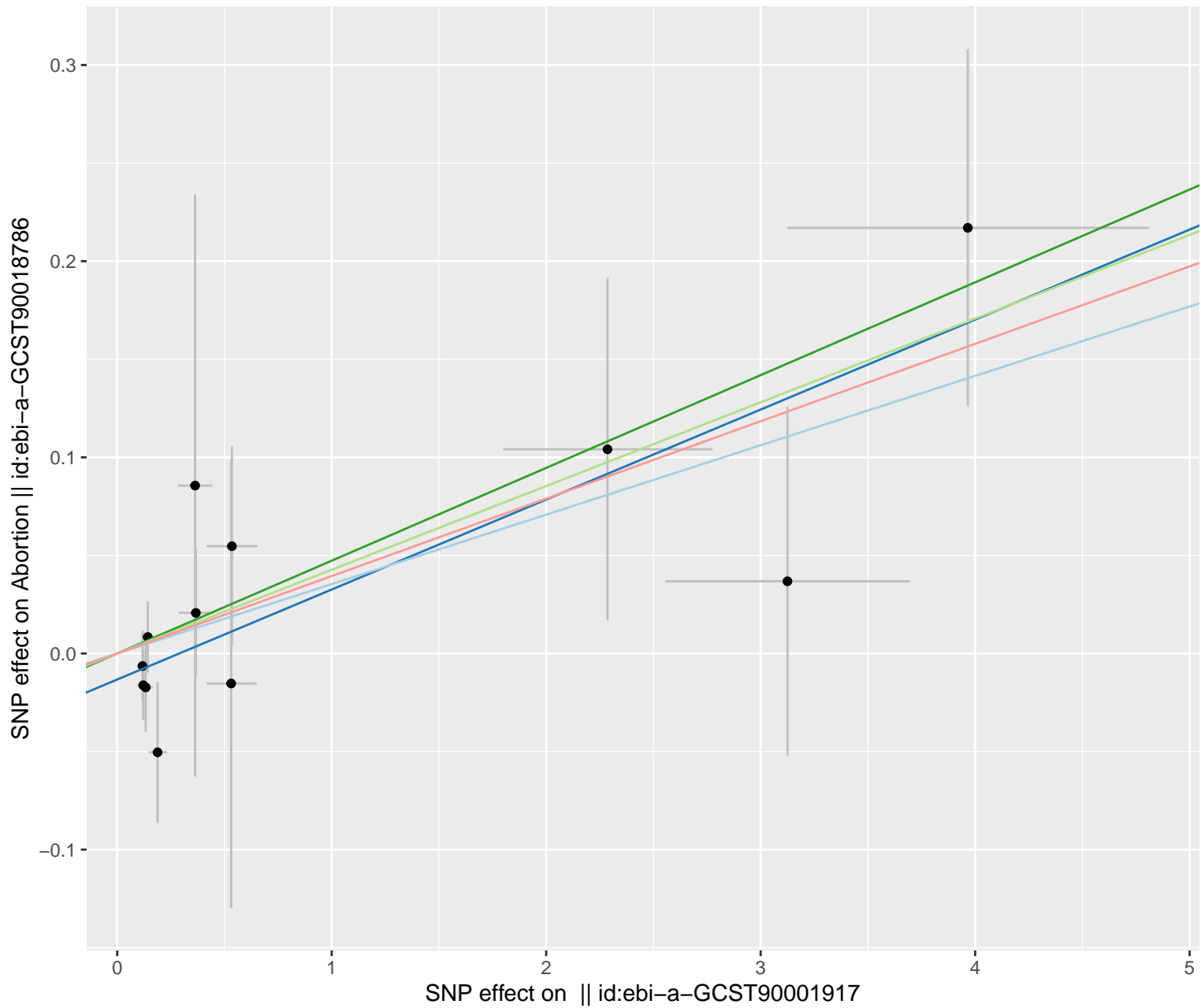

Supplement: S2 File — (ZIP) [file pone.0309088.s002.zip › S2 Fig/ebi-a-GCST90001917/scatter.pdf]

# MR Test

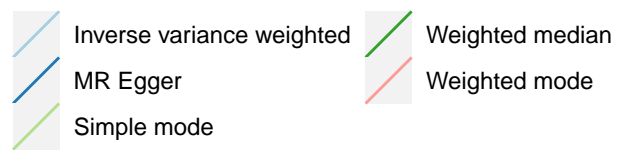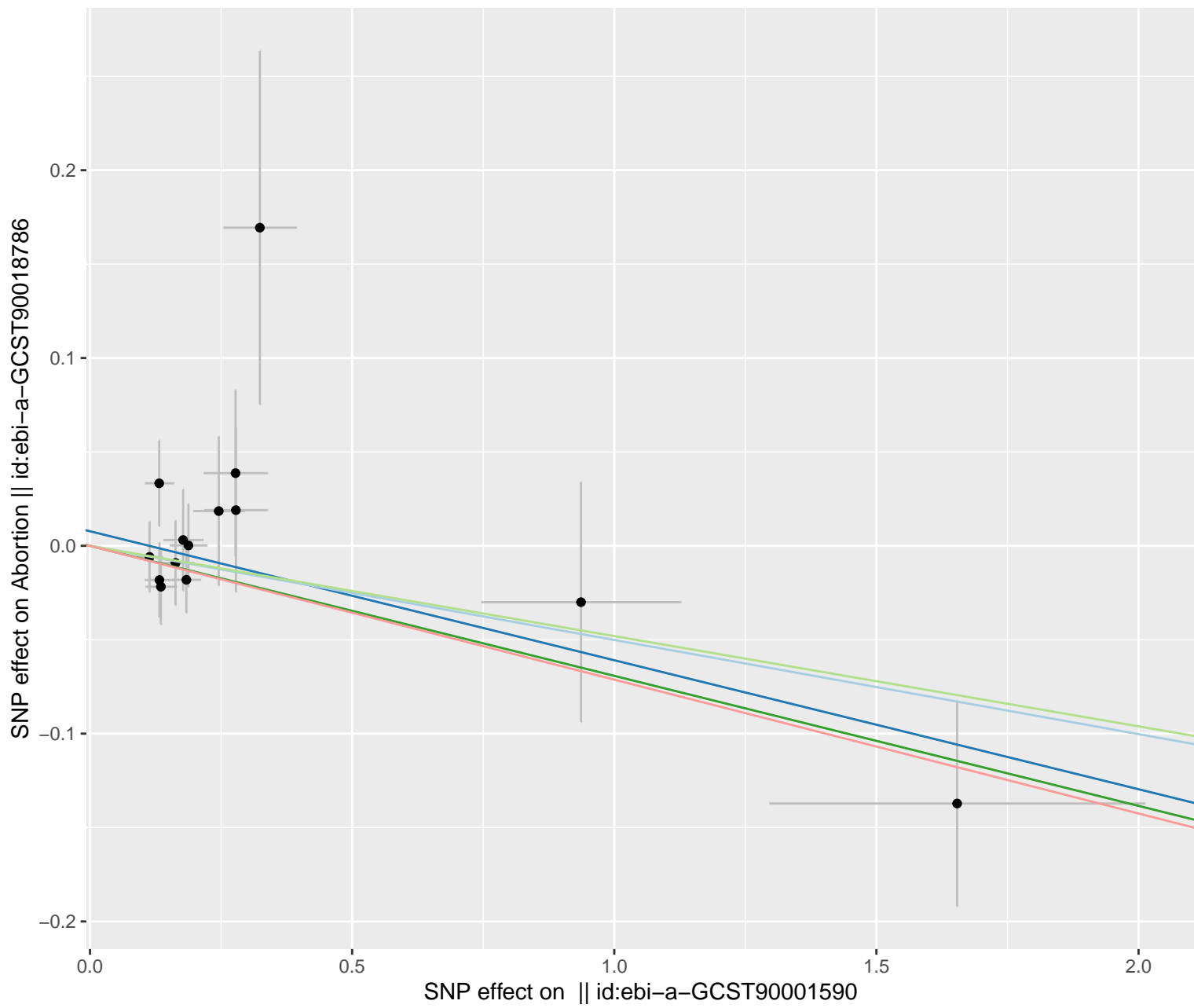

Supplement: S2 File — (ZIP) [file pone.0309088.s002.zip › S2 Fig/ebi-a-GCST90001590/scatter.pdf]

# MR Test

- Inverse variance weighted
- MR Egger
- Simple mode
- Weighted median
- Weighted mode

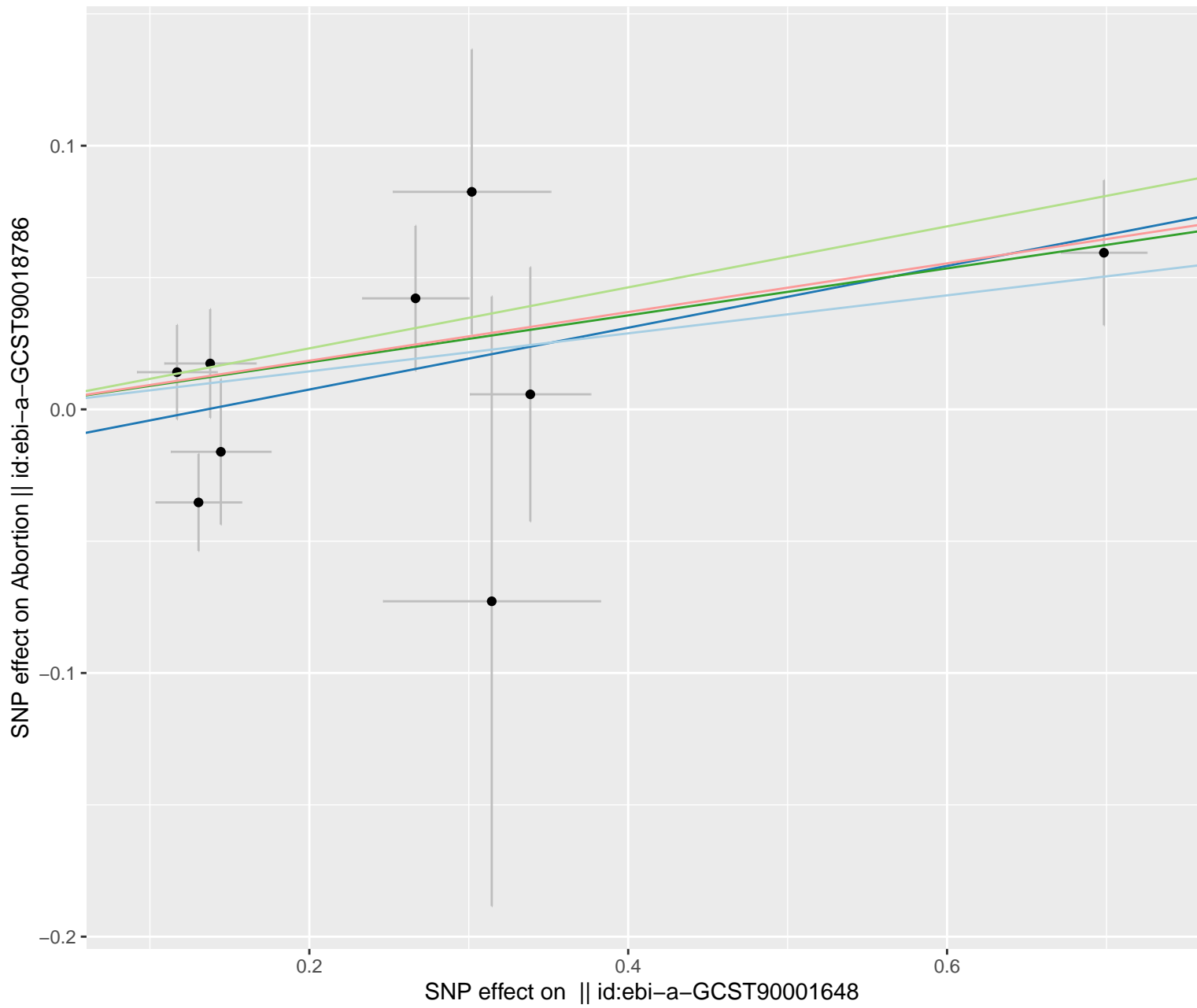

Supplement: S2 File — (ZIP) [file pone.0309088.s002.zip › S2 Fig/ebi-a-GCST90001648/scatter.pdf]

# MR Test

- Inverse variance weighted
- MR Egger
- Simple mode
- Weighted median
- Weighted mode

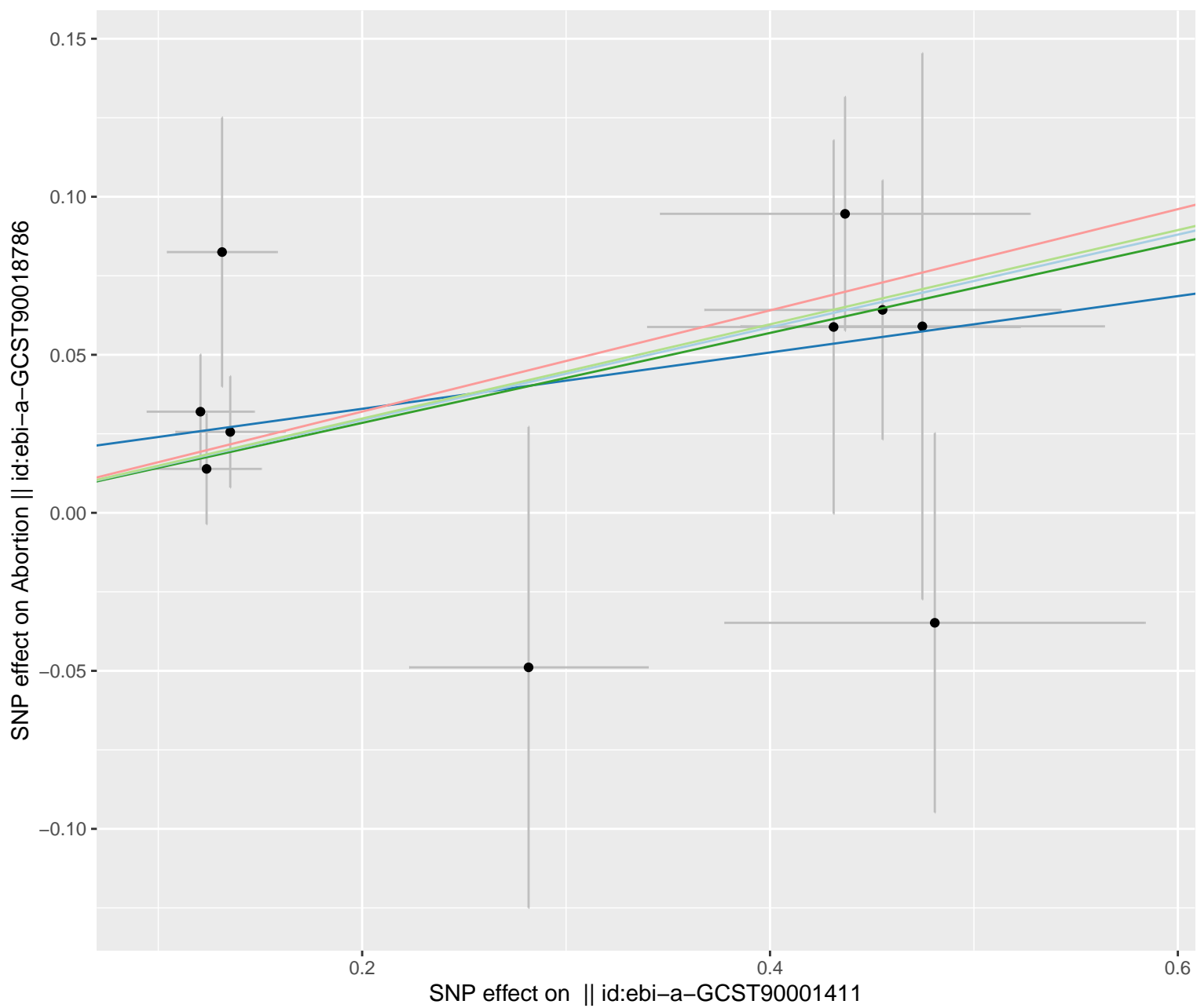

Supplement: S2 File — (ZIP) [file pone.0309088.s002.zip › S2 Fig/ebi-a-GCST90001411/scatter.pdf]

# MR Method

- Inverse variance weighted
- MR Egger

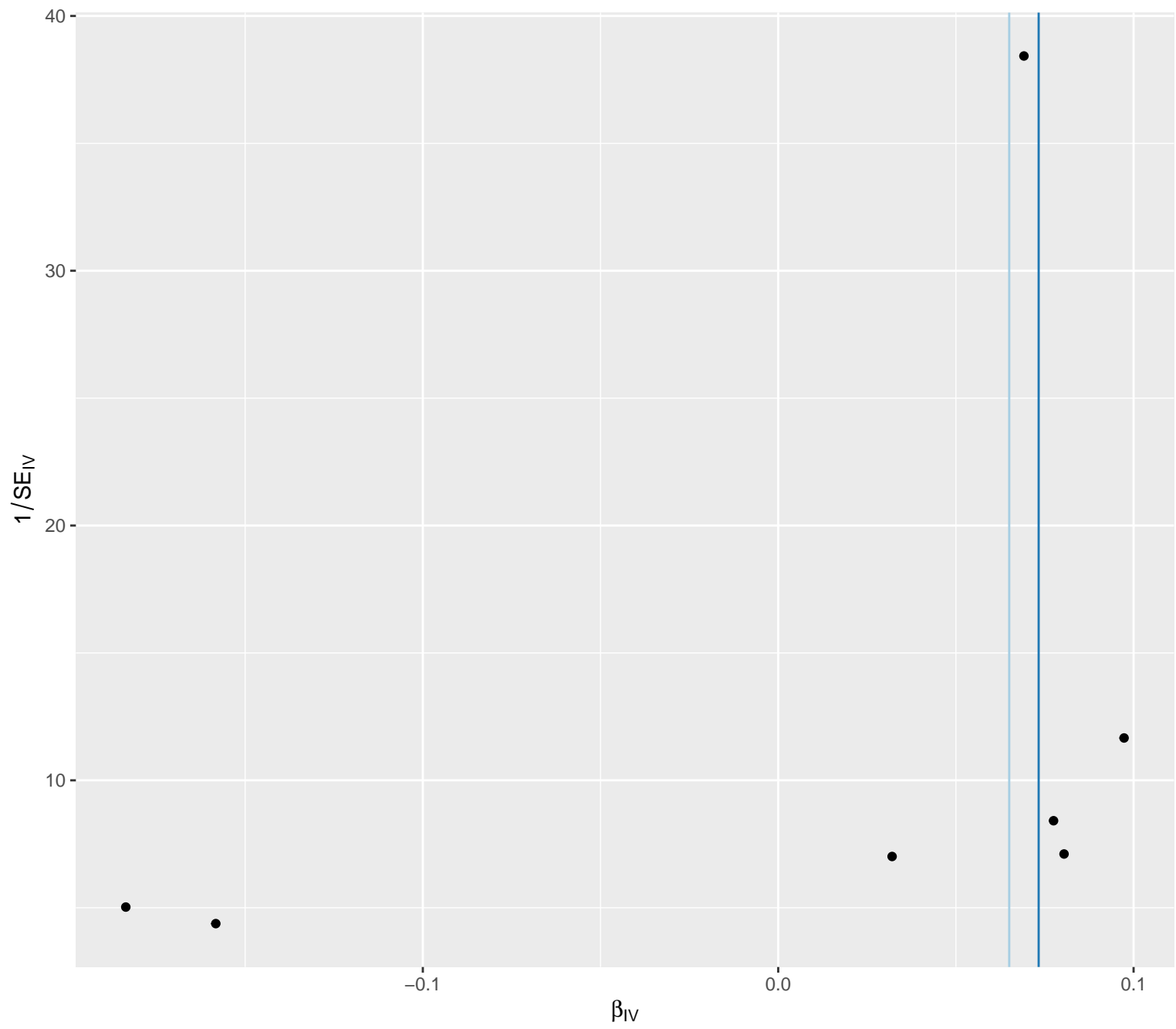

Supplement: S3 File — (ZIP) [file pone.0309088.s003.zip › S3 Fig/ebi-a-GCST90001455/funnelplot.pdf]

# MR Method

- Inverse variance weighted
- MR Egger

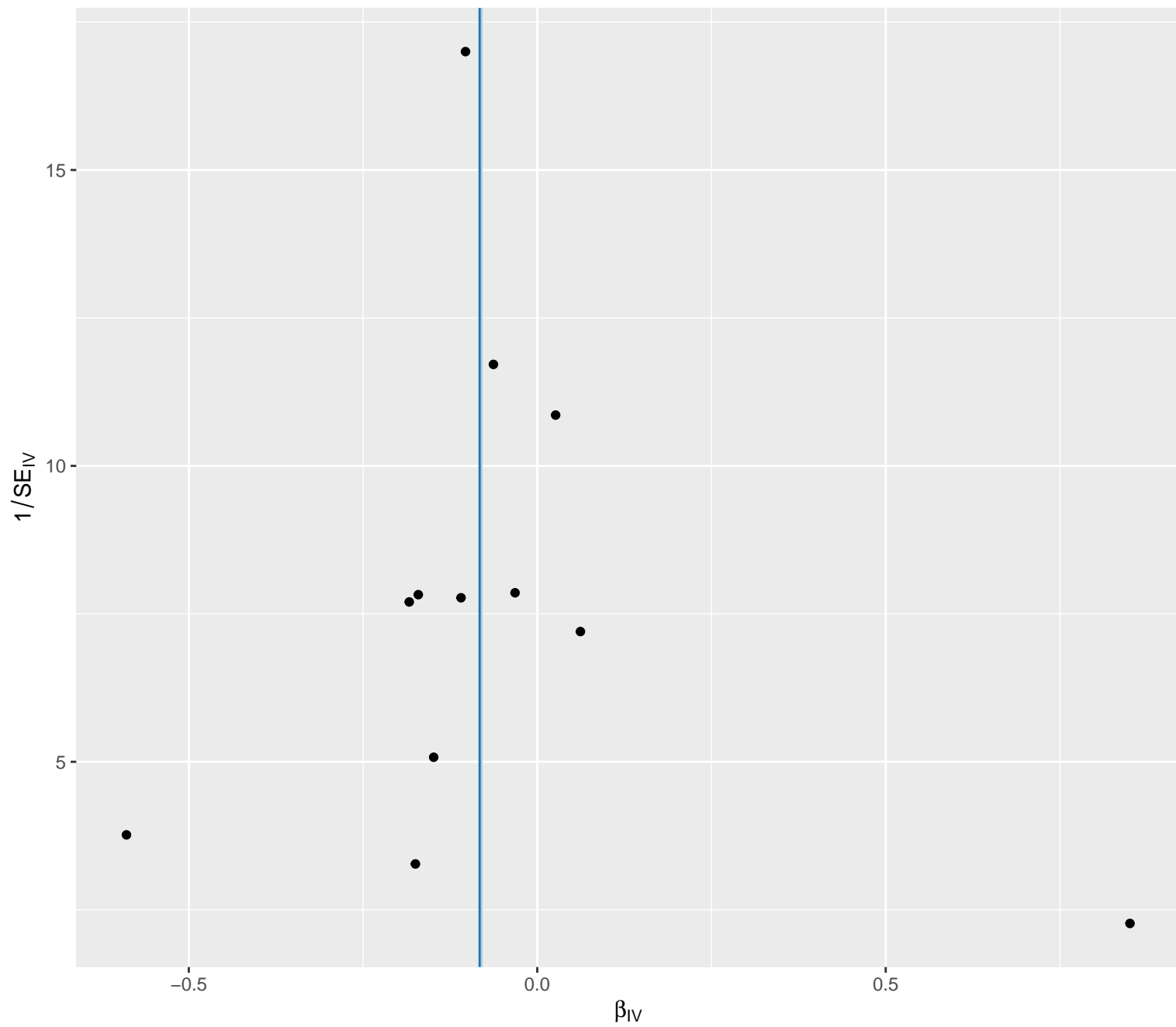

Supplement: S3 File — (ZIP) [file pone.0309088.s003.zip › S3 Fig/ebi-a-GCST90002027/funnelplot.pdf]

# MR Method

- Inverse variance weighted
- MR Egger

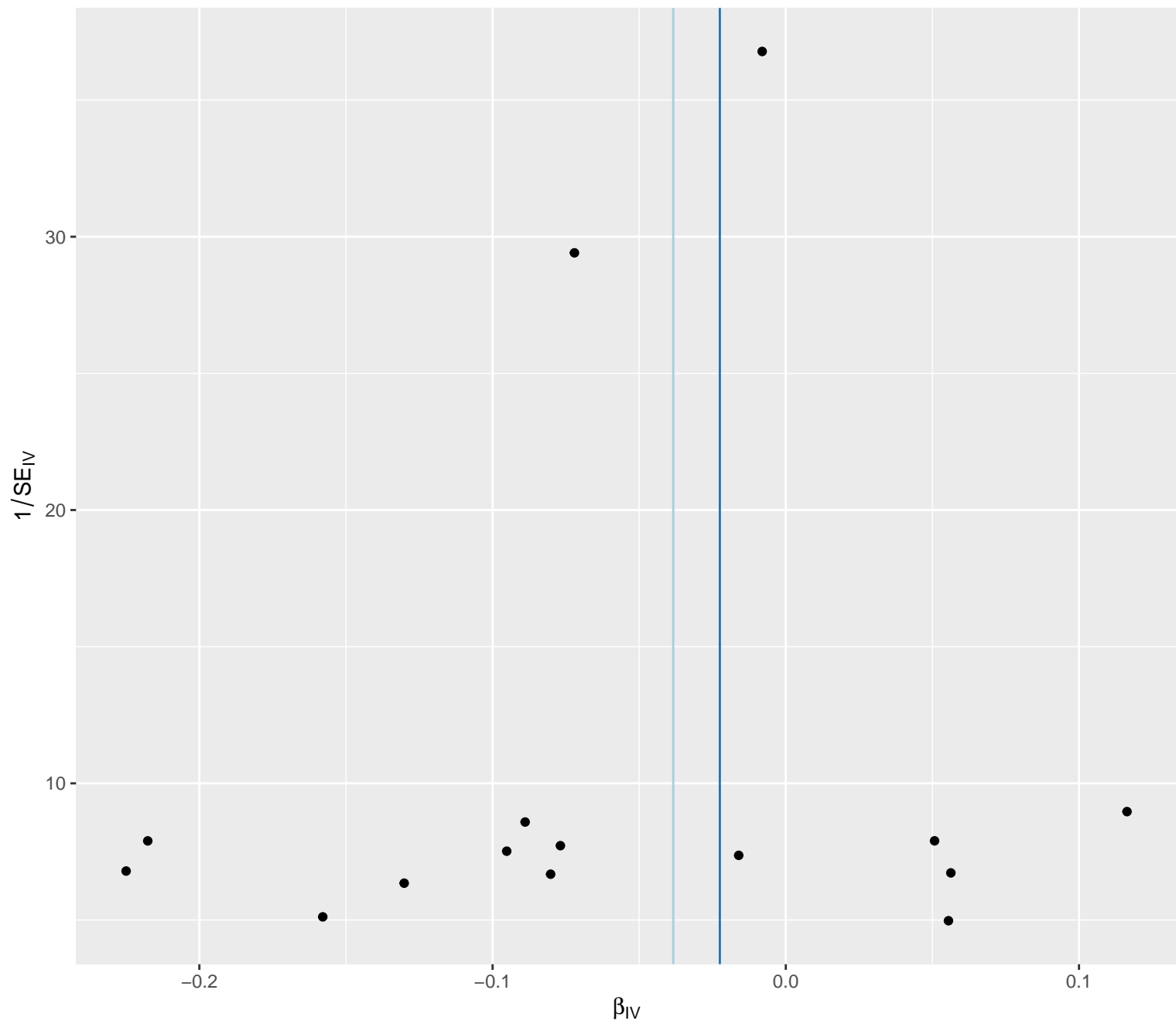

Supplement: S3 File — (ZIP) [file pone.0309088.s003.zip › S3 Fig/ebi-a-GCST90001464/funnelplot.pdf]

# MR Method

- Inverse variance weighted
- MR Egger

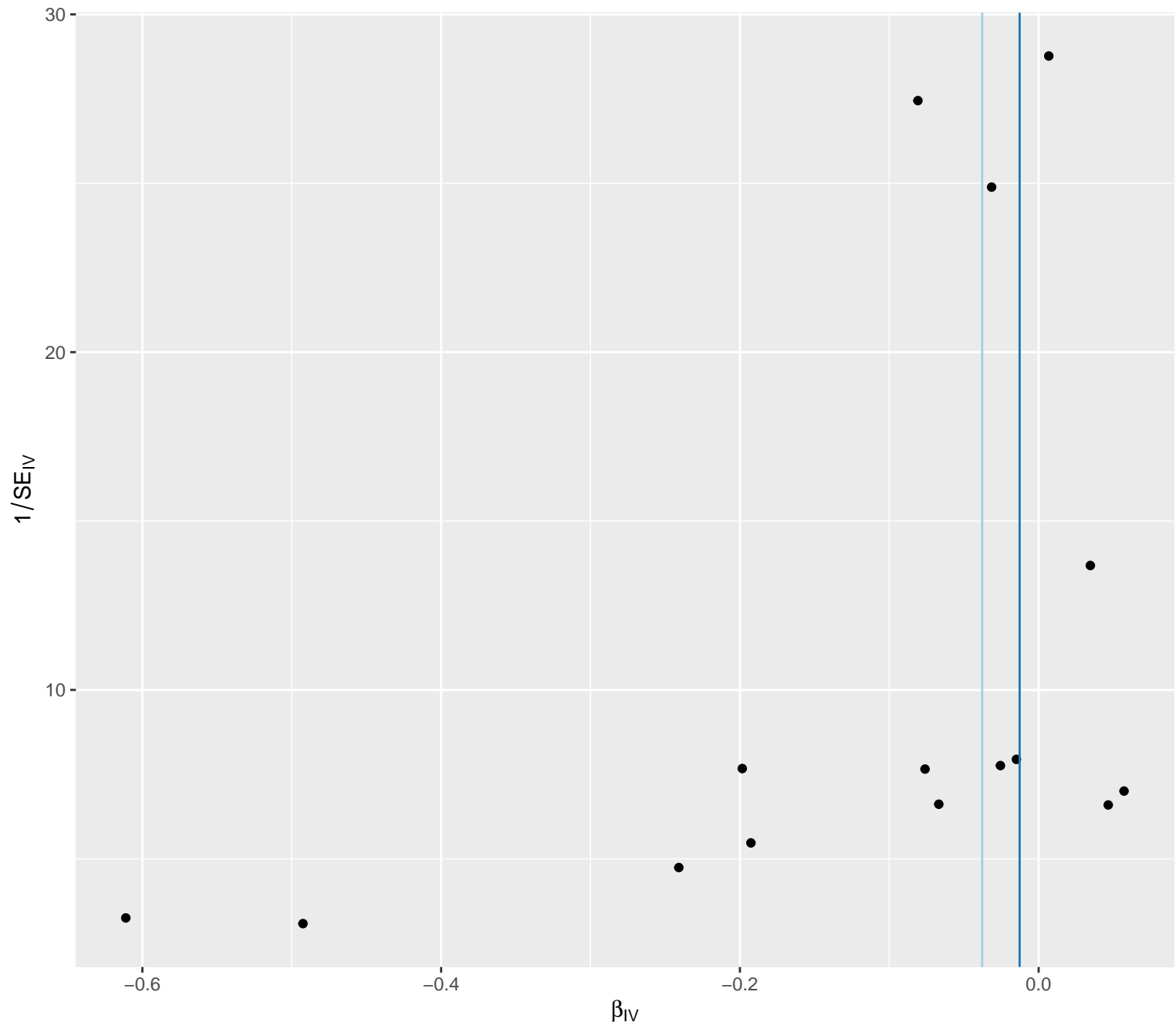

Supplement: S3 File — (ZIP) [file pone.0309088.s003.zip › S3 Fig/ebi-a-GCST90001463/funnelplot.pdf]

# MR Method

- Inverse variance weighted
- MR Egger

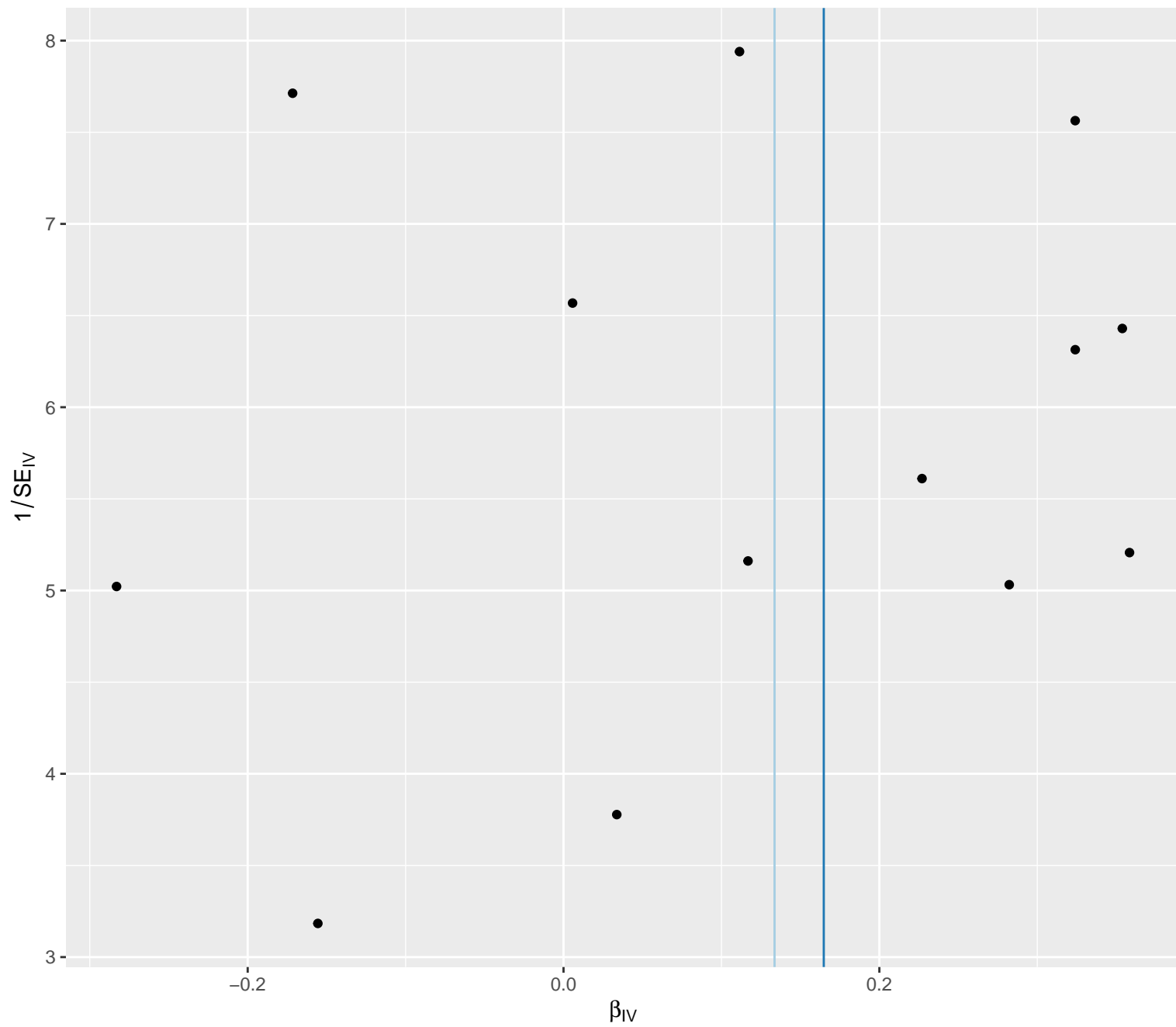

Supplement: S3 File — (ZIP) [file pone.0309088.s003.zip › S3 Fig/ebi-a-GCST90001437/funnelplot.pdf]

# MR Method

- Inverse variance weighted
- MR Egger

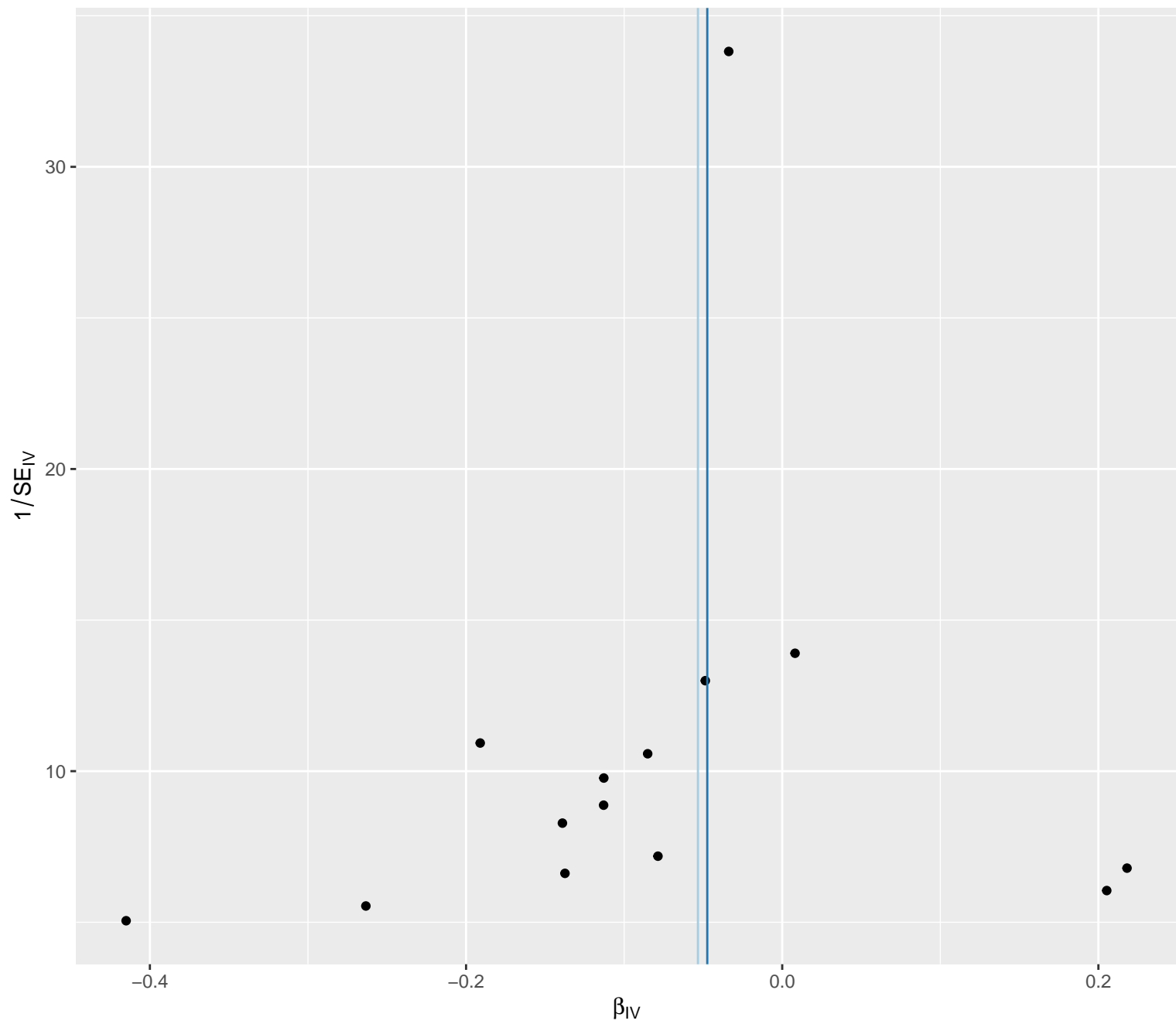

Supplement: S3 File — (ZIP) [file pone.0309088.s003.zip › S3 Fig/ebi-a-GCST90001857/funnelplot.pdf]

# MR Method

- Inverse variance weighted
- MR Egger

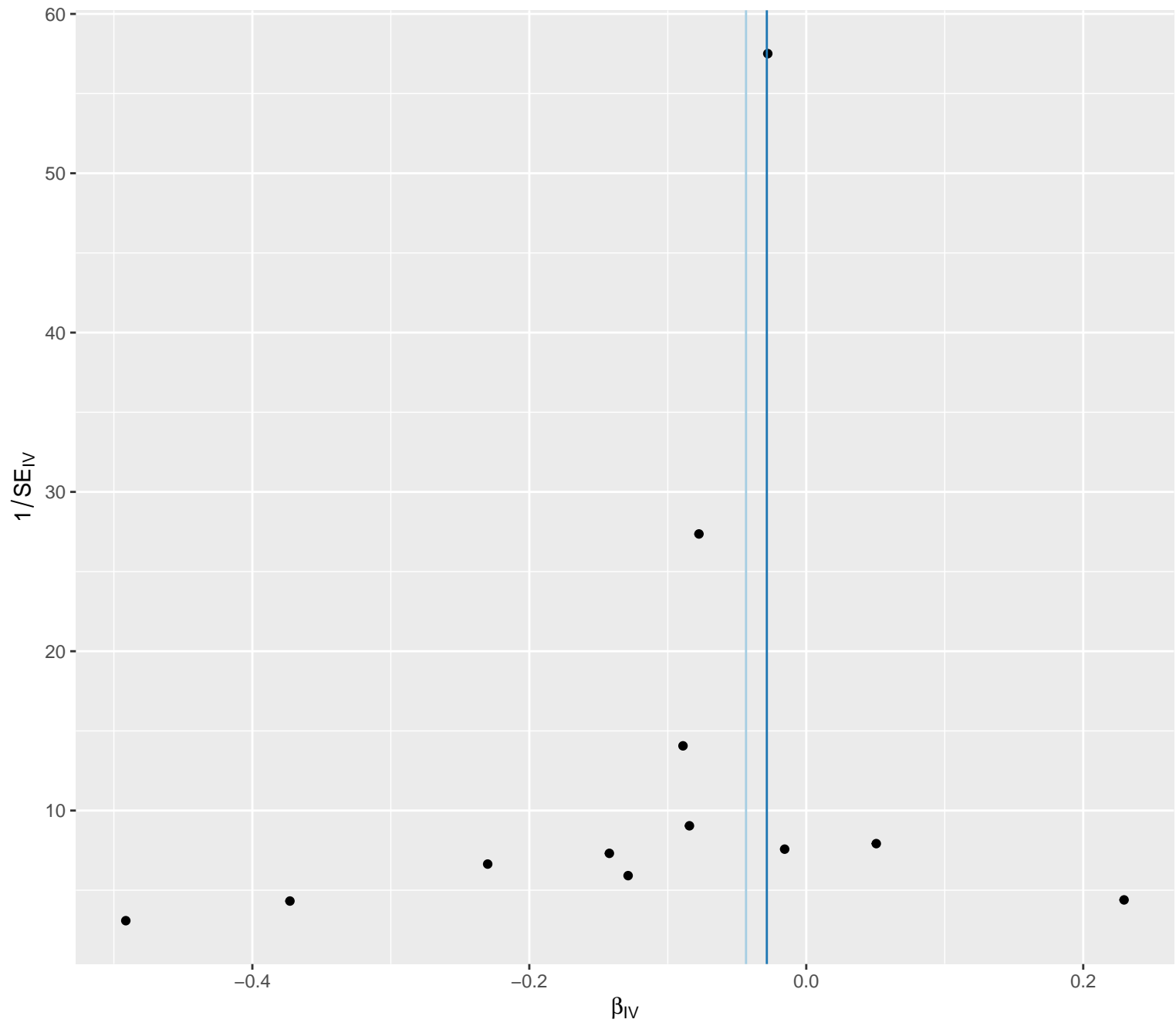

Supplement: S3 File — (ZIP) [file pone.0309088.s003.zip › S3 Fig/ebi-a-GCST90001462/funnelplot.pdf]

# MR Method

- Inverse variance weighted
- MR Egger

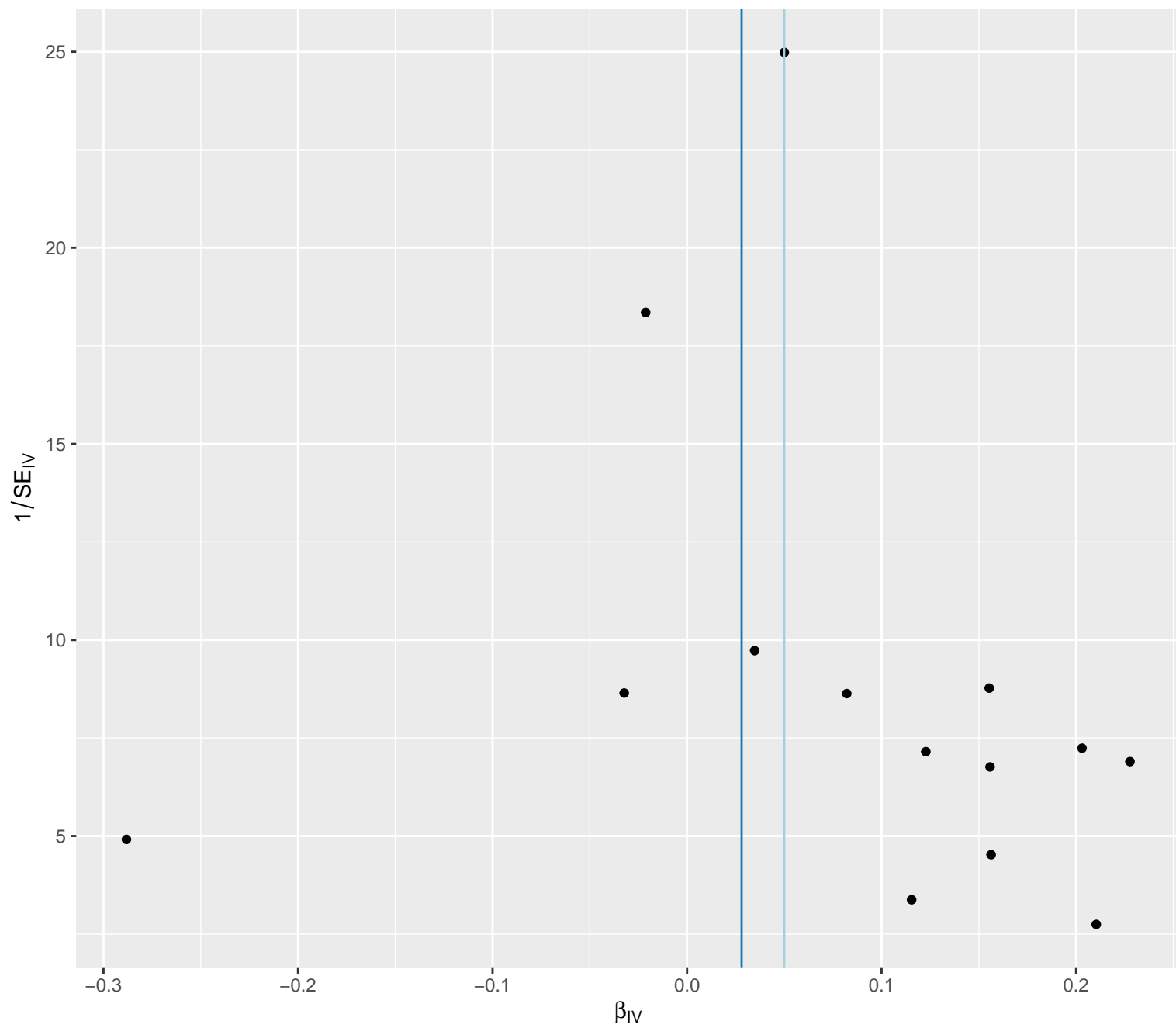

Supplement: S3 File — (ZIP) [file pone.0309088.s003.zip › S3 Fig/ebi-a-GCST90001914/funnelplot.pdf]

# MR Method

- Inverse variance weighted
- MR Egger

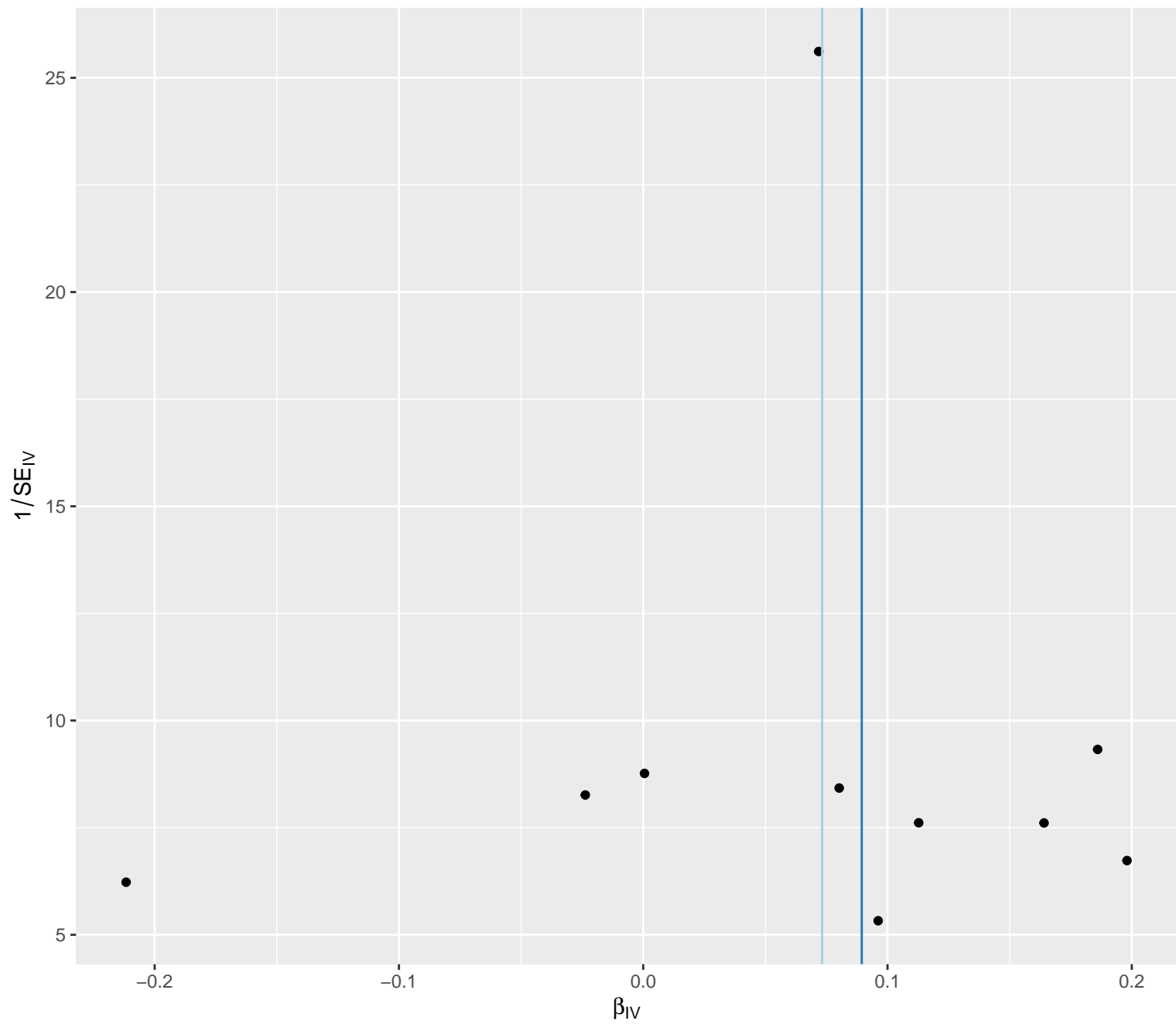

Supplement: S3 File — (ZIP) [file pone.0309088.s003.zip › S3 Fig/ebi-a-GCST90001593/funnelplot.pdf]

# MR Method

- Inverse variance weighted
- MR Egger

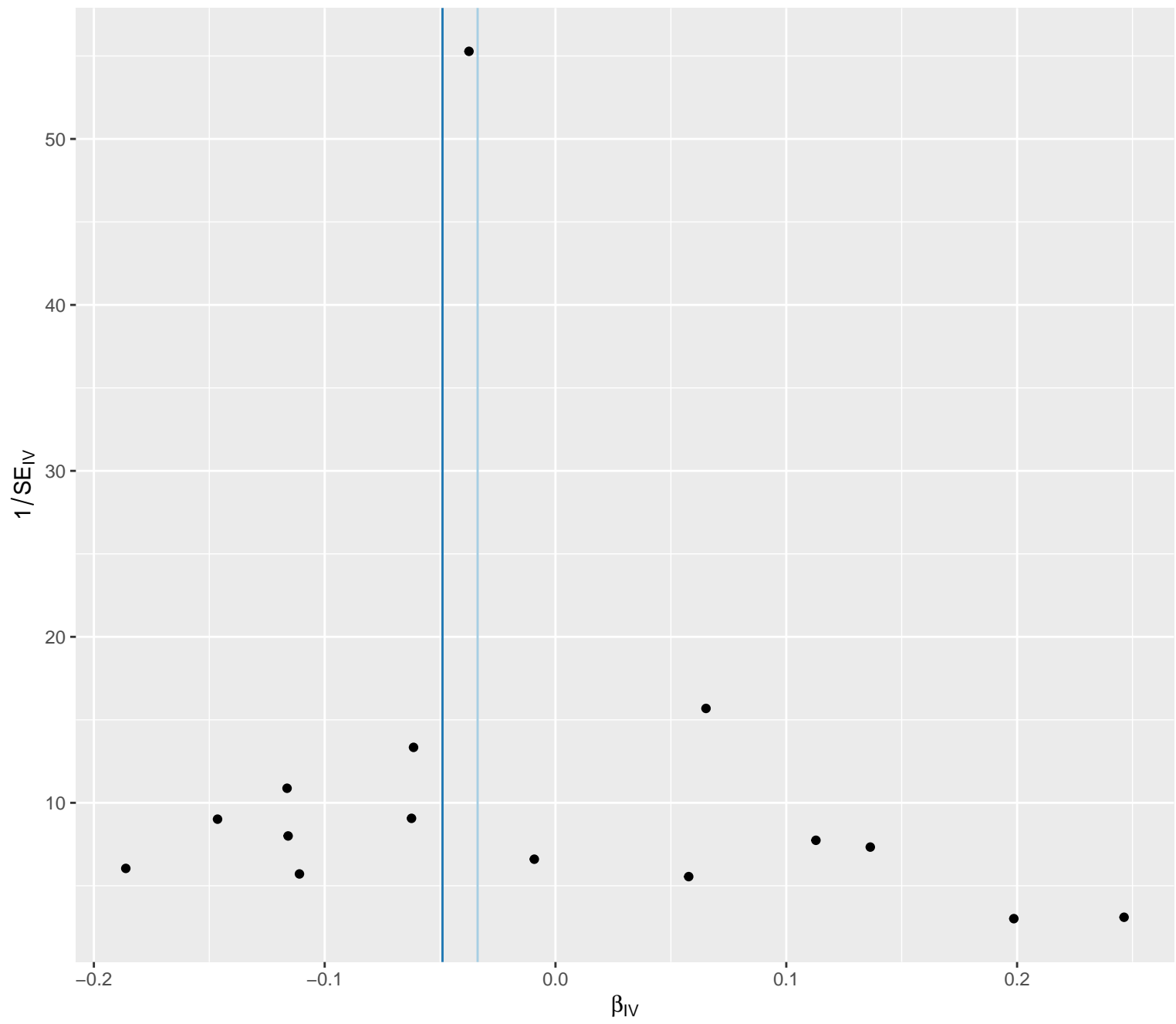

Supplement: S3 File — (ZIP) [file pone.0309088.s003.zip › S3 Fig/ebi-a-GCST90001907/funnelplot.pdf]

# MR Method

- Inverse variance weighted
- MR Egger

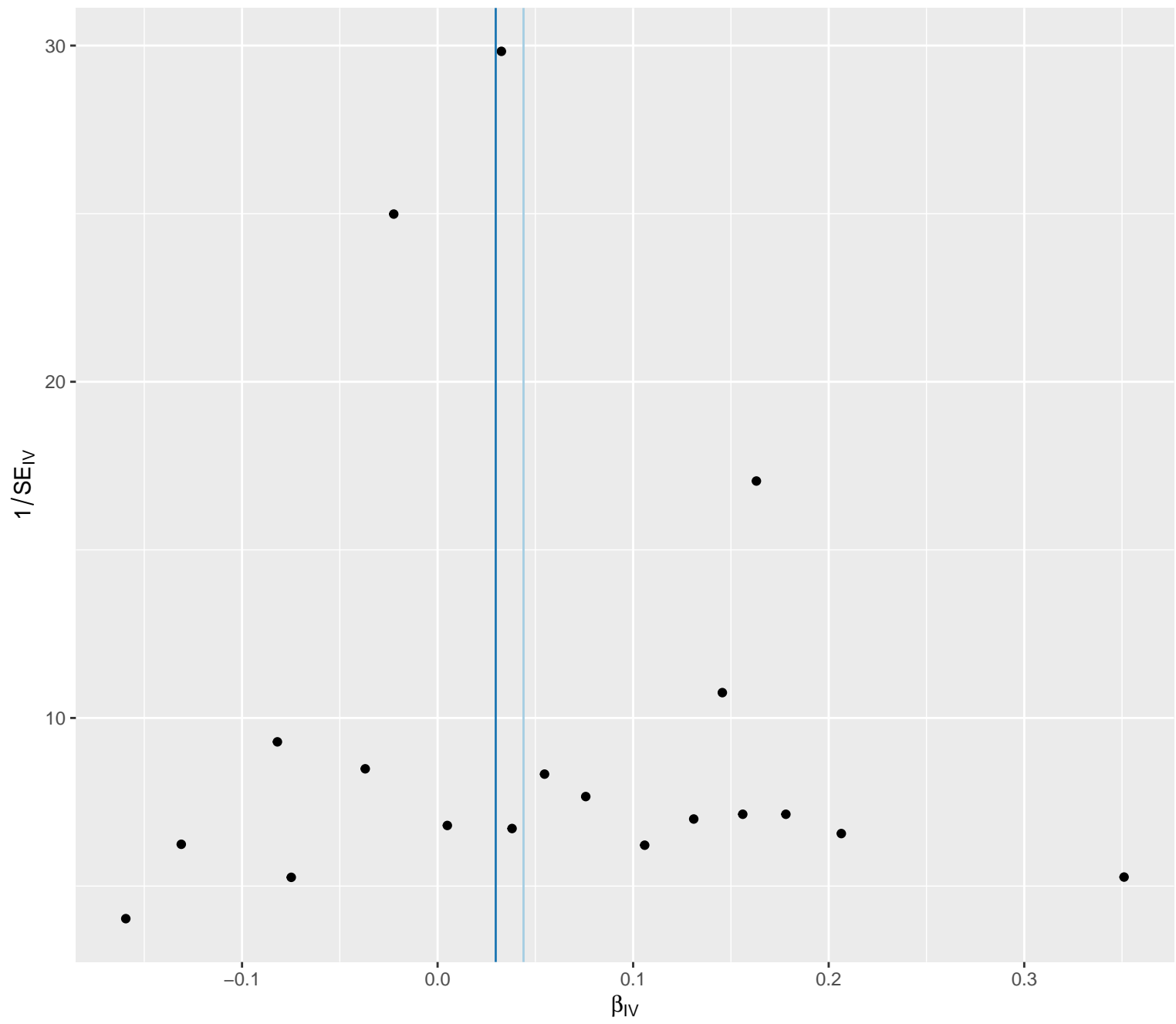

Supplement: S3 File — (ZIP) [file pone.0309088.s003.zip › S3 Fig/ebi-a-GCST90001397/funnelplot.pdf]

# MR Method

- Inverse variance weighted
- MR Egger

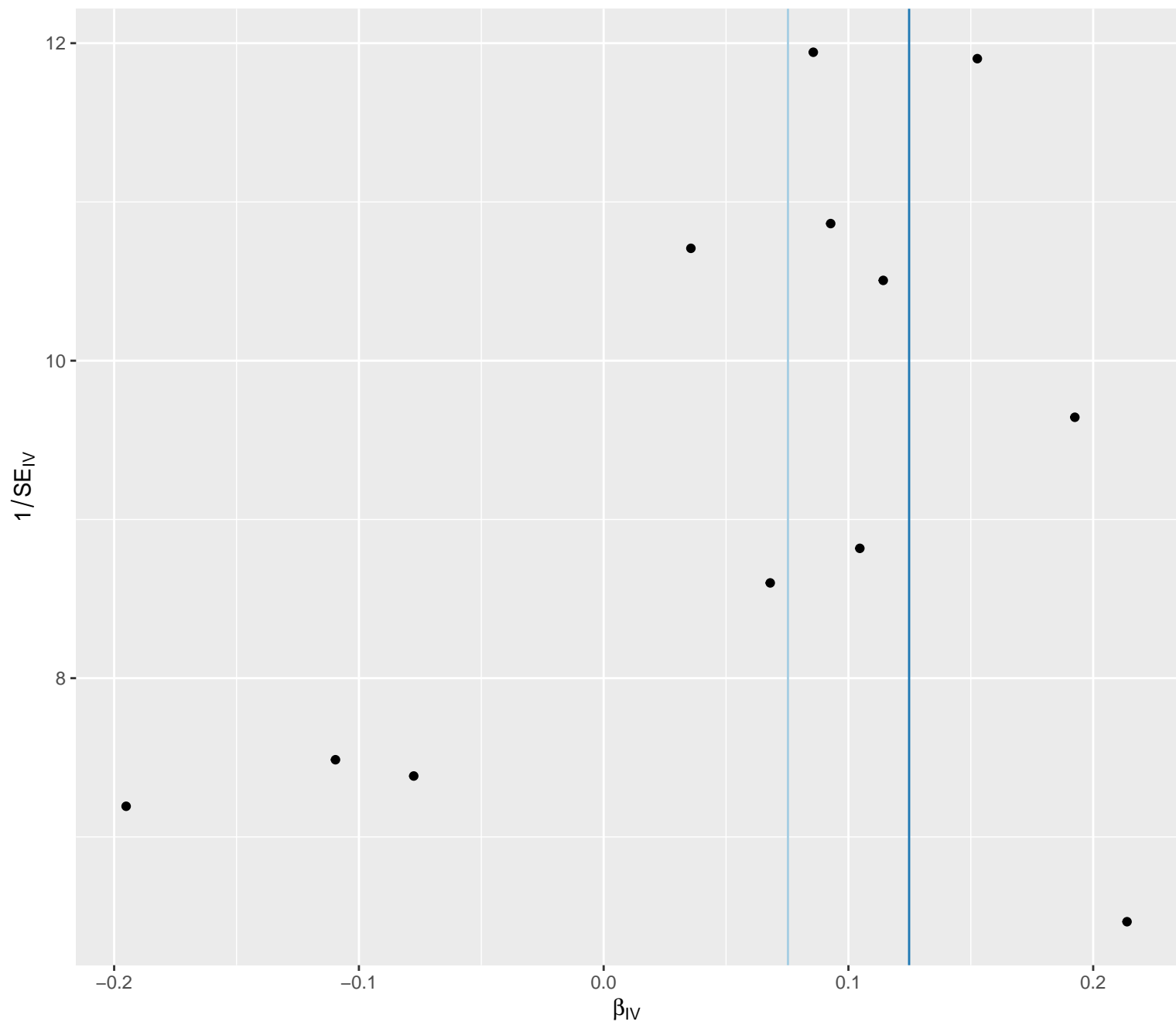

Supplement: S3 File — (ZIP) [file pone.0309088.s003.zip › S3 Fig/ebi-a-GCST90001908/funnelplot.pdf]

# MR Method

- Inverse variance weighted
- MR Egger

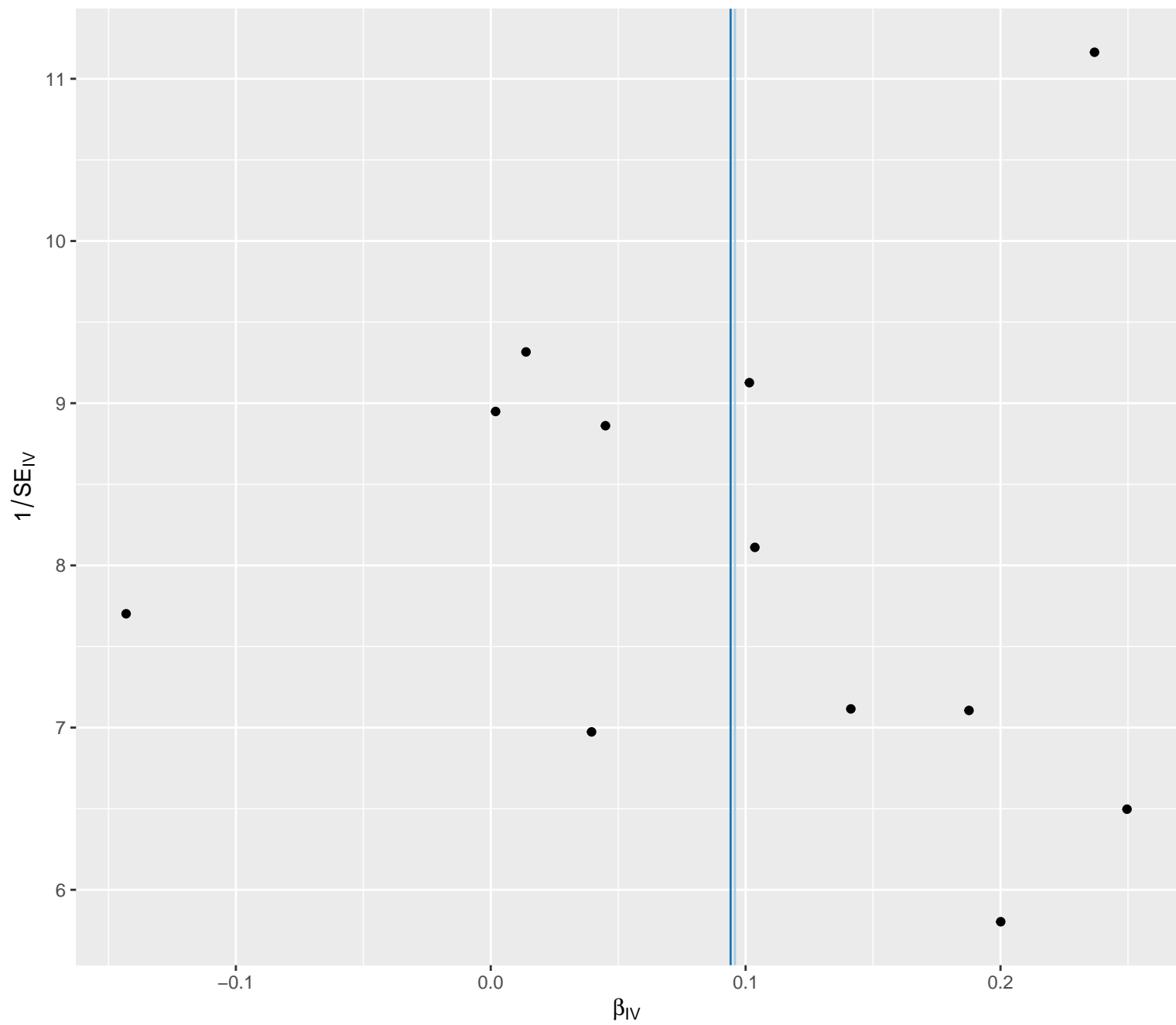

Supplement: S3 File — (ZIP) [file pone.0309088.s003.zip › S3 Fig/ebi-a-GCST90001906/funnelplot.pdf]

# MR Method

- Inverse variance weighted
- MR Egger

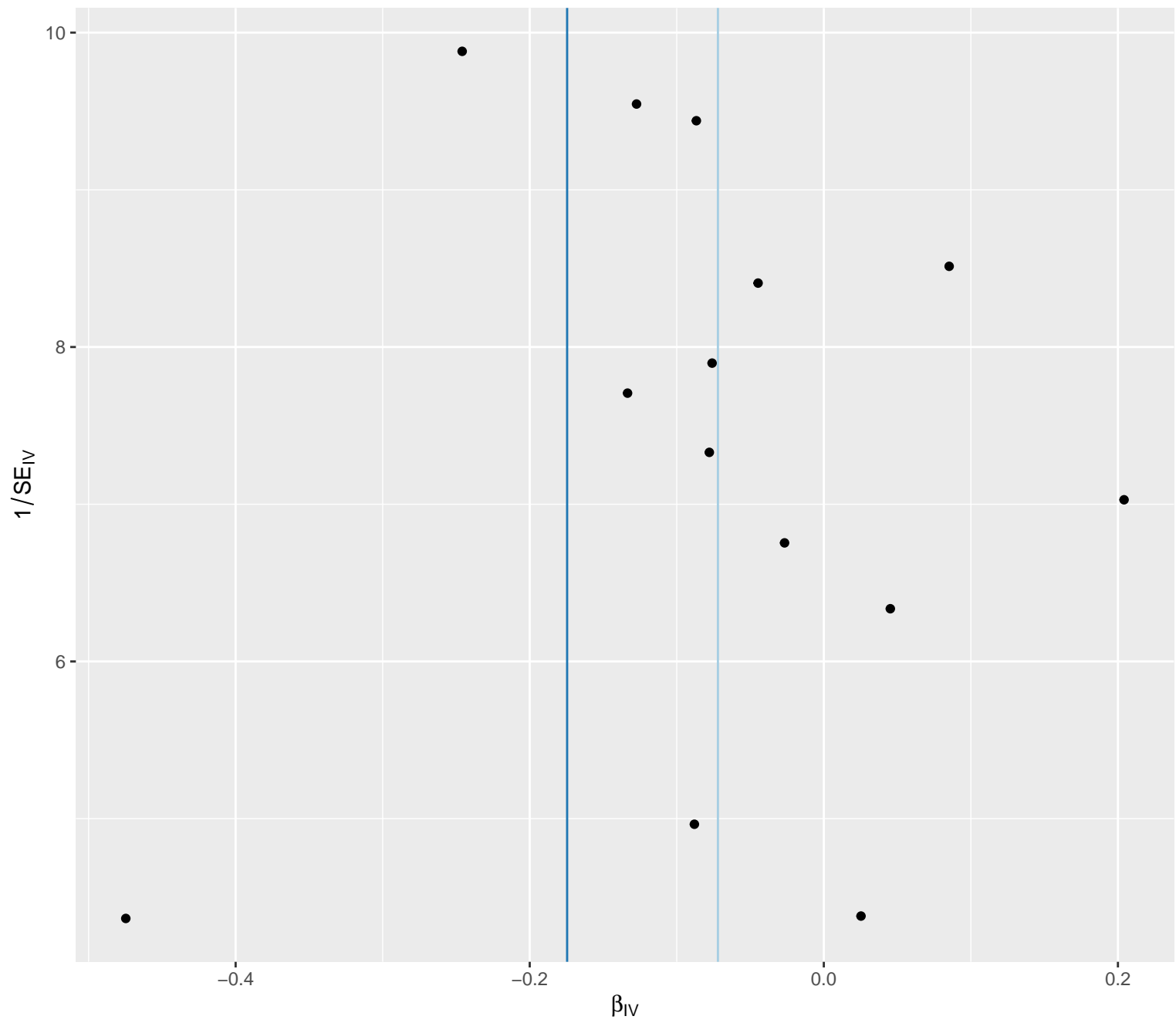

Supplement: S3 File — (ZIP) [file pone.0309088.s003.zip › S3 Fig/ebi-a-GCST90001754/funnelplot.pdf]

# MR Method

- Inverse variance weighted
- MR Egger

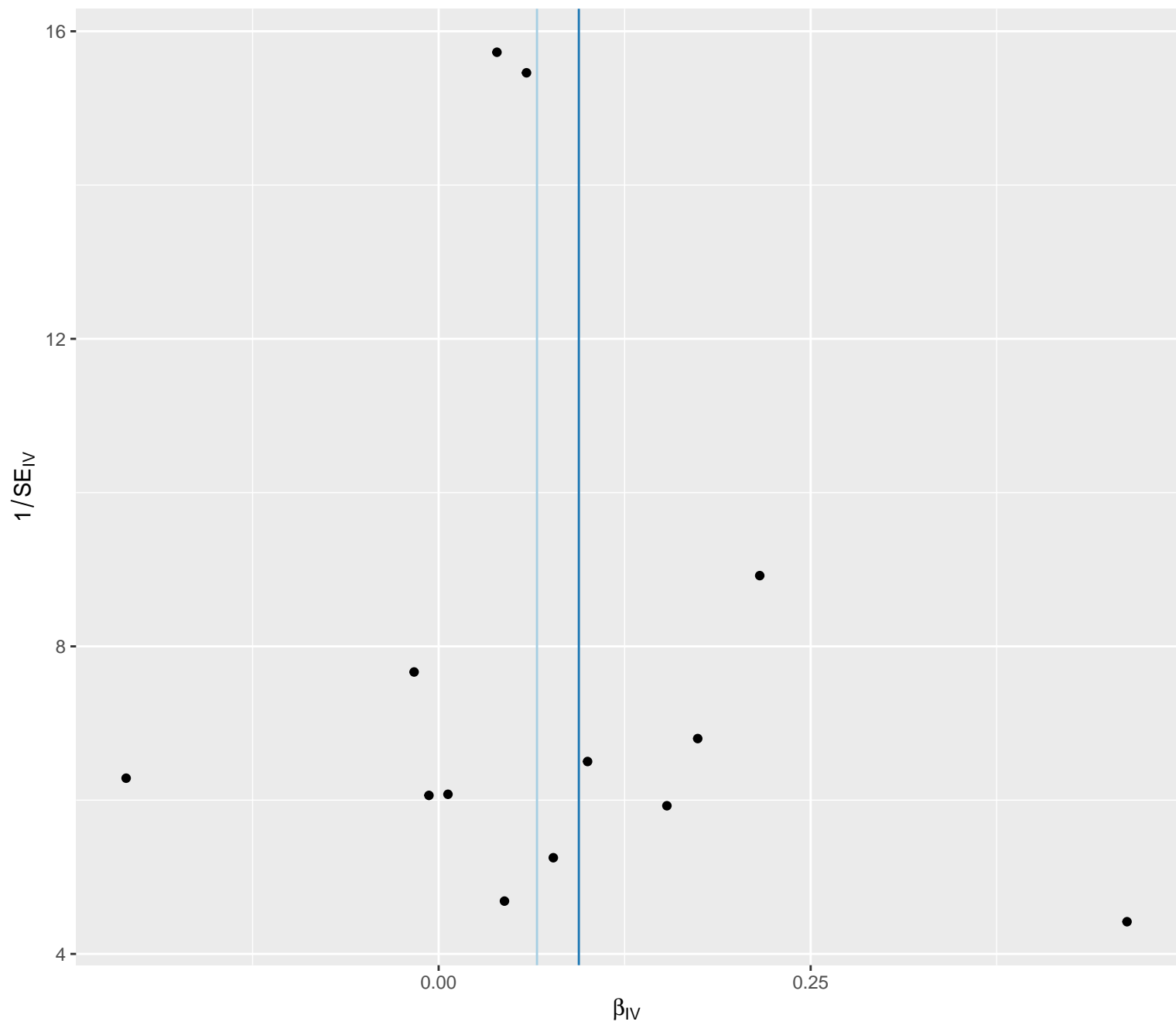

Supplement: S3 File — (ZIP) [file pone.0309088.s003.zip › S3 Fig/ebi-a-GCST90001626/funnelplot.pdf]

# MR Method

- Inverse variance weighted
- MR Egger

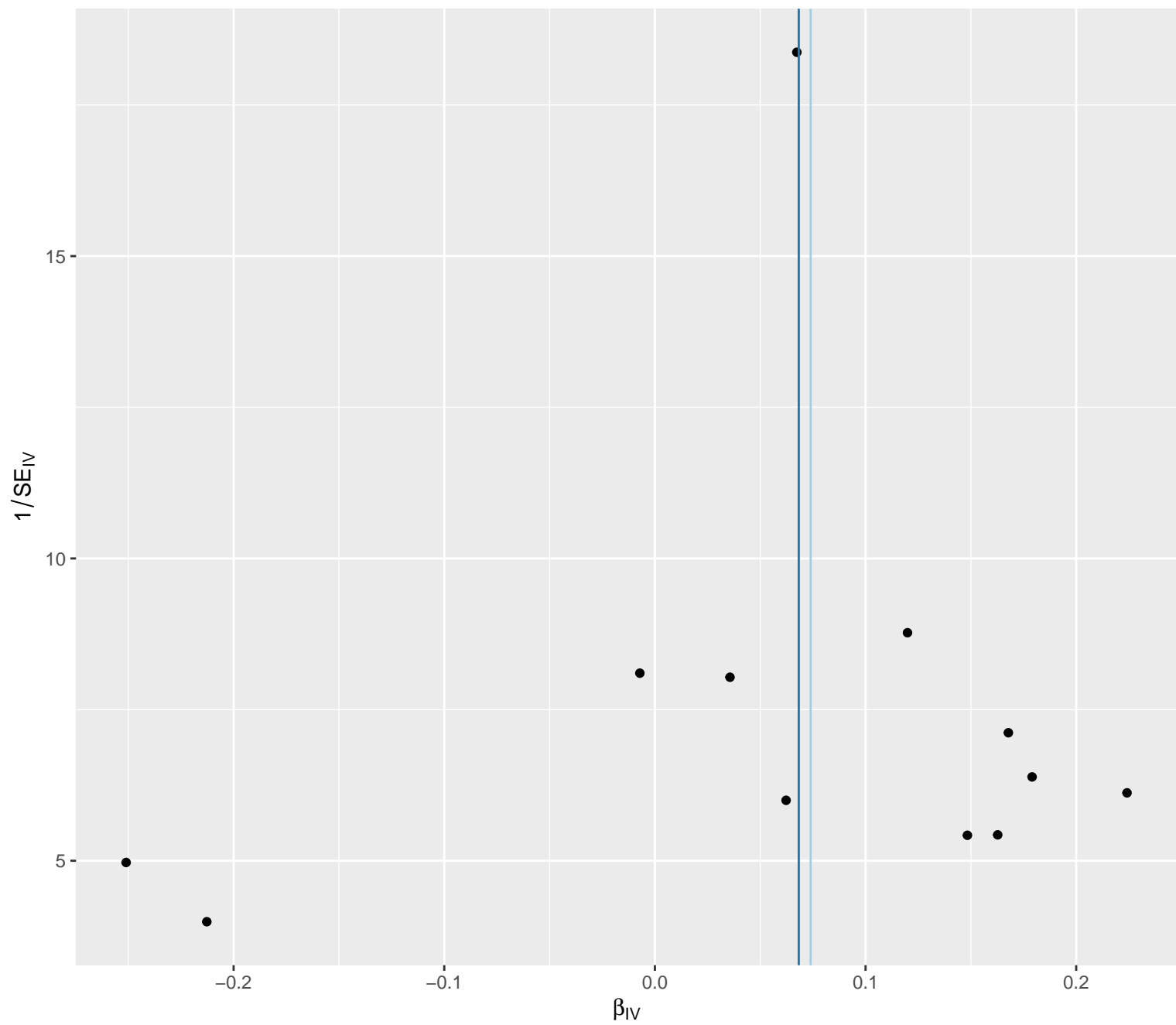

Supplement: S3 File — (ZIP) [file pone.0309088.s003.zip › S3 Fig/ebi-a-GCST90001610/funnelplot.pdf]

# MR Method

- Inverse variance weighted
- MR Egger

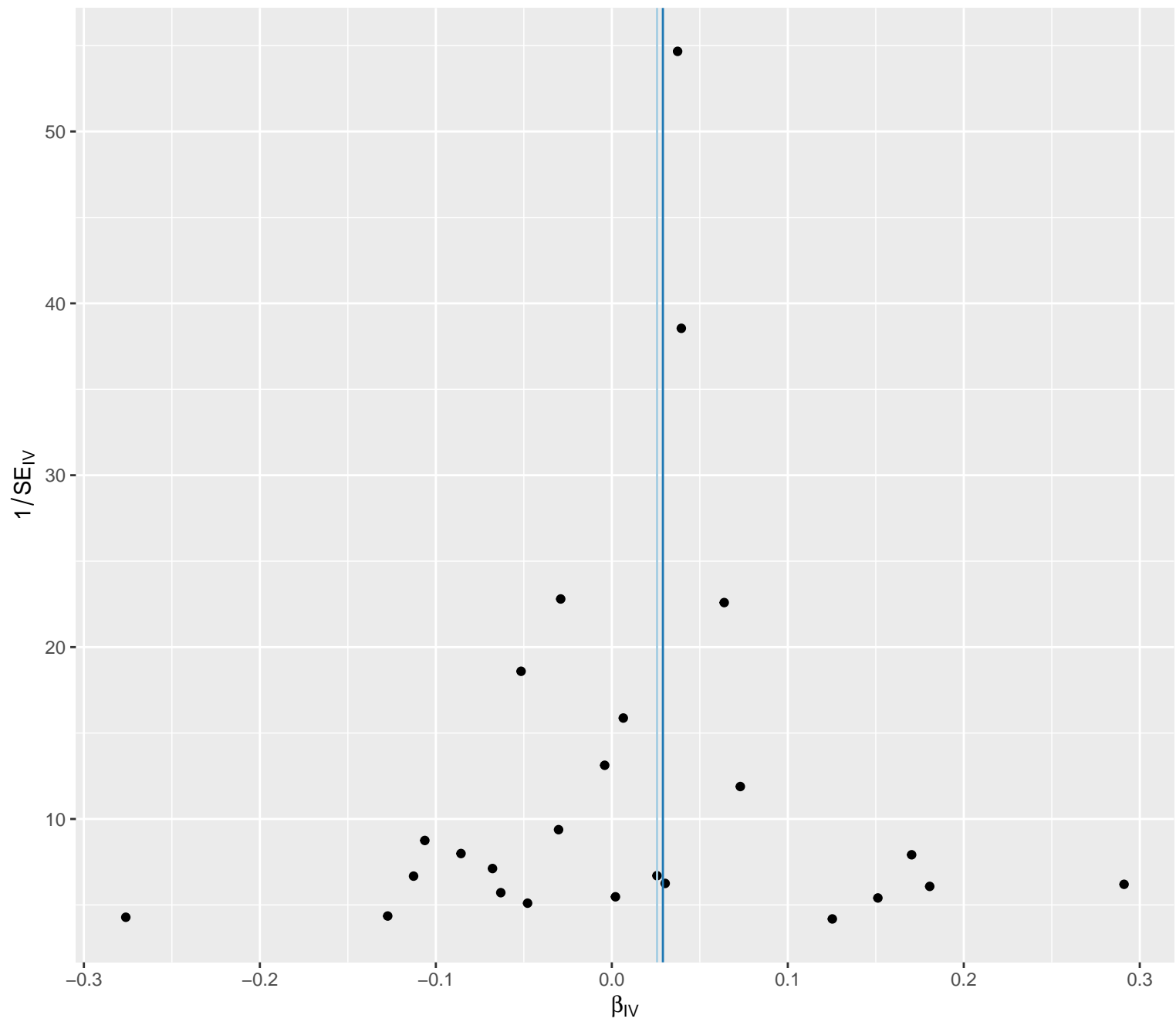

Supplement: S3 File — (ZIP) [file pone.0309088.s003.zip › S3 Fig/ebi-a-GCST90001482/funnelplot.pdf]

# MR Method

- Inverse variance weighted
- MR Egger

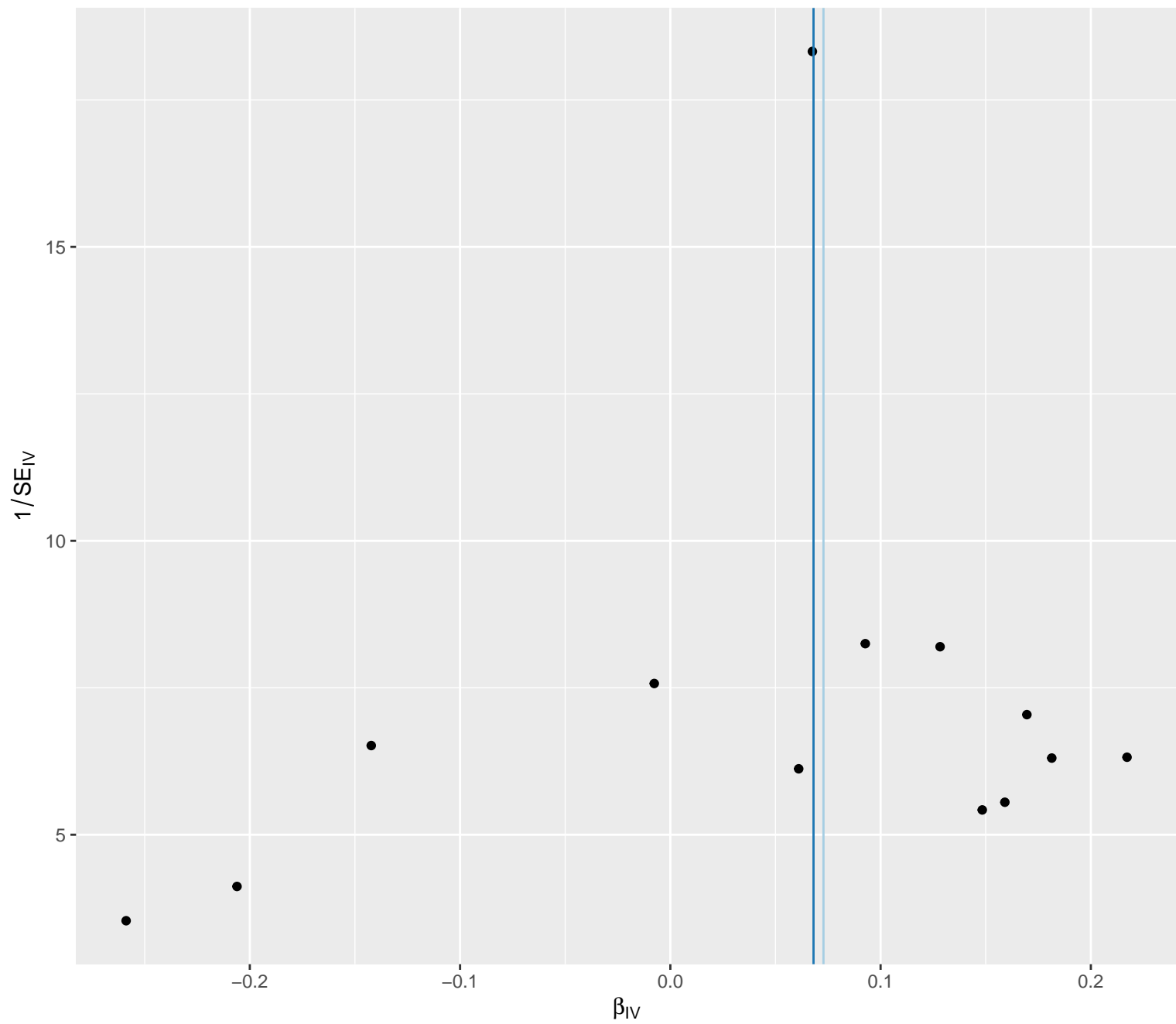

Supplement: S3 File — (ZIP) [file pone.0309088.s003.zip › S3 Fig/ebi-a-GCST90001611/funnelplot.pdf]

# MR Method

- Inverse variance weighted
- MR Egger

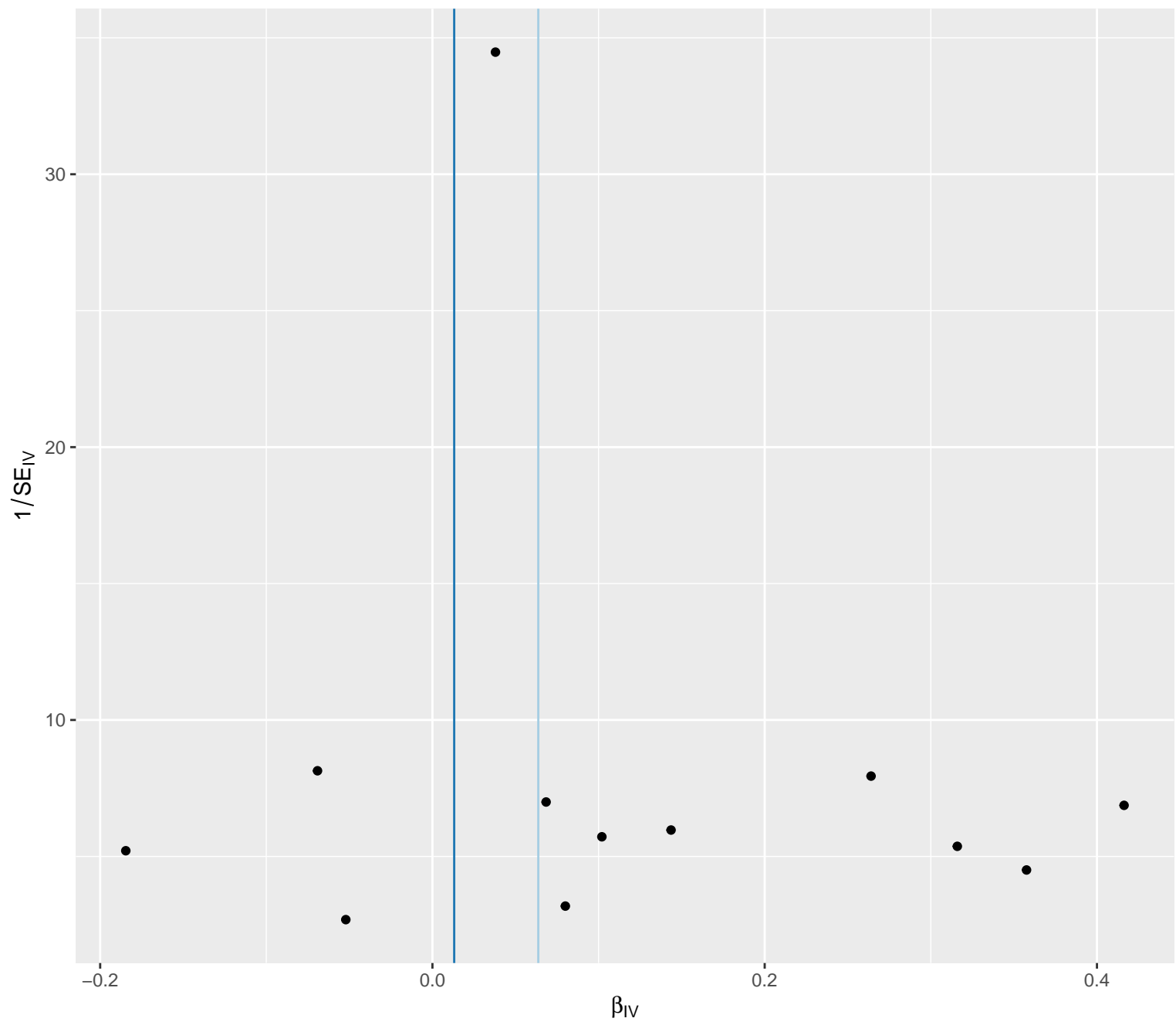

Supplement: S3 File — (ZIP) [file pone.0309088.s003.zip › S3 Fig/ebi-a-GCST90001673/funnelplot.pdf]

# MR Method

- Inverse variance weighted
- MR Egger

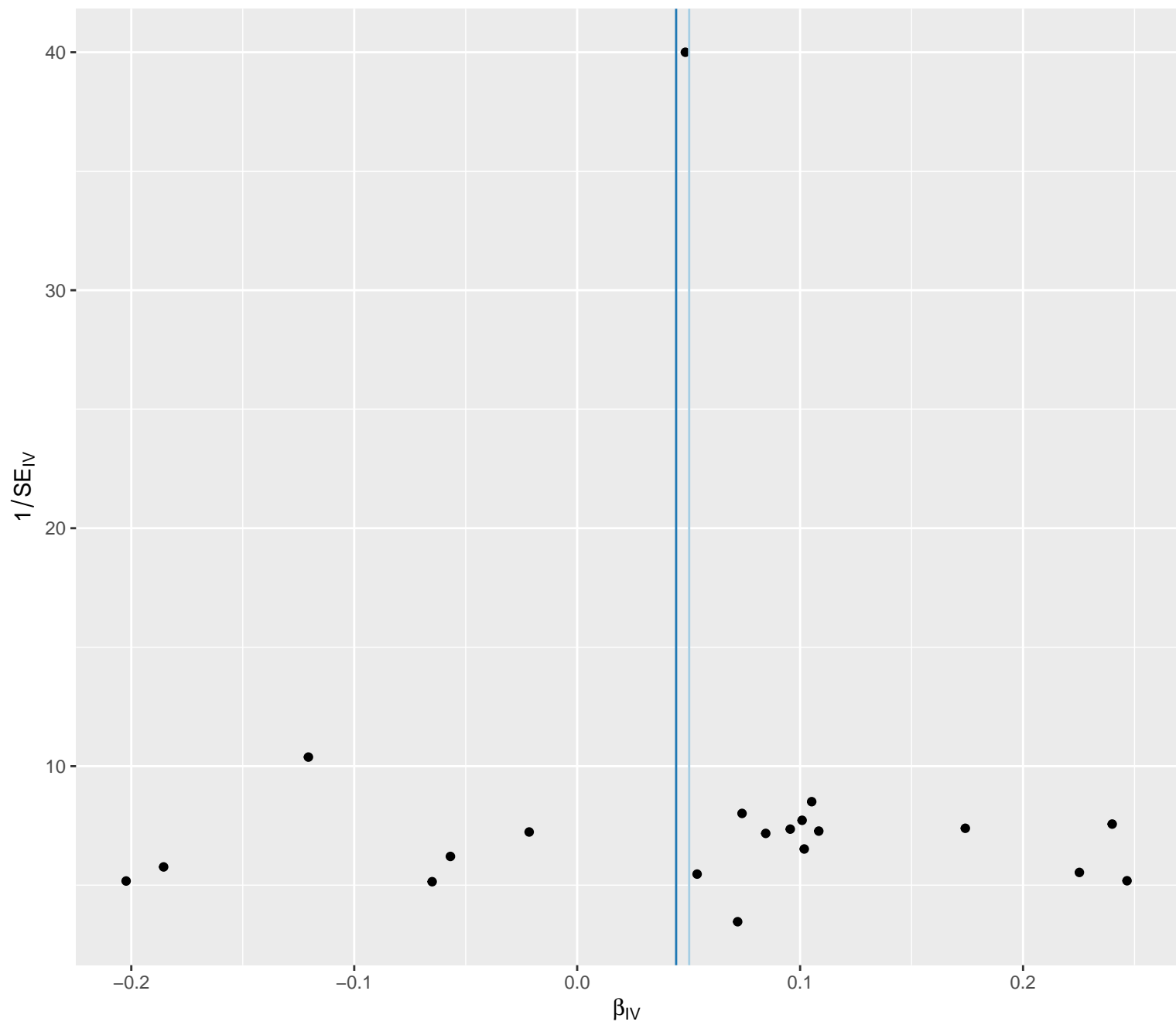

Supplement: S3 File — (ZIP) [file pone.0309088.s003.zip › S3 Fig/ebi-a-GCST90001404/funnelplot.pdf]

# MR Method

- Inverse variance weighted
- MR Egger

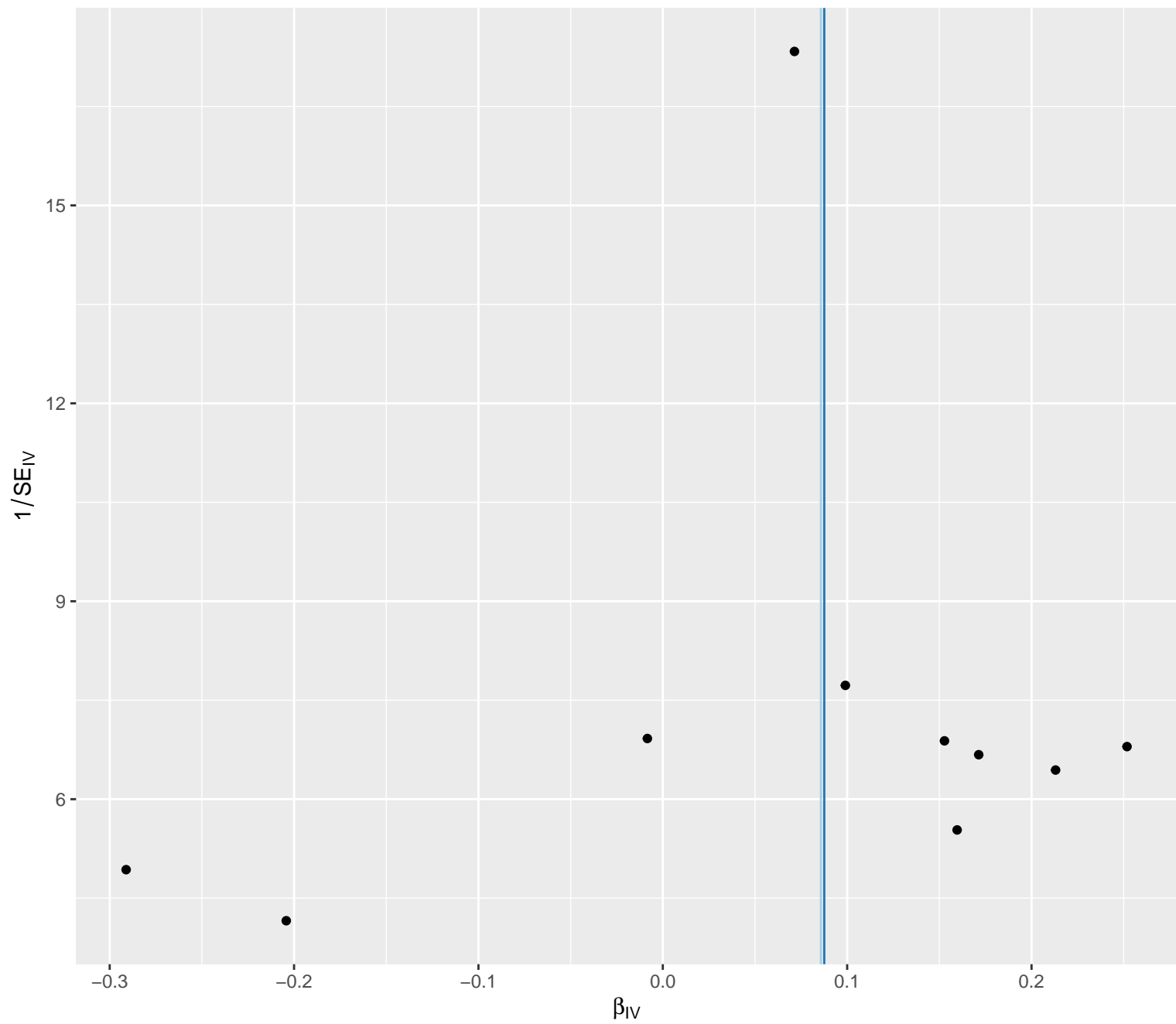

Supplement: S3 File — (ZIP) [file pone.0309088.s003.zip › S3 Fig/ebi-a-GCST90001609/funnelplot.pdf]

# MR Method

- Inverse variance weighted
- MR Egger

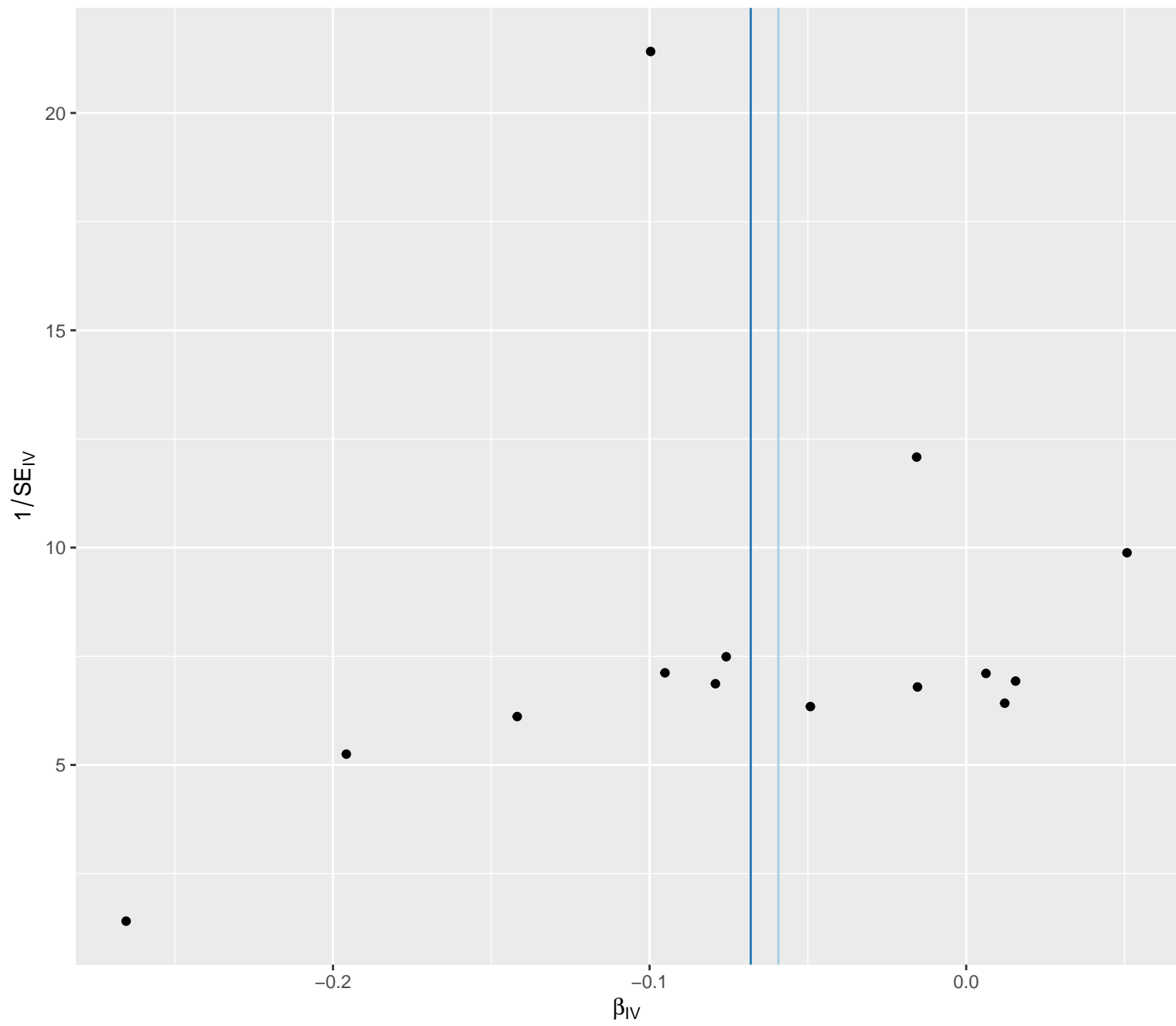

Supplement: S3 File — (ZIP) [file pone.0309088.s003.zip › S3 Fig/ebi-a-GCST90001461/funnelplot.pdf]

# MR Method

- Inverse variance weighted
- MR Egger

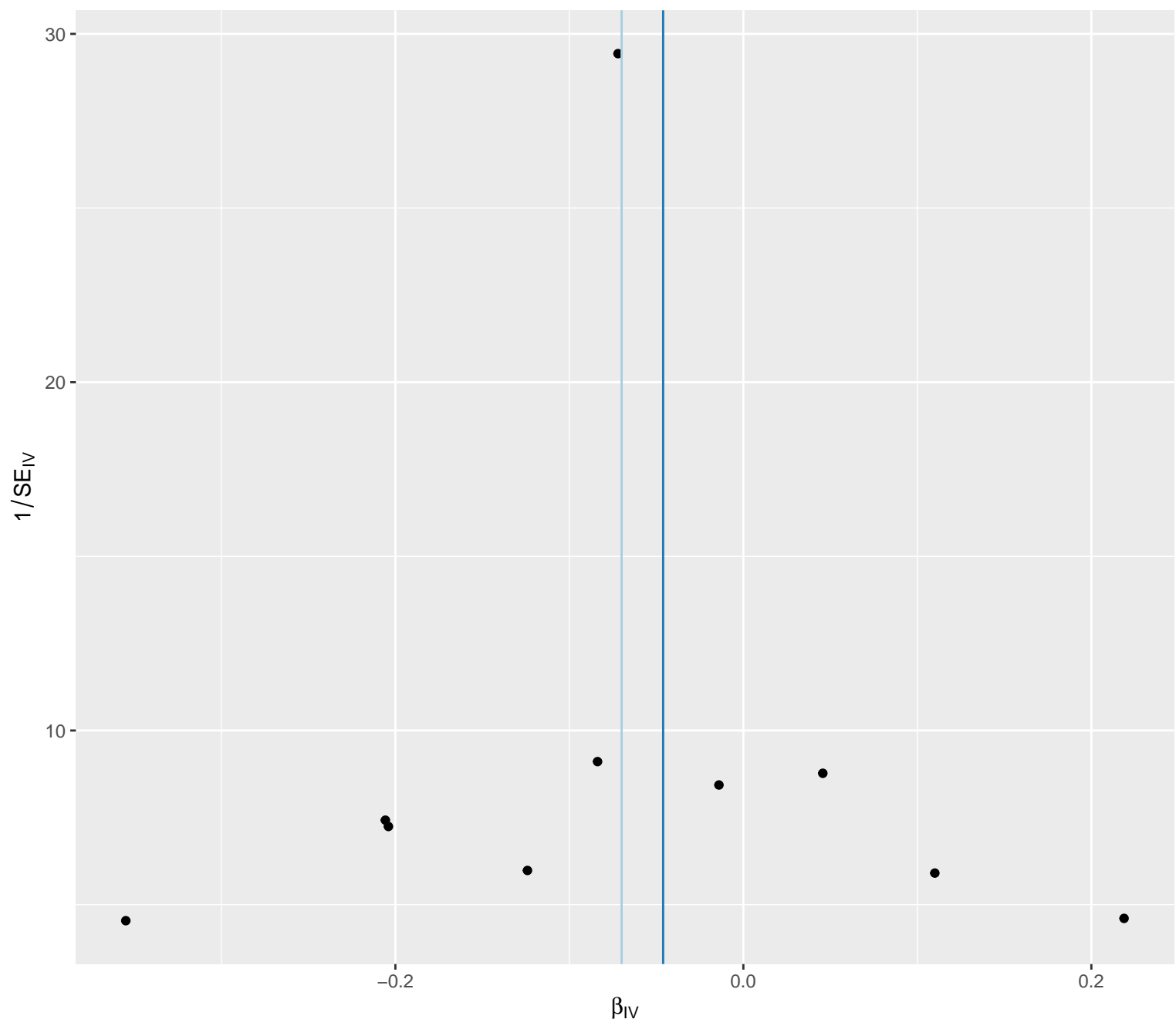

Supplement: S3 File — (ZIP) [file pone.0309088.s003.zip › S3 Fig/ebi-a-GCST90001468/funnelplot.pdf]

# MR Method

- Inverse variance weighted
- MR Egger

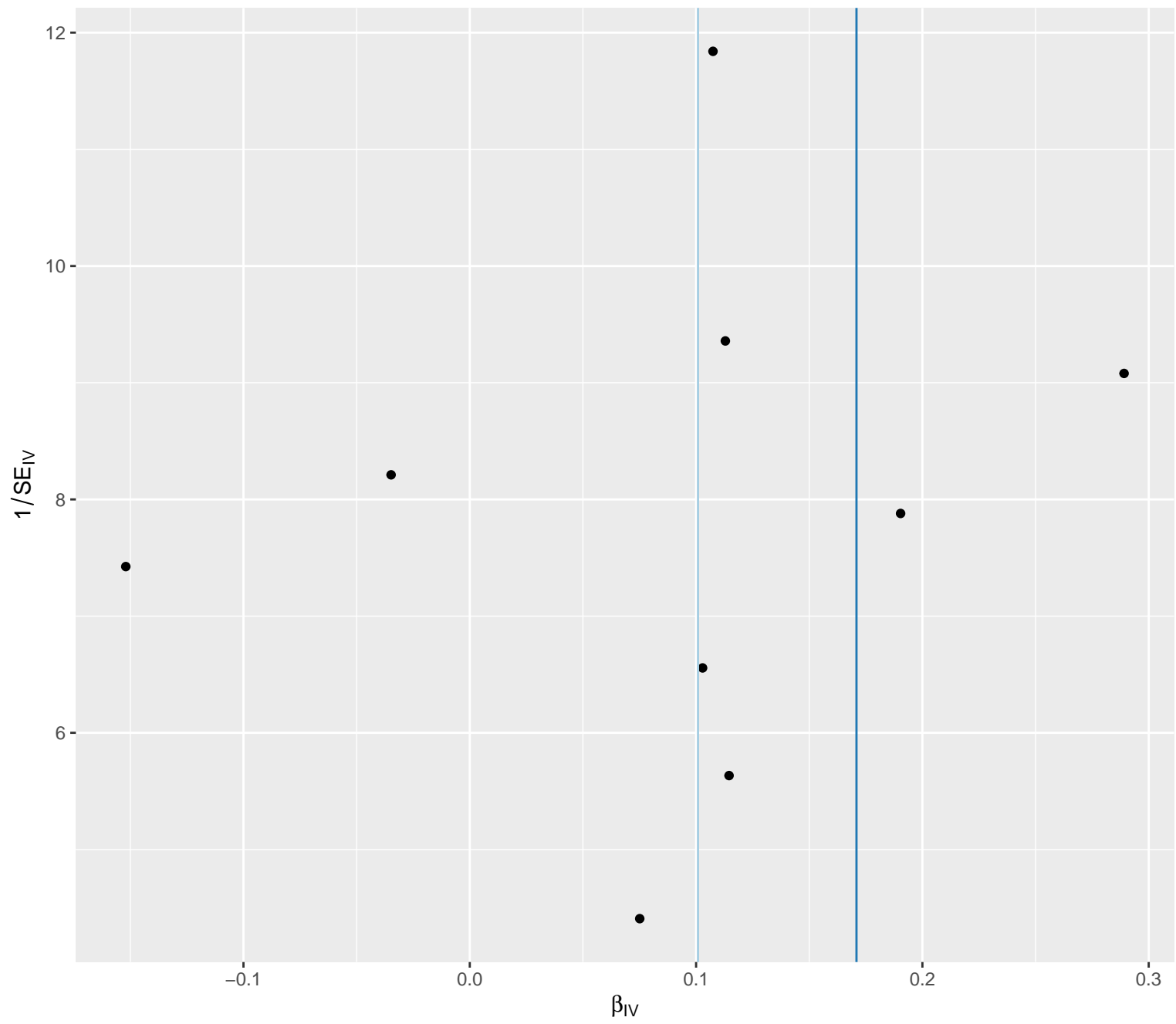

Supplement: S3 File — (ZIP) [file pone.0309088.s003.zip › S3 Fig/ebi-a-GCST90001929/funnelplot.pdf]

# MR Method

- Inverse variance weighted
- MR Egger

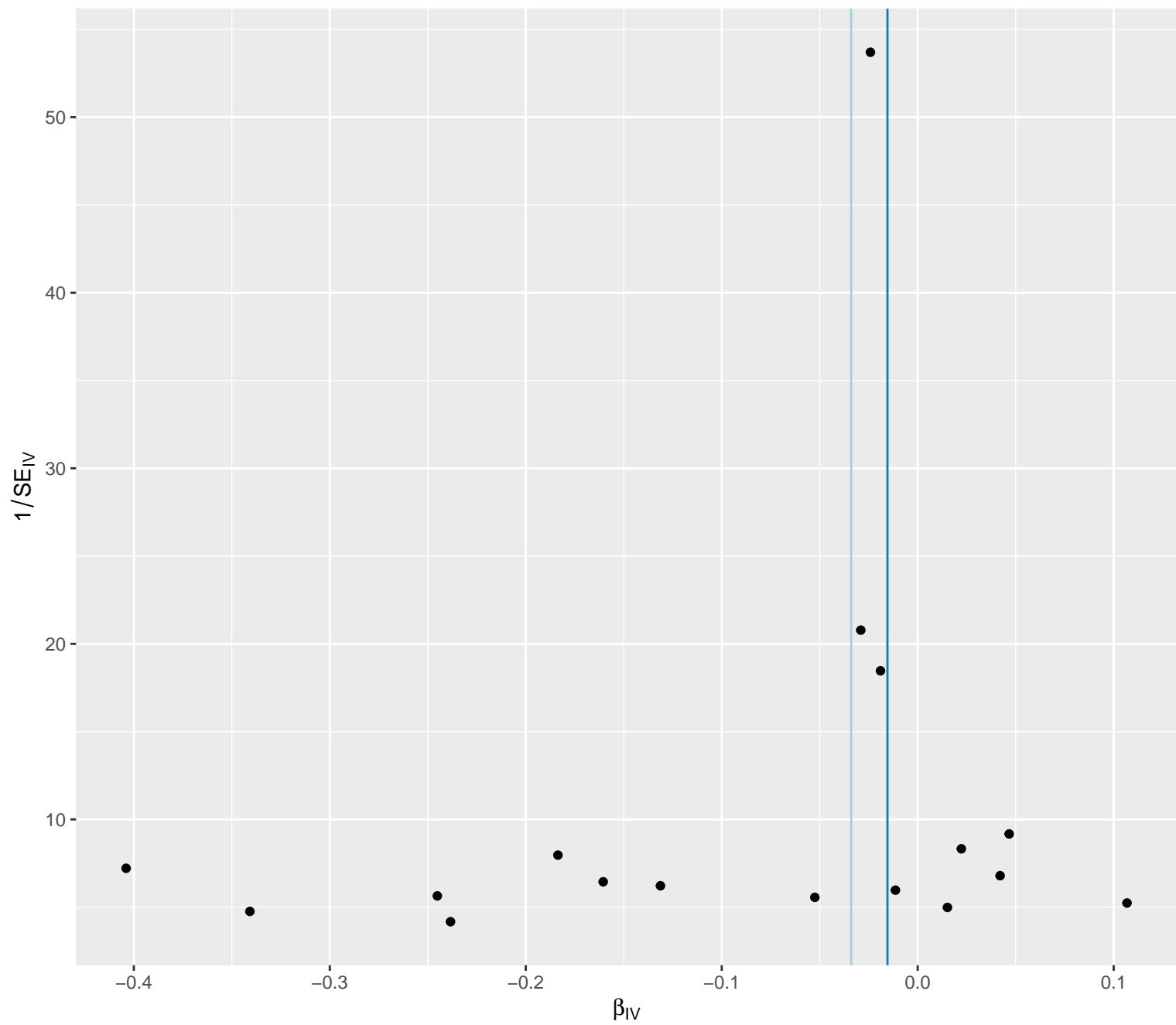

Supplement: S3 File — (ZIP) [file pone.0309088.s003.zip › S3 Fig/ebi-a-GCST90001583/funnelplot.pdf]

# MR Method

- Inverse variance weighted
- MR Egger

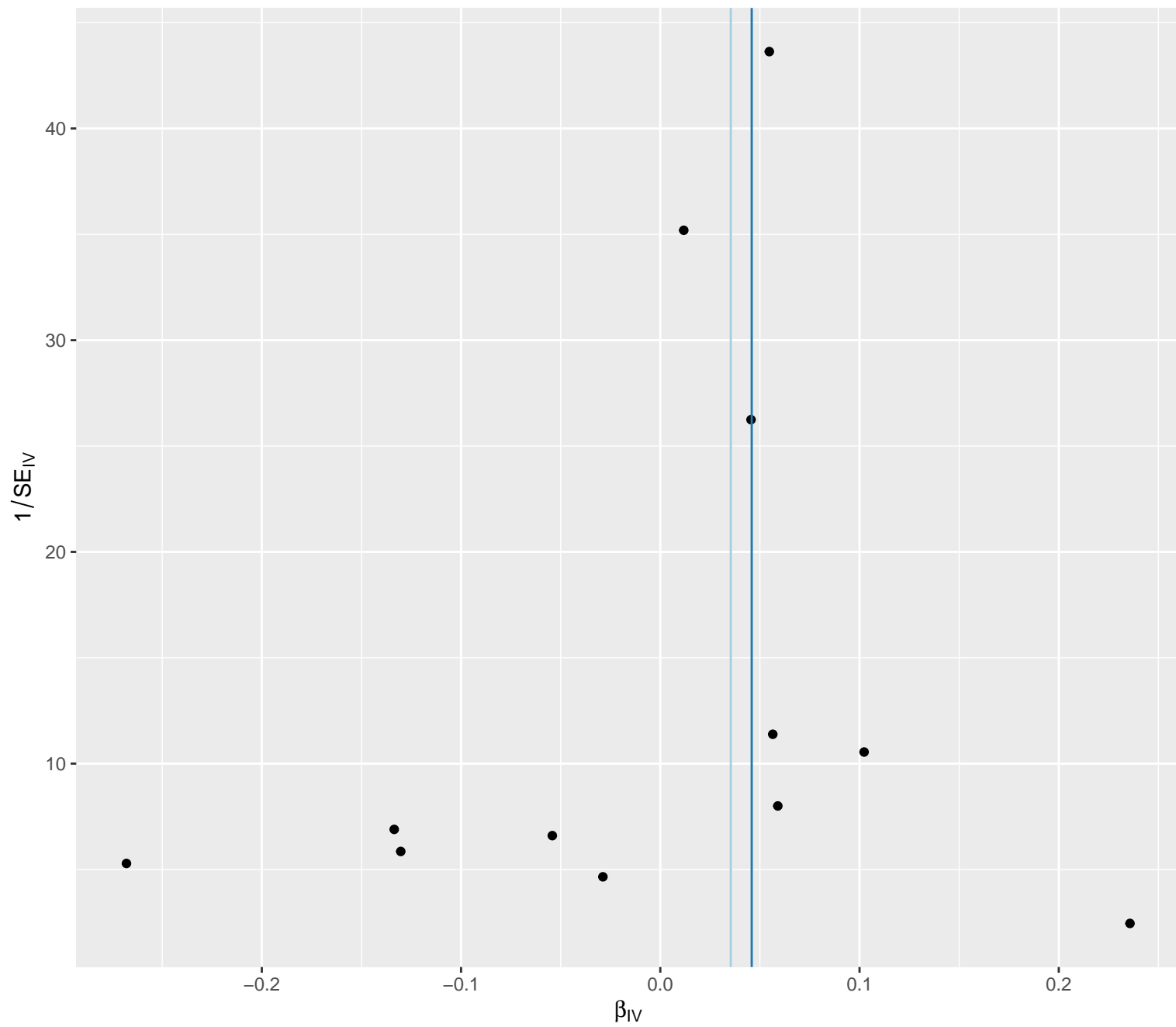

Supplement: S3 File — (ZIP) [file pone.0309088.s003.zip › S3 Fig/ebi-a-GCST90001917/funnelplot.pdf]

# MR Method

- Inverse variance weighted
- MR Egger

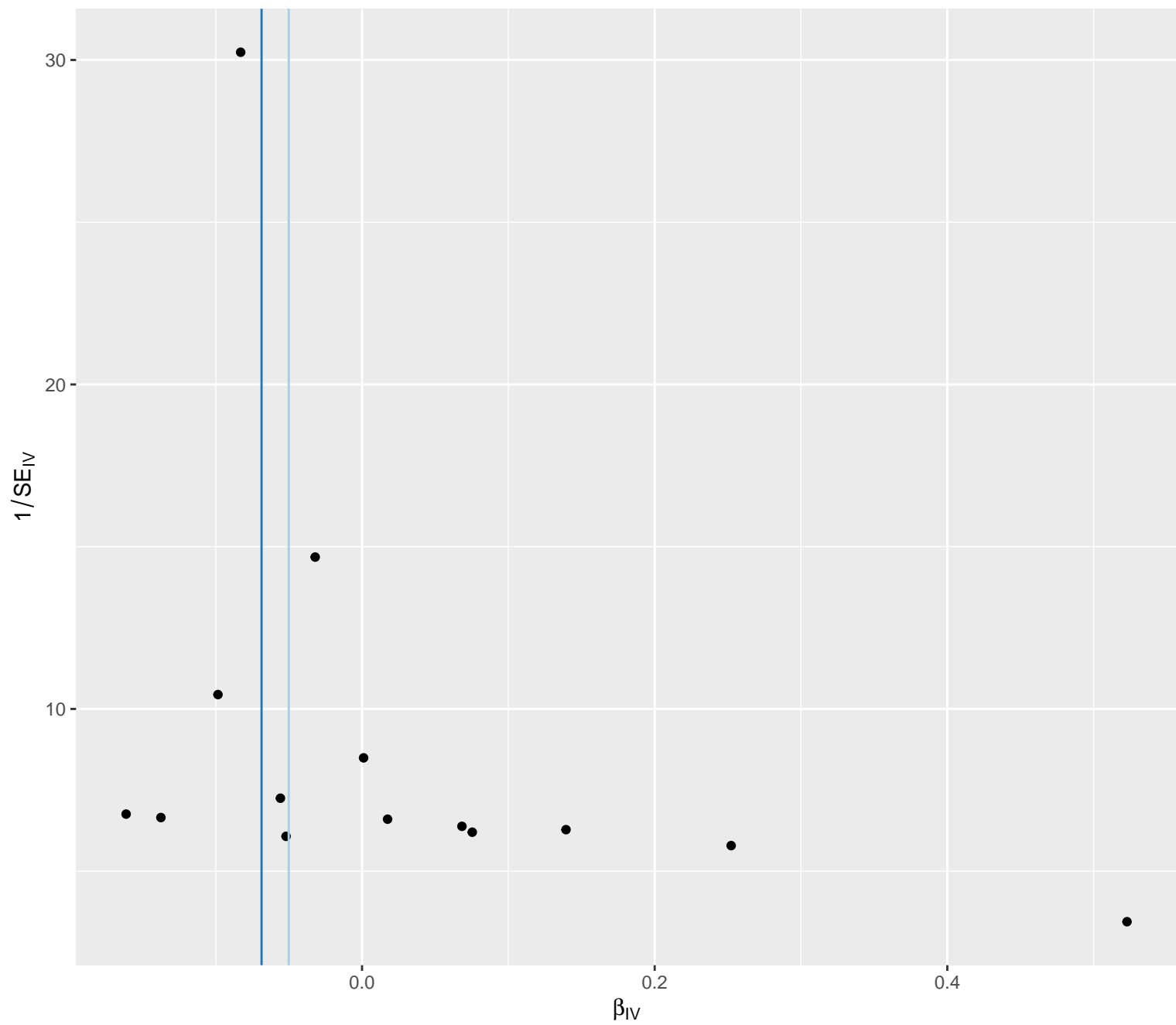

Supplement: S3 File — (ZIP) [file pone.0309088.s003.zip › S3 Fig/ebi-a-GCST90001590/funnelplot.pdf]

# MR Method

- Inverse variance weighted
- MR Egger

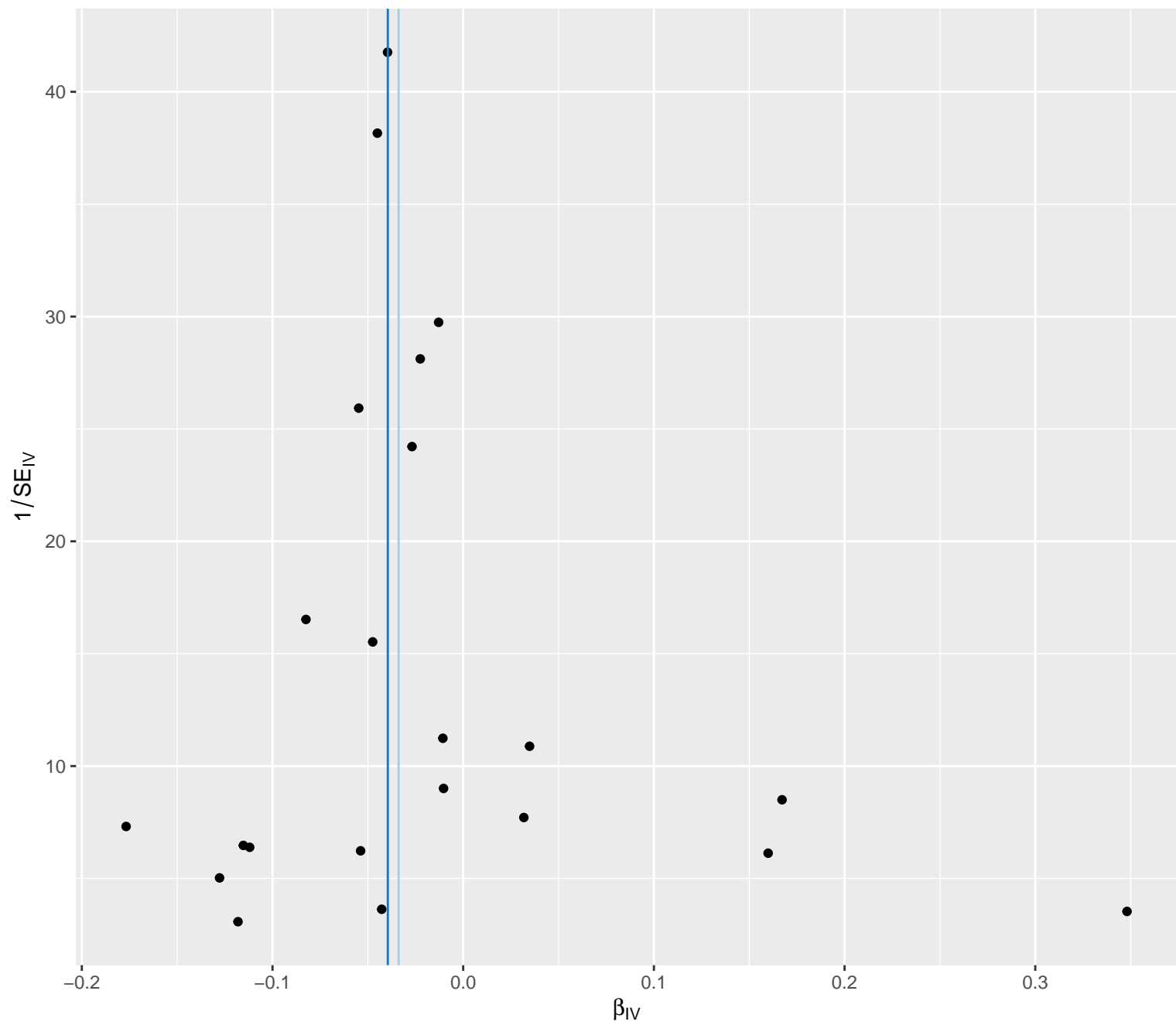

Supplement: S3 File — (ZIP) [file pone.0309088.s003.zip › S3 Fig/ebi-a-GCST90001992/funnelplot.pdf]

# MR Method

- Inverse variance weighted
- MR Egger

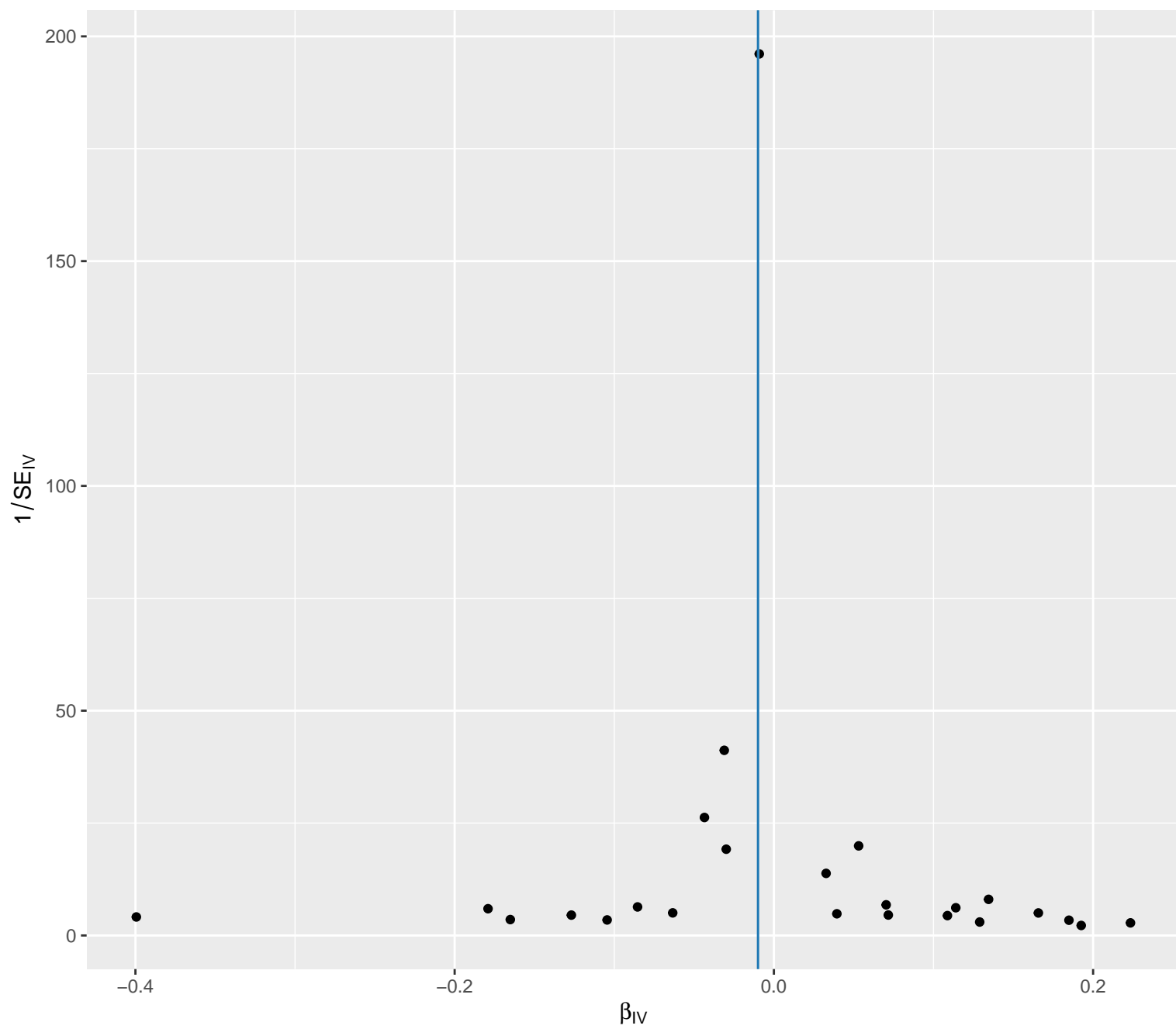

Supplement: S3 File — (ZIP) [file pone.0309088.s003.zip › S3 Fig/ebi-a-GCST90001553/funnelplot.pdf]

# MR Method

- Inverse variance weighted
- MR Egger

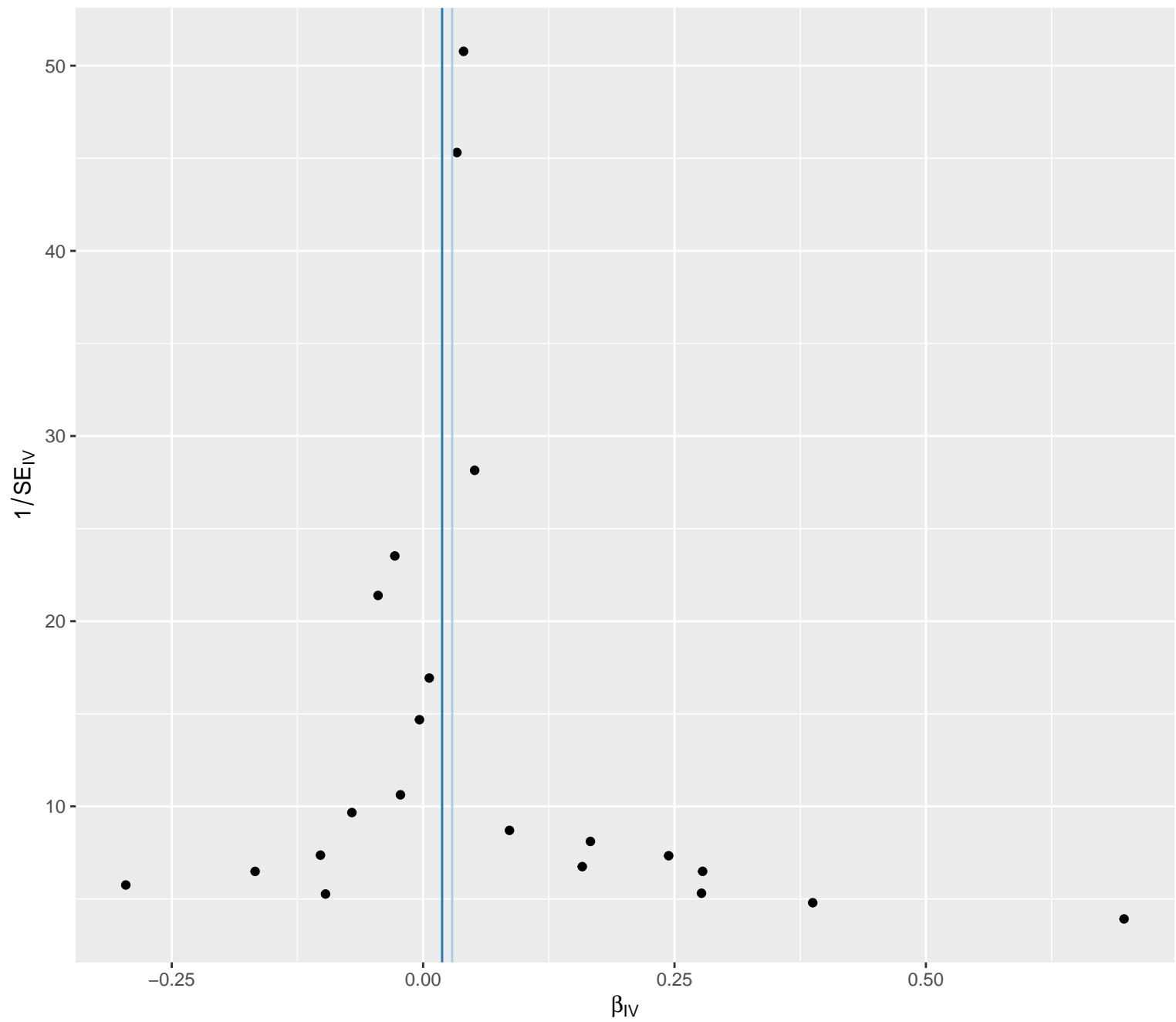

Supplement: S3 File — (ZIP) [file pone.0309088.s003.zip › S3 Fig/ebi-a-GCST90001481/funnelplot.pdf]

# MR Method

- Inverse variance weighted
- MR Egger

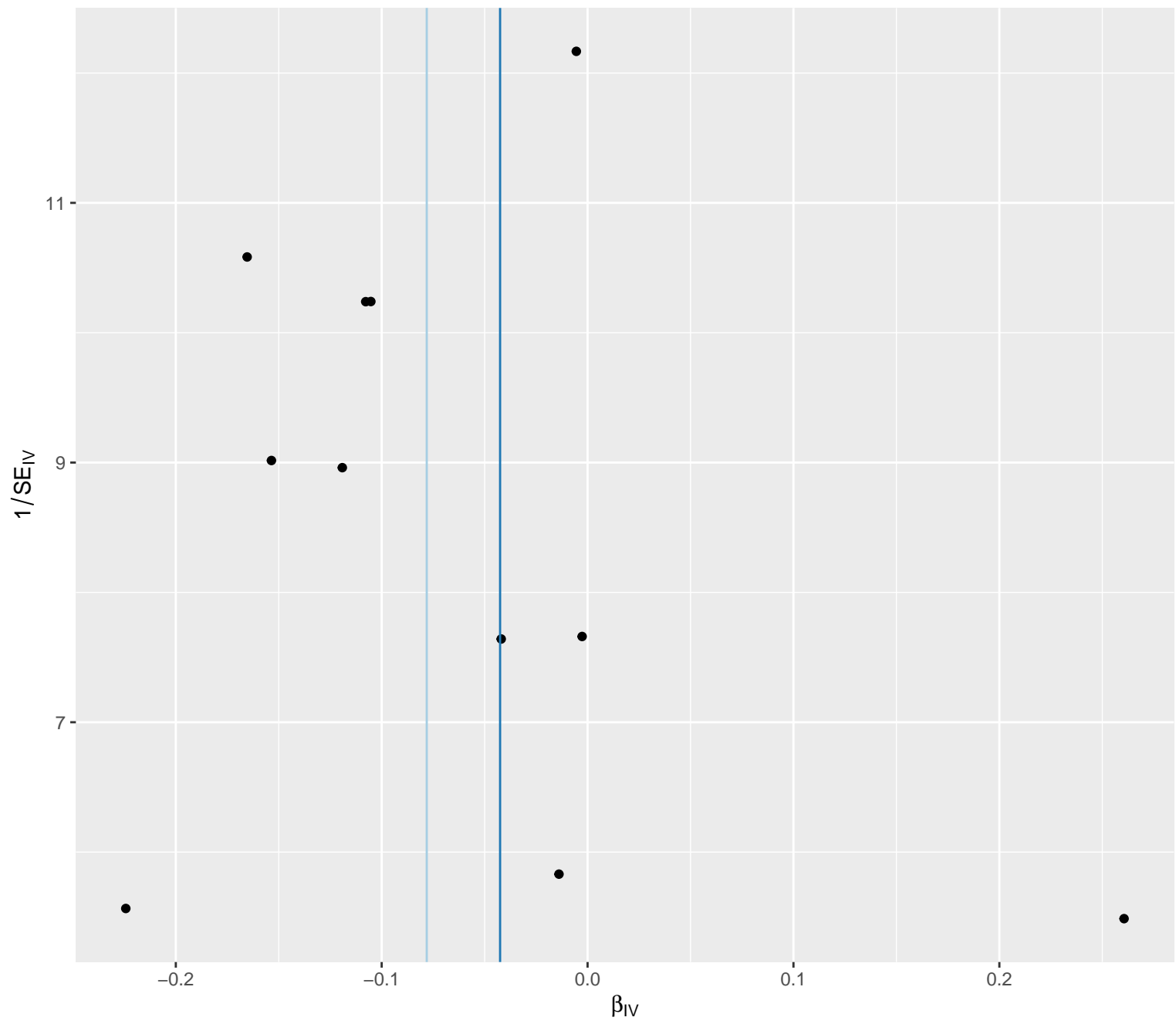

Supplement: S3 File — (ZIP) [file pone.0309088.s003.zip › S3 Fig/ebi-a-GCST90002062/funnelplot.pdf]

# MR Method

- Inverse variance weighted
- MR Egger

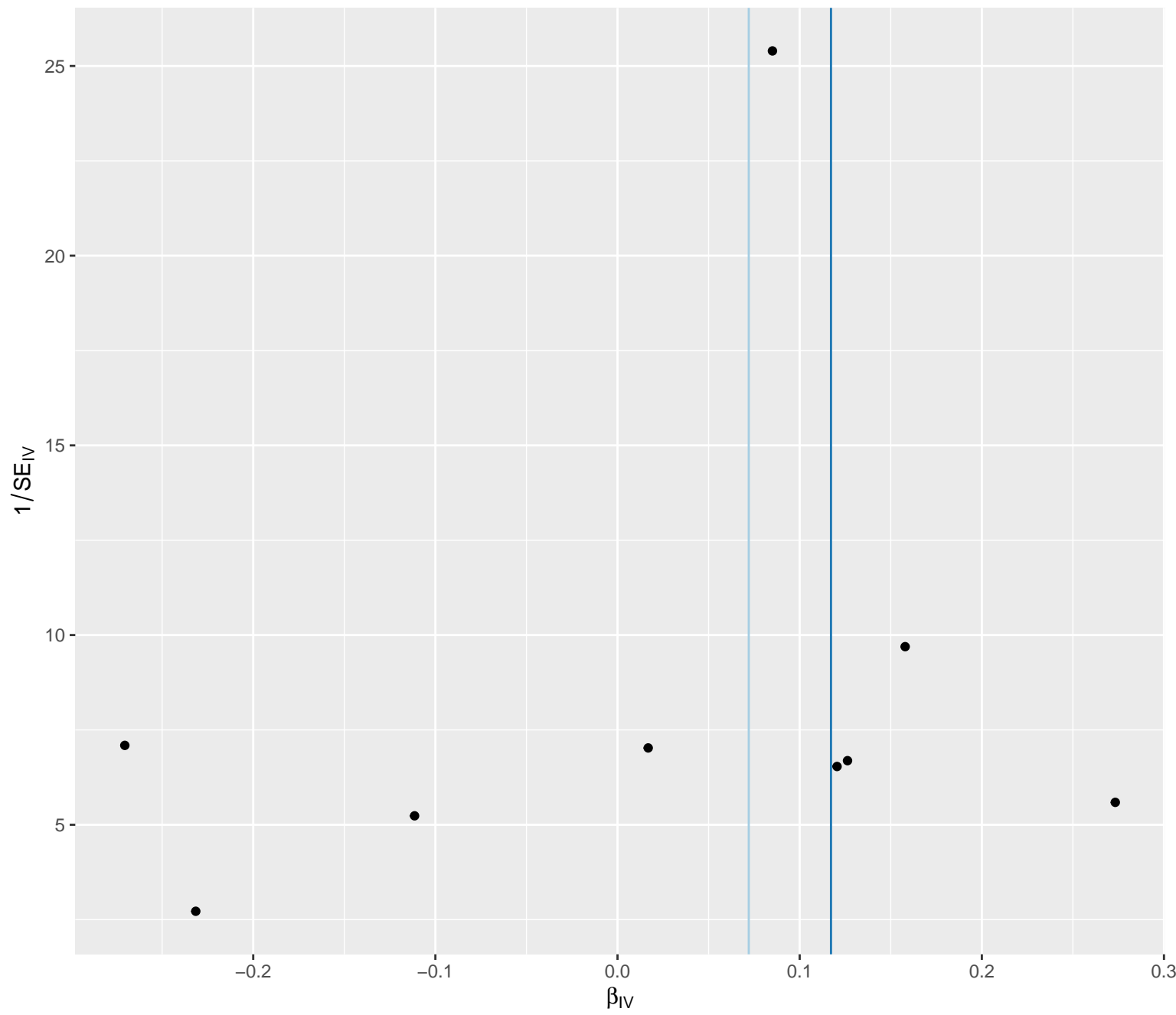

Supplement: S3 File — (ZIP) [file pone.0309088.s003.zip › S3 Fig/ebi-a-GCST90001648/funnelplot.pdf]

# MR Method

- Inverse variance weighted
- MR Egger

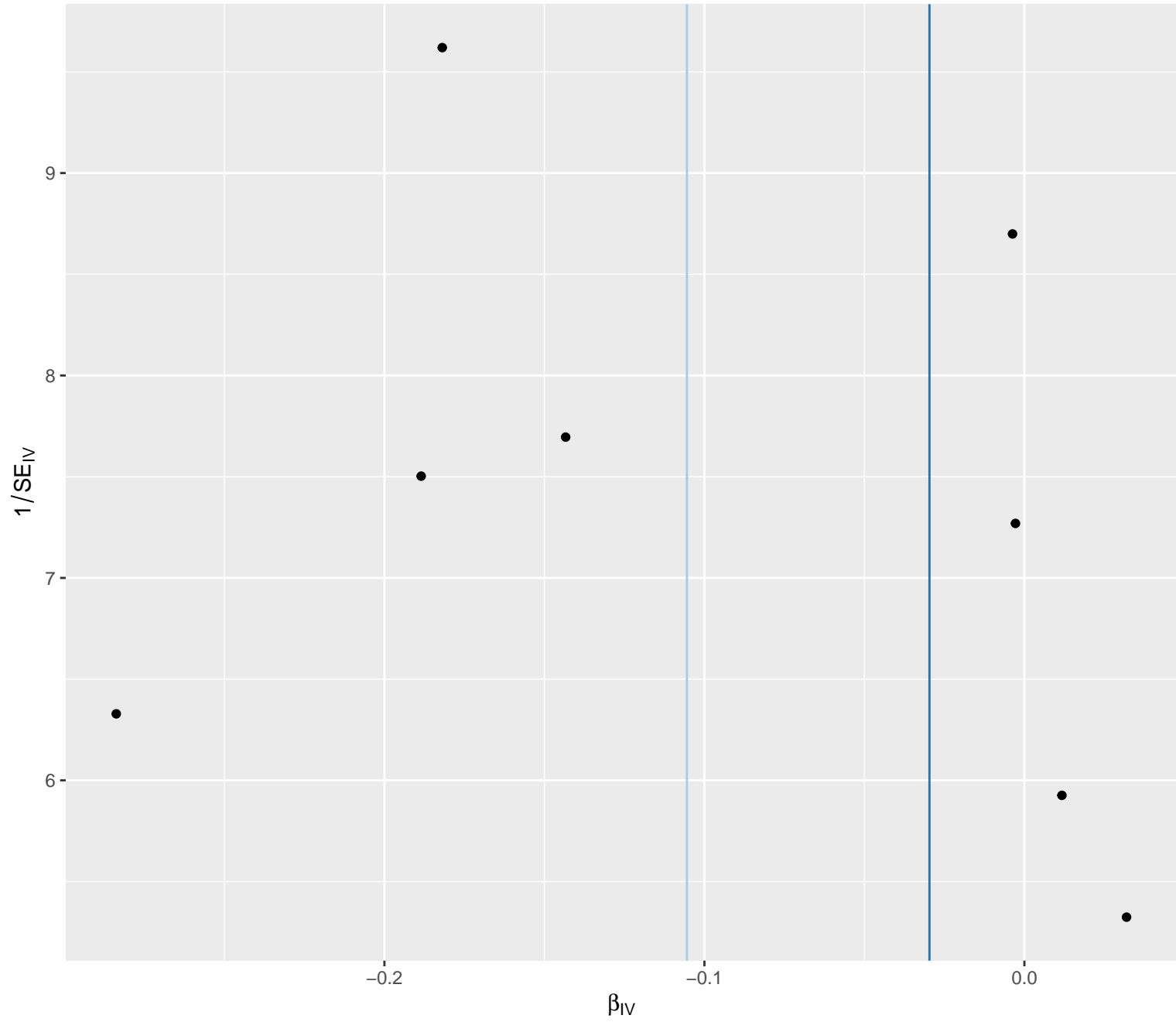

Supplement: S3 File — (ZIP) [file pone.0309088.s003.zip › S3 Fig/ebi-a-GCST90002064/funnelplot.pdf]

# MR Method

- Inverse variance weighted
- MR Egger

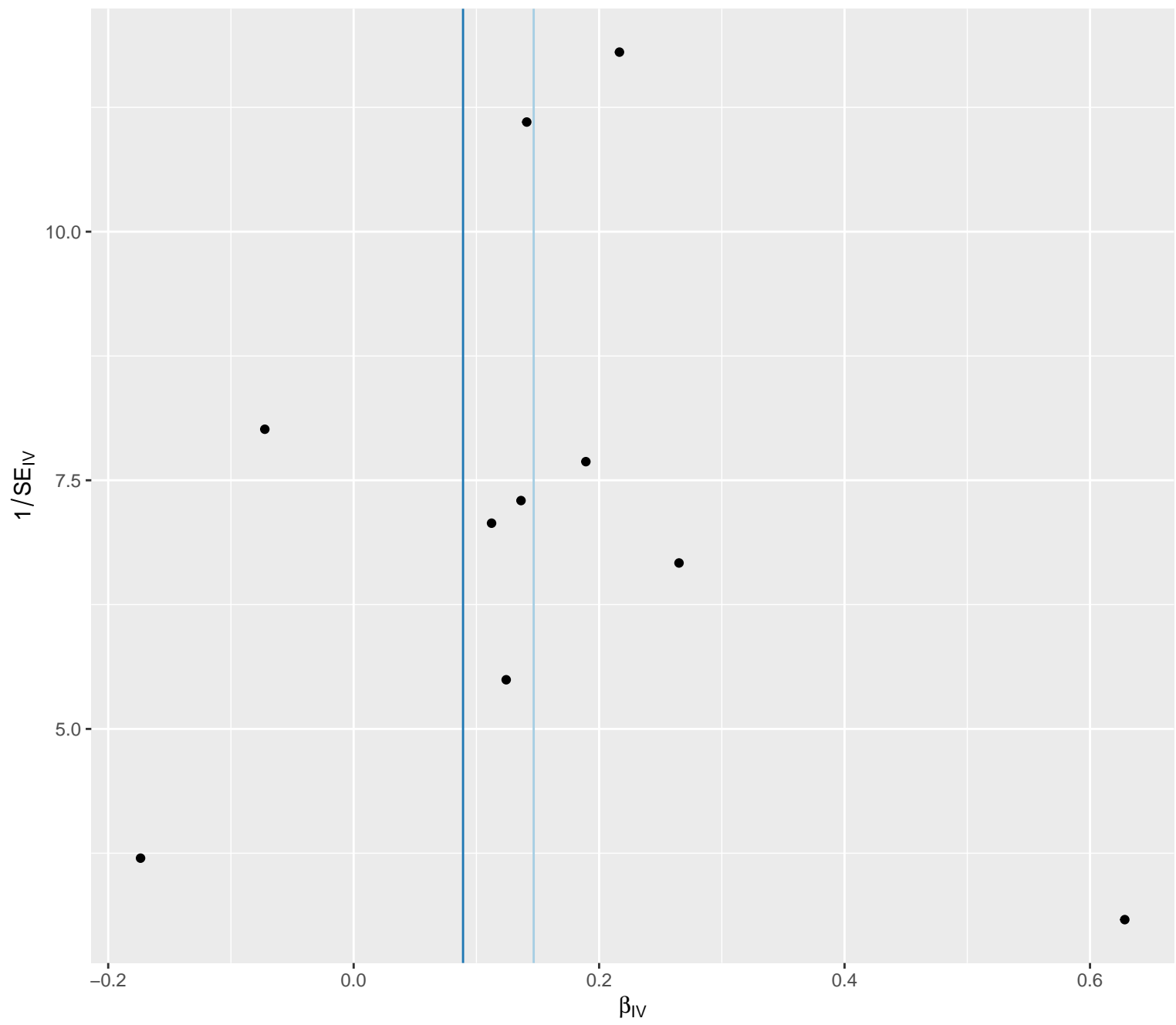

Supplement: S3 File — (ZIP) [file pone.0309088.s003.zip › S3 Fig/ebi-a-GCST90001411/funnelplot.pdf]
