# Supplementary material for: From genes to reproductive health: Immune cell influences on abortion
Source: PLoS One. 2024 Oct 10;19(10):e0309088. doi: 10.1371/journal.pone.0309088 (PMC11466425; doi:10.1371/journal.pone.0309088)
Supplement: S2 File — (ZIP) [file pone.0309088.s002.zip › S2 Fig/ebi-a-GCST90001455/scatter.pdf]

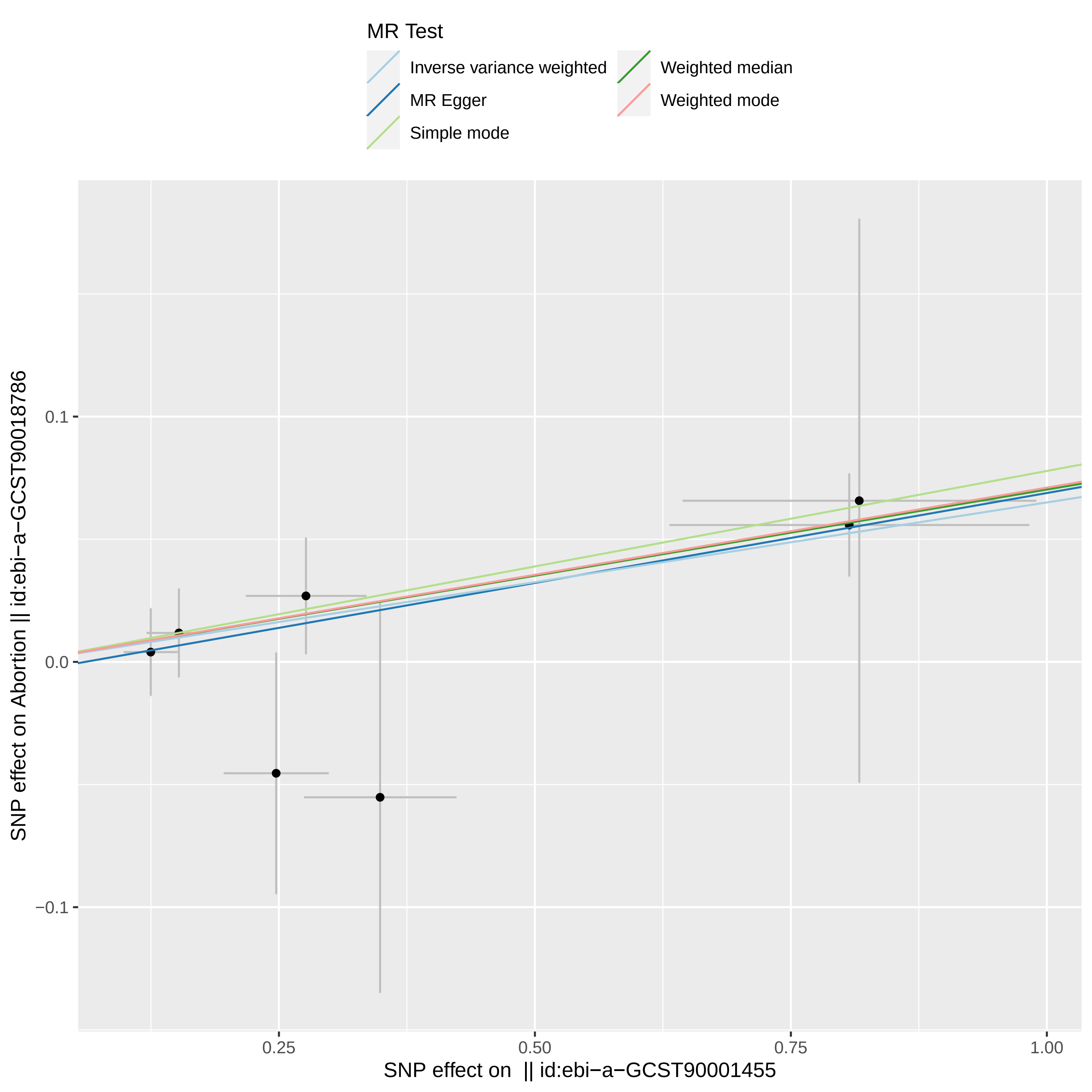

MR Test
Inverse variance weighted
MR Egger
Simple mode
Weighted median
Weighted mode
0.1
0.0
−0.1
0.25
0.50
0.75
1.00
SNP effect on Abortion || id:ebi−a−GCST90018786
SNP effect on || id:ebi−a−GCST90001455
