# Supplementary material for: From genes to reproductive health: Immune cell influences on abortion
Source: PLoS One. 2024 Oct 10;19(10):e0309088. doi: 10.1371/journal.pone.0309088 (PMC11466425; doi:10.1371/journal.pone.0309088)
Supplement: Attachment — Submitted filename: Response to Reviewers.doc [file pone.0309088.s008.doc]

Dear Editor and Reviewers,

On behalf of all the contributing authors, I would like to express our sincere appreciation of the reviewers’ constructive comments concerning our article entitled “From genes to reproductive health: immune cell influences on Abortion” (ID PONE-D-24-01489).

The feedback provided was highly valuable, contributing to the enhancement of our manuscript. In response to the reviewers' comments, we have amended and supplemented the document, adding further explanations to bolster the credibility of our results. In this revised version, all modifications to our manuscript are highlighted in red text within the file. We address each of the aforementioned issues in detail below.

To Reviewer 1,

1.We fully agree with your view. We have continued to read a substantial amount of literature related to immune cells and miscarriage, providing a more extensive and in-depth background description in the introduction.

2.Thank you for your insightful comments and suggestions. We have thoroughly reviewed the manuscript and corrected any typographical errors. Additionally, we have added more content for discussion, significantly enhancing both the academic rigor and readability of the paper.

3.Thank you for your careful review of the entire manuscript. Based on your suggestions, we have provided a more concise explanation in the conclusion section.

To Reviewer 2,

1.Thank you very much for pointing out this issue. We apologize for the oversight; schizophrenia was a typographical error. We have since removed it.

2.I fully understand your concerns. Following your suggestions, we have meticulously arranged the related content and information in the attachments to facilitate easier reading. We have examined the full text and the associated images. Additionally, we have made modifications to certain parts where the wording was inappropriate.

3.Thank you for your constructive feedback. We have almost entirely rewritten the discussion section and reviewed a substantial amount of literature. This has made our paper more rigorous and academically meaningful. Once again, thank you for your suggestions; they have driven us to improve significantly.

To Editor,

1.Thank you very much for your hard work. We have made revisions according to the format requirements of your journal. If there are any other oversights on our part, please contact us immediately for corrections.

2.Thank you for the reminder. The data on miscarriage is sourced from [https://www.ebi.ac.uk/gwas/publications/34594039]. The data on immune cells is sourced from [https://www.ebi.ac.uk/gwas/publications/32929287]. All data is publicly available for download. Additionally, we have supplemented the data sources in the main text as well.

3.We have revised the content according to your formatting requirements.

4.Thank you for the reminder. Supplementary Table 3 was a typographical error, and we have removed it.
